# Integrated transcriptome and plant growth substance profiles to identify the regulatory factors involved in floral sex differentiation in *Zanthoxylum armatum* DC

**DOI:** 10.3389/fpls.2022.976338

**Published:** 2022-09-02

**Authors:** Wenkai Hui, Jiangtao Fan, Xianzhi Liu, Feiyan Zhao, Tasheen Saba, Jingyan Wang, Aimin Wu, Xuebin Zhang, Junli Zhang, Yu Zhong, Gang Chen, Wei Gong

**Affiliations:** ^1^Key Laboratory of Ecological Forestry Engineering of Sichuan Province, College of Forestry, Sichuan Agricultural University, Chengdu, China; ^2^State Key Laboratory for Conservation and Utilization of Subtropical Agro-Bioresources, College of Forestry and Landscape Architecture, South China Agricultural University, Guangzhou, China; ^3^State Key Laboratory of Cotton Biology, Department of Biology, Institute of Plant Stress Biology, Henan University, Kaifeng, China

**Keywords:** *Zanthoxylum armatum*, transcriptome, phytohormone, sucrose and starch, floral sex differentiation

## Abstract

*Zanthoxylum armatum* is a prominent plant for food industries. Its male flowers often occur in gynogenesis plants; however, the potential mechanism remains poorly understood. Herein, a total of 26 floral sex differentiation stages were observed to select four vital phases to reveal key factors by using RNA-seq, phytohormones and carbohydrates investigation. The results showed that a selective abortion of stamen or pistil primordia could result in the floral sex differentiation in *Z. armatum*. Carbohydrates might collaborate with cytokinin to effect the male floral differentiation, whereas female floral differentiation was involved in SA, GA_1_, and ABA biosynthesis and signal transduction pathways. Meanwhile, these endogenous regulators associated with reproductive growth might be integrated into ABCDE model to regulate the floral organ differentiation in *Z. armatum*. Furthermore, the 21 crucial candidates were identified in co-expression network, which would contribute to uncovering their roles in floral sex differentiation of *Z. armatum* in further studies. To the best of our knowledge, this study was the first comprehensive investigation to link floral sex differentiation with multi-level endogenous regulatory factors in *Z. armatum*. It also provided new insights to explore the regulatory mechanism of floral sex differentiation, which would be benefited to cultivate high-yield varieties in *Z. armatum*.

## Introduction

The plant characteristics involved in flowering are an important biological basis for the breeding of high-yield varieties, especially for the fruit-producing economic forest plants. The differentiation and development of male and female flowers often have a direct effect on their fruit yield and subsequent economic benefits. Therefore, it was crucial to explore the endogenous regulatory factors associated with floral sex differentiation and uncover their corresponding regulation mechanism, which could be benefited to cultivate high-yield economic forestry varieties.

*Zanthoxylum*, belonged to Rutaceae, is a very famous condiment and medicine tree species in southwest China and some other regions of Asia, such as Japan, Southern Korea, India, Pakistan, and Nepal (Hui et al., 2021). *Zanthoxylum armatum* DC, commonly known as Green prickly ash because of its fruit color, is not only enriched with the special numbing substances, but also attached with some fragrance, all of which make it one of the most popular species in recent contemporary public diets (Luo et al., 2021). As one of the key spices, the fruit of *Zanthoxylum* had generated billions of dollars in China as mentioned on huajiao.cn website (Hui et al., 2021). Meanwhile, as one of important medicinal trees, *Zanthoxylum* has also contributed to the economic development in certain countries. Because its fruit pulp could be used to treat some skin diseases, fever, and toothache among other ailments (Hui et al., 2021). It was worth to note that a majority of *Z. armatum* plants were gynogenetics, and reproduced parthenogenetically. Meanwhile a few trees were male or monoecious plants, even the bisexual flowers could be occasionally observed in *Z. armatum*. Interestingly, if male flowers appeared in gynogenesis plants, which could be increased year by year until the trees were transformed into male plants. Thus, some researchers even suggested that the male flower looks like a flower yellow disease. Recently, three publications reported some viruses might be correlated with the male flower development (Cao et al., 2019, 2021; Xu et al., 2021), however, the floral gender was not changed when the viruses were re-introduced into female plants (Cao et al., 2019). Therefore, insufficient research has been conducted on the potential regulatory factors of floral sex differentiation in *Z. armatum*, furthermore, its underlying genetic mechanisms have not been clarified.

To date, there is no molecular regulatory model that could be used to explain the different plant sex determination mechanisms in various plants, especially in the dioecious plants. However, we were able to confirm that a few determinative genes often play a crucial role in the pistil and stamen differentiation process (Feng et al., 2020). The absence of cytokinin-associated pistillate suppressor, *ARPT3*, led to the formation of bisexual flowers, which may regulate floral sex differentiation by the selective abortion of pistils or stamens in grape (Feng et al., 2020). Similarly, the cytokinin response factor family annotated with pistil suppressor gene *SyGI* (*Shy Girl*) could also regulate the floral sex differentiation in kiwifruit, and a mutation of *SyGI* could induce the production of unisexual kiwifruit (Akagi et al., 2018). Later on, a stamen-promoting factor *FrBy* (*Friendly boy*) along with *SyGI* was reported to co-regulate the pistil differentiation (Akagi et al., 2019). In addition, the *ARR17* played its function as a sex switch to regulate the floral differentiation in European aspens (Müller et al., 2020). The *FERR*, *FERR-R* and *MSL* genes jointly determined the gender differentiation process in *Populus deltoides* (Xue et al., 2020). The sex-determining genes have been demonstrated to regulate the male and female floral formation through the pathways associated with flower organ differentiation (Wang S. et al., 2019). Recently, it has been reported that the floral sex differentiation could be regulated by plant endogenous hormones, especially cytokinins, gibberellins and jasmonic acids (Shi and Vernoux, 2022). In addition, energy metabolism pathway is crucial for the flowering regulation (Hao et al., 2021), as sufficient nutrients not only provide the energy basis for flower bud differentiation, but also supply the precursors for the biosynthesis of plant endogenous hormones that can be used as signal molecules to regulate the expression abundance of transcription factors of floral sex determination (Liu et al., 2019). Furthermore, the various regulatory signals directly or indirectly interact with the floral organ identity genes to control male and female floral formation, such as the ABC model genes (Irish, 2017). Thus, a complex regulatory network is involved in the flower sex differentiation, which comprises of a variety of external environment and endogenous pathways. Nonetheless, these crucial genes and their regulatory mechanism have not yet been reported in *Z. armatum*.

Recently, the genomic dataset of *Z*. *armatum* was released that supplied a wealth of information to explore the key genes associated with floral sex differentiation (Wang M. et al., 2021). In order to conduct morphological observations in *Z. armatum*, the male and female floral samples at different developmental stages were collected in the present study. Then, through observation using stereoscopy, paraffin section, as well as scanning electron microscopy (SEM), four vital periods of floral sex differentiation were identified. To explore the potential regulatory genes and crucial pathways, the 24 transcriptomic profiles of floral sex differentiation stages were obtained. Meanwhile, the 12 endogenous plant hormones were investigated using HPLC-ESI-MS/MS to uncover the regulatory events of floral sex differentiation in *Z. armatum*. Furthermore, the significantly enriched DEGs were carried out by qRT-PCR analysis using 15 samples related to male and female floral differentiation process. All of the information was integrated to reveal the underlying genetic mechanisms involved in floral sex differentiation in *Z. armatum* DC. This study is the first comprehensively investigation to link floral sex differentiation with multi-level endogenous regulatory factors in *Z. armatum*. Thus, the findings of the present study provide the valuable information for future functional studies involving the floral sex differentiation.

## Materials and methods

### Morphological observations of male and female floral development process

The dioecious trees of five-year-old *Z. armatum* were selected as the experimental material in this study, which was planted at a forestry trial base of Sichuan Agricultural University (30.60°N, 103.65°E) in Chengdu City, Sichuan Province, China. The environment and conditions are described in our previous study (Hui et al., 2020). The male and female floral samples were harvested approximately every 7 days at 9:00 to 10:00 a.m. from September 2020 to March 2021, which contained the main periods from undifferentiated inflorescence buds to full maturated male or female flowers. All the samples were observed by stereoscopy and paraffin section (Hui et al., 2018), and then flash-frozen in liquid nitrogen and stored at −80°C until further use for RNA extraction. The floral buds were, respectively, collected from 10 individual male or female plants. After flowering, the floral sex observation was conducted to re-confirm the gender of the trees. And then, the floral samples of the confirmed trees were mixed together as a biological replicate in each stage, and three independent biological replicates were conducted in this study. Additionally, 15 floral buds were used to confirm the differentiation stage and the connections between floral exterior characters and interior differentiation stages. Meanwhile, SEM observation were carried out and four vital phases of male and female floral differentiation were identified as: undifferentiated male primordia (M1), male primordia initiation (M2), male primordia differentiation (M3), male development and maturation (M4), undifferentiated female primordia (F1), female primordia initiation (F2), female primordia differentiation (F3), female development and maturation (F4).

### Total RNA extraction and transcriptome sequencing

Total RNA from four vital phases was separately extracted using Fast Pure Plant Total RNA Isolation Kit (Vazyme, Nanjing, China). The quantity and purity of total RNA were investigated by agarose gel electrophoresis and Nanodrop 2,100 (Agilent, United States). The 24 cDNA libraries, including M1_1, M1_2, M1_3, M2_1, M2_2, M2_3, M3_1, M3_2, M3_3, M4_1, M4_2, M4_3, F1_1, F1_2, F1_3, F2_1, F2_2, F2_3, F3_1, F3_2, F3_3, F4_1, F4_2 and F4_3, were constructed and sequenced using Illumina HiSeq™ 4,000 platform at Novogene Bioinformatics Technology Co. Ltd. (Beijing, China). After quality control, the clean reads were aligned with the genomic dataset of *Z. armatum* obtained in Fig Share database^1^ (Wang M. et al., 2021). Then, the HTSeq software was used to calculated the expression abundance of each gene and normalized to the expected number of fragments per kilobase of transcript sequence per million fragments mapped (FPKM; Liu et al., 2021).

### Gene functional annotation and DEG identification

To comprehensively obtain the functional information, all genes were aligned by BLASTX against NCBI nonredundant protein sequence (NR), NCBI nucleotide sequence (NT), Protein family (Pfam), KEGG Orthology (KO) and euKaryotic Ortholog Groups (KOG) databases with a threshold E-value of 10^−5^. Meanwhile, the functional annotation and classification of all genes were also conduct based on Gene Ontology database^2^ (GO) and Kyoto Encyclopedia of Genes and Genomes database^3^ (KEGG).

In this study, all samples were divided into 12 comparisons (M2*vs.*M1, M3*vs.*M2, M3*vs.*M1, M4*vs.*M3, F2*vs.*F1, F3*vs.*F2, F3*vs.*F1, F4*vs.*F3, M1*vs.*F1, M2*vs.*F2, M3*vs.*F3, and M4*vs.*F4) to comprehensively identify the important genes associated with male floral differentiation, female floral differentiation and male *vs.* female floral differentiation. All the up- or down- regulations in the following description represent in the first comparison component. The differential expression genes (DEGs) among each comparison were detected by DESeq2 package (1.16.1). Foldchange (FC) was the gene expression difference between different samples, and the threshold was set as |log2 (FC)| > 1 and *p*-value < 0.05 to identify the DEGs. The KEGG enrichment was carried out to classify the significant pathways of DEGs, and the threshold was set as the corrected *p*-value < 0.05. The transcription factors were identified by iTAK_v1.6a software (Yi et al. 2016) The BLASTX alignment was carried out to annotate the DEG information with TAIR11 database.^4^ The expression abundance of DEG was displayed as heat map by TBtools software (Chen et al., 2020). Moreover, the network of vital genes and various plant growth substances was performed by Cytoscape software.

### Extraction and detection of phytohormones and sugar substances

The same samples as RNA-seq were used to investigate the content of various plant hormones in this study. 100 mg fresh samples of four vital phases with three biological replicates were collected and immediately frozen with liquid nitrogen to detect the 12 phytohormones using HPLC-MS/MS platform, including salicylic acid (SA), jasmonic acid (JA), Jasmonate isoleucine (JA-ILE), Plant dienic acid reductase (OPDA), Abscisic acid (ABA), Gibberellin acid 1 (GA_1_), Gibberellin acid 3 (GA_3_), Gibberellin acid 4 (GA_4_), Indole acetic acid (IAA), Trans-zeatin nucleoside (TZR), Zeatin (ZT), and aminocyclopropanecarboxylic acid (ACC, the biosynthetic precursor of ethylene). The investigation of each phytohormone was conduct as previously described by Xin et al. (2020). The regression equation of 12 phytohormones were obtained by plotting the detected peak areas of their corresponding concentrations (Supplementary Table 1). Meanwhile, the 100 mg of similar samples were also collected to, respectively, investigate the concentration of total sugar, soluble sugar, trehalose, and sucrose content using Sugar Content Assay Kit (Solarbio, Beijing, China) as described by Pan et al. (2022). Three biological replicates of each sample were performed to estimate these plants physiological index.

### Quantitative real-time PCR analysis

To validate the transcriptome results, a total of 47 objects (15 DEGs within 11 comparisons) were selected for quantitative real-time PCR (qRT-PCR) analysis using the similar samples of RNA-seq. These genes were randomly chosen due to their high FC and crucial functions identified in this study. Three biological replicates were conduct with three technological replications for each gene. Moreover, the DEGs associated with sucrose synthesis and metabolic pathways, as well as phytohormone biosynthesis and signal transduction pathways were also selected for qRT-PCR analysis using 15 samples related to male and female floral differentiation process.

The total RNA of each sample was extracted using FastPure Plant Total RNA Isolation Kit (Vazyme, Nanjing, China) according to the manufacturer’s protocol, and the cDNA was obtained using PrimeScript® II first Strand cDNA Synthesis Kit (TaKaRa, Japan). The specific primers were designed by Primer Premier 5.0 with amplified PCR products from 80 to 300 bp (Supplementary Table 2). The qRT-PCR was referred to our previous experimental operation in a Bio-Rad CFX96™ system (Bio-Rad, America; Hui et al., 2020), and *ZaUBQ* was selected as internal control according to the previous study (Fei et al., 2018). The first female sample was used as control to estimate the relative expression of the DEGs in this study, and the relative expression level of the genes were calculated using 2^–ΔΔCt^ method in different samples (Hui et al., 2021).

### Statistical analysis

In the present study, the statistical analysis was conduct using the R software for the phytohormone and qRT-PCR data. The significance among each sample was estimated by Duncan’s multiple range tests, and the level of significance was set to *p* < 0.05, and GraphPad Prism 5 was used to draw the figures.

## Results

### Morphological characters of male and female floral differentiation

**Figure 1:**
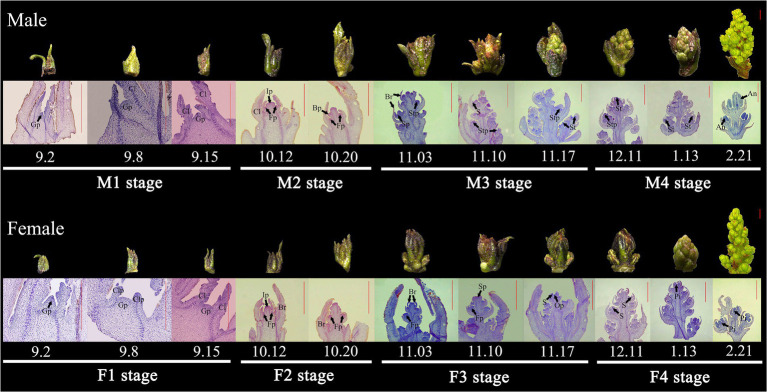
The vital periods of floral sex differentiation and organogenesis in *Z. armatum*. An, anther; Br, bract; Bp, bract primordium; Cl, compound leaf; Clp, compound leaf primordium; Fp, floral primordium; Gp, floral growing point; Ip, inflorescence primordium; Op, ovule primordium; Pi, pistil; S, sepal; Sp, sepal primordium; St, stamen; Stp, stamen primordium. The bar was 1 mm, and the decimals was the date to collect the samples. For example, 9.2 present the 2 September.

To comprehensively reveal the morphological characters of floral sex differentiation process in *Z. armatum*, a total of 26 samples were harvested, which covered the main periods from undifferentiated inflorescence buds to full maturated male or female flowers (Supplementary Figures 1, 2). Based on the morphological identification conducted by stereoscopy, paraffin section and SEM observation, the male and female floral differentiation were, respectively, divided into four stages in this study (Figure 1; Supplementary Figures 1–4). From September to early October, the inflorescence was located in the most initial primordial period (M1 or F1 stage), which was the beginning of the transition from vegetative to reproductive growth. At this time, the inflorescences were small and born in the axils of fruited branches, the growth cone was slightly uplifted with the light green and triangular surface. In the following days of October, the inflorescence continued to differentiate and the primary flower buds gradually become more distinguishable (M2 or F2 stage), however, the primordium of stamen and pistil flowers were still not visible. The inflorescence primordium was elongated and thickened at M2 and F2 stage, and some spherical projections gradually occurred around the sides of the main axis. During November to early December (M3 or F3 stage), the male and female floral primordium was triggered and the inflorescence was continuously expanded with brown-red spot and some fine hair. At this stage, the axis of inflorescence was elongated to form multiple vesicular projections, the male and female floral bud can be easily distinguished. Subsequently, the male and female flower continuously differentiated, the stamens and pistils were observed and the complete stamens and ovule were formed in M4 and F4 stage. After the cold winter, the flower buds entered into the rapid growth periods with the rise of temperature. The inflorescence sharply expanded in 2 months, the size of floral bud rapidly increased, and the ovary primordium grew continuously with the distinct stigma at the top of female flower (Supplementary Figure 2). Meanwhile, the yellow stamens were visible in male flowers, and a slight collapse was observed in the center of male flowers leading to the formation of a cavity (Supplementary Figure 1).

Thus, the four vital periods of male and female floral differentiation were selected based on their morphological characters (Figure 1), including undifferentiated male primordia (M1), male primordia initiation (M2), male primordia differentiation (M3), male development and maturation (M4), undifferentiated female primordia (F1), female primordia initiation (F2), female primordia differentiation (F3), and female development and maturation (F4). All of these samples were performed RNA-seq to detect the crucial genes associated with floral sex differentiation.

### Transcriptome sequencing and assembly of different cDNA libraries

The total RNA of 24 floral samples, including M1-M4 and F1-F4, were isolated to construct the transcriptome profiles in *Z. armatum*. The quality of the RNA was evaluated by the RIN (6.90–10.00; Supplementary Table 3). After quality control, 6.12–7.00 G of clean bases were produced from 24 cDNA libraries by RNA-seq in this study (Supplementary Table 4). The error rates of transcriptome profiles were only approximately 0.03%, all the Q30 values were greater than 91.22%, and the GC content was between 43.85 and 44.50% in each sample. When the RNA-seq data was aligned with the genomic profiles of *Z. armatum*, the total map of 78.46 to 85.54% was observed (Supplementary Table 5), and the mapped exon was more than 77% (Supplementary Table 6). In addition, the principal component analysis (PCA) revealed that the three independent biological replicates of each sample presented good reproducibility (Supplementary Figure 5). All the results indicated that the high-quality RNA-seq datasets were obtained and can be used to conduct the following analysis.

### Regulatory patterns of male floral differentiation

A total of 2,866, 3,692, 1,468, and 4,363 DEGs were identified in M2*vs.*M1, M3*vs.*M1, M3*vs.*M2, and M4*vs.*M3 (Supplementary Figure 6). Because the floral primordium was triggered in M1 and M2 stages, the relative high correlation coefficient was obtained among these samples (Supplementary Figure 7). To obtain more significantly DEGs, the M3*vs.*M1 and M4*vs.*M3 were used to isolate the key pathways involved in male floral differentiation in the present study, although the M2*vs.*M1 and M3*vs.*M2 were enriched in the genes involved in plant hormone signal transduction and carbohydrate metabolism pathways.

**Figure 2:**
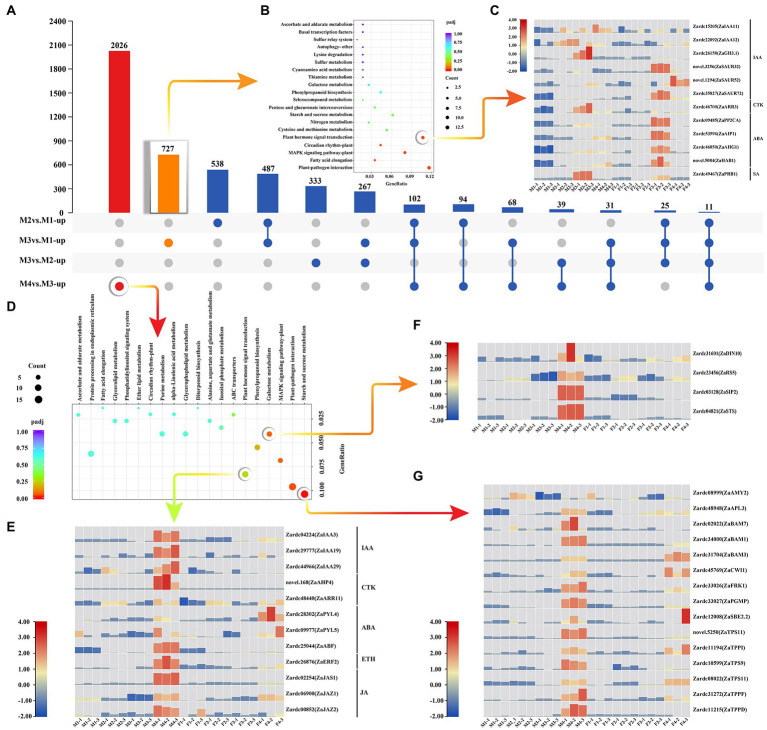
The key genes selection involved in male floral differentiation. **(A)** The upset statistics of up-regulated DEGs among male floral samples. **(B)** The KEGG enrichment of the up-regulated DEGs in M3*vs.*M1. **(C)** The heat map of the DEGs associated with plant hormone signal transduction which was the significant enrichment pathway in M3*vs.*M1. **(D)** The KEGG enrichment of the up-regulated DEGs in M4*vs.*M3. **(E)** The heat map of the enriched DEGs related to plant hormone signal transduction in M4*vs.*M3. **(F)** The heat map of the DEGs involved in galactose metabolism which was the significant enrichment pathway in M4*vs.*M3. **(G)** The heat map of the enriched DEGs associated with starch and sucrose metabolism in M4*vs.*M3.

There were 727 up-regulated DEGs exclusively identified in M3*vs.*M1 (Figure 2A). Based on KEGG metabolic pathway analysis, 12 DEGs were significantly enriched in plant hormone signal transduction pathway (Figure 2B; Supplementary Table 7). To comprehensively track the expression patterns, the FPKMs of these genes were investigated in each sample (Figure 2C). The results showed that three genes annotated with IAA signal transduction, Zardc15205(*ZaIAA11*), Zardc22092(*ZaIAA12*), and Zardc21658(*ZaGH3.1*), were up-regulated in male floral differentiation process, but three of *ZaSAUR* were significantly up-regulated in female floral differentiation process. Moreover, the Zardc46700(*ZaARR3*), a gene related to CTK, was detected significantly up-regulated during male floral differentiation and very low expression abundance was observed in female samples. The four ABA signal transduction pathway related genes were significantly up-regulated in female floral differentiation process, although they were increased in M4*vs.*M3. All of these results indicated that CTK may be involved in male floral differentiation, whereas ABA could be associated with female floral differentiation.

Furthermore, 2026 up-regulated DEGs were exclusively detected in M4*vs.*M3 (Figure 2A). Three important pathways were significantly enriched, including plant hormone signal transduction, galactose metabolism, and starch and sucrose metabolism pathways (Figure 2D). It was worth to note that most of the genes annotated with plant hormone signal transduction were up-regulated in male floral differentiation except Zardc28302(*ZaPYL4*) and Zardc09977(*ZaPYL5*), both of which were involved in ABA signal transduction (Figure 2E). Additionally, all of four genes annotated to galactose metabolism, Zardc31601(*ZaDIN10*), Zardc23456(*ZaRS5*), Zardc03128(*ZaSIP2*), and Zardc04821(*ZaSTS*), were highly significantly up-regulated in male floral differentiation (Figure 2F; Supplementary Table 8). The similar results were also obtained in 15 DEGs related to starch and sucrose metabolism pathways (Figure 2G). All of these results further revealed that CTK might play a crucial role in male floral differentiation, whereas ABA was associated with female floral differentiation. Meanwhile, the sugar metabolism may also provide some nutrient supplements for the male floral differentiation in *Z. armatum*.

### Regulatory patterns of female floral differentiation

**Figure 3:**
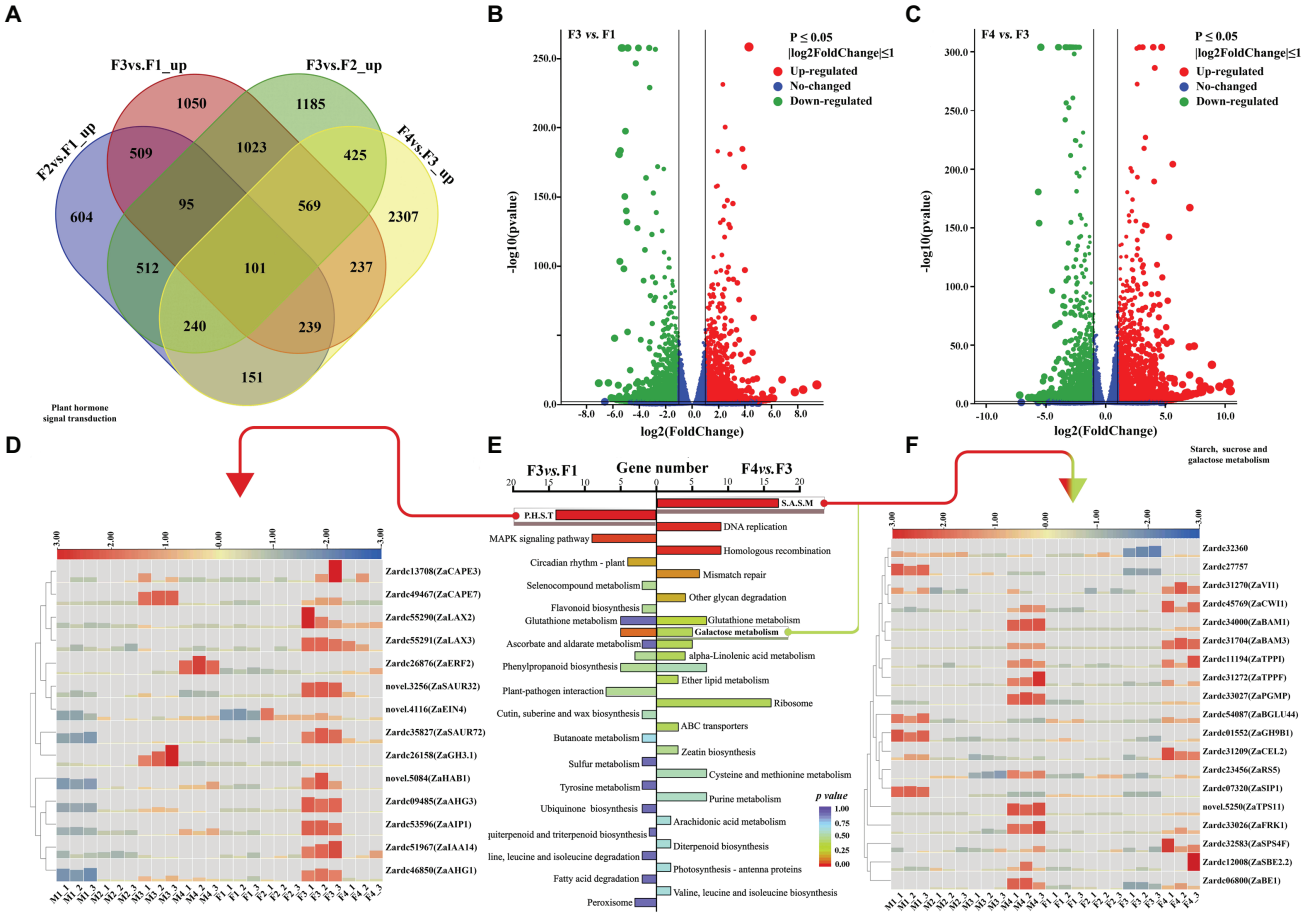
The DEGs and significant pathway analysis related to female floral differentiation. **(A)** The Venn diagram shared with the numbers of up-regulated DEGs in various comparisons of female floral samples. **(B)** The volcano plot of differential expressed genes (DEGs) in F3*vs.*F1. **(C)** The volcano plot of differential expressed genes (DEGs) in F4*vs.*F3. **(D)** The heat map of the DEGs associated with plant hormone signal transduction which was the significant enrichment pathway in F3*vs.*F1. **(E)** The enrichment pathways in F3*vs.*F1 and F4*vs.*F3. **(F)** The heat map of the DEGs related to starch, sucrose and galactose metabolism which was the significant enrichment pathway in F4*vs.*F3.

In totally, 690, 1723, 2,338, and 2,239 DEGs were up-regulated in F2*vs.*F1, F3*vs.*F1, F3*vs.*F2, and F4*vs.*F3 (Figure 3). Similar to the analyses in male floral differentiation, the F3*vs.*F1 and F4*vs.*F3 were used to identify the crucial genes and pathways involved in female floral differentiation.

There were 1722 and 2,106, 2,239 and 2030 DEGs isolated with noticeable up-regulation and down-regulation in F3*vs.*F1 and F4*vs.*F3, respectively (Figures 3B,C). According to KEGG enrichment for up-regulated DEGs in F3*vs.*F1, 14 DEGs were significantly enriched in plant hormone signal transduction pathway (Figure 3D; Supplementary Table 7). Similar results with M3*vs.*M1 showed that while a part of genes related to IAA signals were up-regulated in female floral differentiation, another was mainly up-regulated in male floral differentiation (Supplementary Table 8). However, the genes involved in ABA and SA were significantly up-regulated in female floral differentiation process. These results suggested that ABA and SA may be contribute to female floral differentiation, and IAA might be an essential member for floral sex differentiation.

Moreover, 22 up-regulated DEGs of F4*vs.*F3 were significantly enriched in sugar metabolism pathways, including 17 DEGs for starch and sucrose metabolism, and 5 DEGs for galactose metabolism (Figure 3E; Supplementary Table 7). Interestingly, the majority of these DEGs were obtained with notable up-regulation in male floral differentiation, despite the fact that their levels were gradually elevated in female floral differentiation (Figure 3F; Supplementary Table 8). Four genes, Zardc54087(*ZaBGLU44*), Zardc01552(*ZaGH9B1*), Zardc27757, and Zardc07320(*ZaSIP*) were up-regulated in M1 stage, and the six genes, Zardc06800(*ZaBE1*), Zardc31272(*ZaTPPF*), Zardc33026(*ZaFRK1*), Zardc33027(*ZaTPS11*), novel.5250(*ZaTPS11*), and Zardc34000(ZaBAM1) were observed to have high expression abundance in M4 stage. This information implied that the sugar substances are essential factors during floral differentiation in *Z. armatum*, however it might be more important for male floral differentiation.

### Identification the genes involved in floral sex differentiation in *Zanthoxylum armatum*

To comprehensively obtain the key genes involved in floral sex differentiation, the DEGs were identified among male and female samples. A total of 2,553, 2,995, 3,475, and 5,033 DEGs were isolated in the M1*vs.*F1, M2*vs.*F2, M3*vs.*F3, M4*vs.*F4, and 1,586, 2,251, 2,184 and 2,912 of which were, respectively, up-regulated in each comparison (Supplementary Figure 8). Surprisingly, all of the up-regulated DEGs were enriched in zeatin biosynthesis, starch and sucrose metabolism pathways in each comparison, especially in M3*vs.*F3 (Supplementary Figure 9).

**Figure 4:**
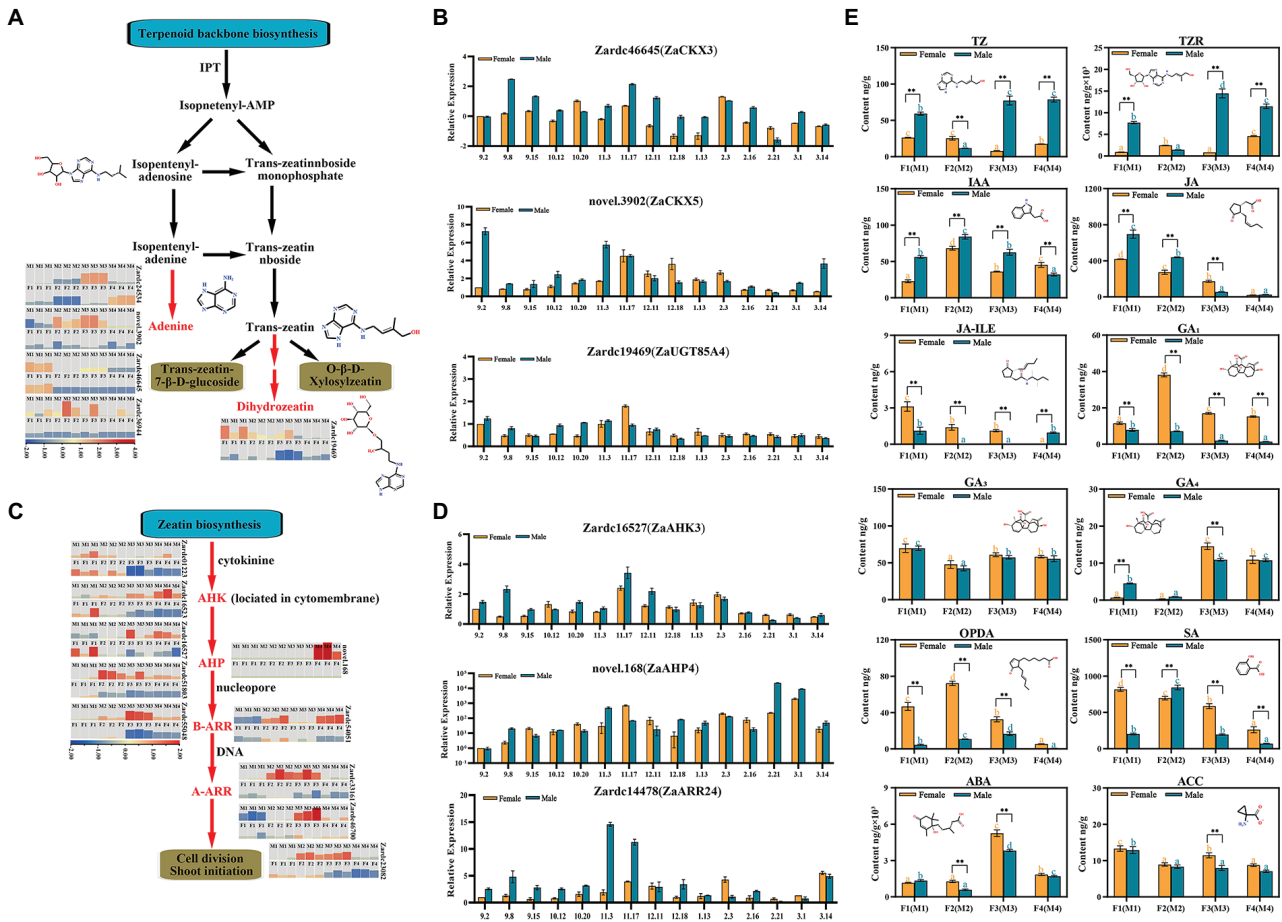
The identification of key genes related to cytokinin biosynthesis and signal transduction pathways enriched in male *vs.* female comparisons. **(A)** The biosynthesis cascade of zeatin biosynthesis pathway. Red fonts indicated the homologous differential expressed genes significantly up-regulated in this study. **(B)** The relative expression of the DEGs enriched in zeatin biosynthesis during 15 male and female floral samples. **(C)** The biosynthesis cascade of zeatin biosynthesis pathway. **(D)** The relative expression of the DEGs identified in cytokinin signal transduction pathways. **(E)** The investigation of plant hormones during floral sex differentiation process. * and **, respectively, means *p* < 0.05 and *p* < 0.01 between female and male samples. The different orange and blue letters indicated the significant difference (*p* < 0.05) among differentiation stages for male or female floral buds, respectively.

In total, six up-regulated DEGs were significantly enriched in zeatin biosynthesis process, and 3 of which were co-obtained in M2*vs.*F2, M3*vs.*F3 and M4*vs.*F4. All of these genes were subjected to BLASTX against the TAIR 11 protein database to obtained additional genetic annotations (Supplementary Table 7). The FPKM data present that the four genes associated with CKX subfamily, Zardc36944(*ZaCKX1*), Zardc46645(*ZaCKX3*), Zardc24534(*ZaCKX5*), and novel.3902(*ZaCKX6*), showed significant up-regulation in male floral differentiation process, especially in M2 and M3 stage (Figure 4A; Supplementary Table 8), whereas the lower expression was detected in female floral samples. Meanwhile, a gene, Zardc19469(*ZaUGT85A4*) which was attributed to UDP-glucosyl transferase superfamily in the dihydrozeatin-O-glucoside process, also displayed higher expression in male samples.

Additionally, 29 up-regulated DEGs were identified in plant hormone signal transduction pathways, including 10 of which were annotated with IAA, 12 with CTK, 1 with ethylene (ETH), 4 with SA, and 2 with jasmonic acid (JA; Supplementary Table 7; Supplementary Figure 10). Furthermore, six genes were annotated to *AHKs*, the cytokinin sensor located in cytomembrane, and mainly up-regulated in male floral differentiation, especially the Zardc23082 and Zardc55048(*ZaAHK4*). The similar results were that five DEGs involved in two-component signaling system, a kind of transcription factor named *Arabidopsis* response regulator (ARR), also showed very significant up-regulation in M2 and M3 stages of male floral differentiation. It was worth to note that novel.168(*ZaAHP4*), a new gene detected in present study which was a messenger to transfer the cytokinin signals into nucleus, was significantly up-regulated in M4 stage, but not detected in another floral differentiation periods, especially during the entire female floral differentiation process (Figure 4C; Supplementary Table 8). Additionally, the genes associated with JA and SA signal transduction were also screened in the up-regulated profiles of male floral differentiation (Supplementary Figure 10).

To further identify the genetic information of these key genes associated with cytokinin biosynthesis and signal transduction, male and female flower samples in 15 differentiation and maturation points from September to March of next year were selected to investigate their abundant expression (Figures 4B,D). The results showed that all of them had similar expression patterns with RNA-seq dataset during the floral sex differentiation, and most of which were obviously up-regulated in floral primordium periods between November and December, especially in male flowers. The above results indicated that cytokinin might be a vital factor for male floral differentiation in *Z. armatum*.

**Figure 5:**
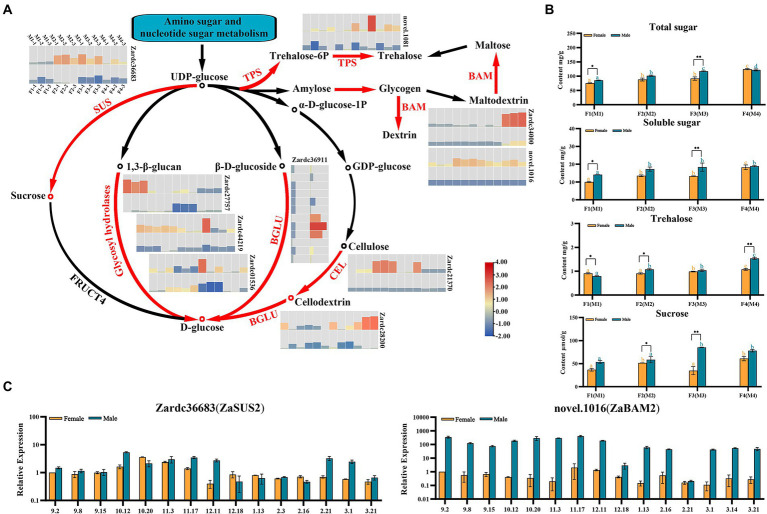
The identification of key genes related to sugar compounds biosynthesis pathways enriched in male *vs.* female comparisons. **(A)** The biosynthesis cascade of sucrose and starch biosynthesis pathway. Red fonts indicated the homologous differential expressed genes significantly up-regulated in this study. **(B)** The investigation of four sugar compounds during floral sex differentiation process. * and **, respectively, means *p* < 0.05 and *p* < 0.01 between female and male samples. The different orange and blue letters indicated the significant difference (p < 0.05) among differentiation stages for male or female floral buds, respectively. **(C)** The relative expression of the DEGs during 15 samples of male and female floral development and maturation process. The female sample at 2 September was used as control.

Based on the KEGG enrichment analysis, 15 up-regulated DEGs were detected in starch and sucrose metabolism pathway. The expression values of these DEGs in eight floral samples and their TAIR11 annotations are also listed in Supplementary Tables 7, 8. Interestingly, the major enriched pathways of sugar metabolism were the biosynthesis process of D-glucose and Trehalose (Figure 5). A total of seven enzymes involved in the biosynthesis steps were identified in our DEG profiles, which included sucrose synthase (SUS), beta-amylase (BAM), trehalose phosphate synthase (TPS), beta glucosidase (BGLU), cellulase (CEL), ADP-glucose pyrophosphorylase (APS), and glycosyl hydrolases (Figure 5A; Supplementary Table 7). Zardc36683(ZaSUS2), a gene encoded proteins catalyzed the initial formation of sucrose using UDP-glucose, was only significantly up-regulated in male floral samples. Similar results were obtained for another six DEGs which also used the UPD-glucose as a substance to catalyze the key reactions of D-glucose biosynthesis. Additionally, the other branch of these pathways was also significantly obtained and enriched in TPS biosynthesis. All of the genes in TPS pathways were significantly up-regulated in the entire male floral samples in this study.

Moreover, two genes were selected to confirm the genetic information of these key genes associated with sugar metabolism pathways using qRT-PCR detection (Figure 5B). The results showed that they were up-regulation in 15 male and female flower samples, especially the novel.1016(*ZaBAM2*) which was related to the maltose formation.

### Investigation of plant growth substances during male and female differentiation

To further confirm the roles of various plant hormones during floral sex differentiation process, a total of 12 phytohormone were examined in this study (Figure 4E). These phytohormones were observed to be very significantly different among male and female floral samples. Both of the trans-zeatin (TZ) and trans-zeatinriboside (TZR), the cytokinin compounds, were highly up-regulated in male floral differentiation, but very low concentration during female floral differentiation process, especially at M3*vs.*F3 stage. The IAA and JA were also significantly up-regulation in the earlier male floral differentiation stages, compared with female floral stages. Due to the important roles of gibberellins (GA) for floral formation, three dominant GA compounds were investigated during male and female floral differentiation in *Z. armatum*. Interestingly, GA_1_ was overwhelmingly in high concentration in female floral samples, whereas the very weak abundance was detected in male flowers. However, the GA_3_ and GA_4_ did not show any significantly different patterns between male and female floral samples. RNA-seq results revealed similar to GA, SA and ABA were also up-regulated in the female floral differentiation. Meanwhile, the other crucial chemical compounds associated with phytohormones were also analyzed in the present study, including 12-oxo-phytodienoic acid (OPDA) which is an important precursor in the biosynthesis of JA, jasmonic acid-isoleucine (JA-ILE) which is a complex of JA and isoleucine, and aminocyclopropanecarboxylic acid (ACC) which is an important precursor in the biosynthesis of ETH. The results showed that both of the OPDA and JA-ILE, the substances related to JA, were significantly high concentration in female floral differentiation, although they were gradually decreased during later floral differentiation and maturation process. Similar to GA_3_, ACC also did not exhibit a significant pattern during floral formation process in *Z. armatum*.

To further confirm the roles of sugars during the floral sex differentiation process, four sugar compounds were investigated in this study (Figure 5C). The total sugar, soluble sugar, trehalose and sucrose were showed the significant differences among the male and female floral samples, especially at M3 and F3 stages. These results suggested that the sugar compounds also play a crucial role for male floral differentiation in *Z. armatum*.

### DEGs analysis related to MADS-box transcription factors

**Figure 6:**
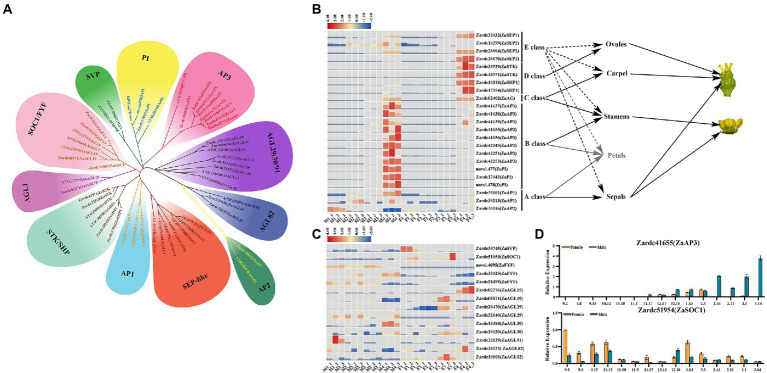
The identification of DEGs associated with MADS-box transcription factors. **(A)** The phylogenetic analysis of MADS-box DEGs screened in the male *vs.* female samples. **(B)** The heat map of DEGs related to ABCDE model. Due to the deletion of petals in *Z. armatum*, its related information was shown as gray color. **(C)** The heat map of DEGs involved in the transition from vegetative to reproductive growth. **(D)** The relative expression of two DEGs during 15 male and female floral samples.

Due to the vital regulatory roles and interaction with plant hormone transduction pathways, 37 DEGs related to MADS-box transcription factors (TFs) were isolated among the DEGs in four M*vs.*F comparisons (Supplementary Table 9). Based on the annotation with TAIR11 database and phylogenetic analyses, all of these DEGs could be divided into 11 subclades (Figure 6A), including *AP1*, *AP2*, *AP3/PI*, *STK/SHP*, *SEP*, *SVP*, *SOC1*, *FYF*, and various *AG-like* genes.

A total of 23 DEGs were identified to control the floral organ differentiation, and attributed to ABCDE model (Figure 6B). Two *ZaAP1* genes and one *ZaAP2* gene, Zardc39101, Zardc39213 and Zardc30916, were isolated that are mainly involved in male floral initiation and differentiation. It was worth to note that 11 DEGs of *AP3/PI-like* annotated with B class were unexpectedly obtained and exclusively up-regulated in male floral development process, which might indicate a reason why male flower could be triggered in *Z. armatum*. Only one gene, Zardc02402(*ZaAG*), was identified as a C class gene in the genomic profile of *Z. armatum*, which was exclusively detected at M4 and F4 stages and showed no significant differences among the male and female floral samples. Meanwhile, four DEGs related to D class, 2 of which were annotated with *ZaSTK* and remaining 2 that annotated with *ZaSHP1*, were specifically up-regulated in F4 stage, which suggested involvement of these genes in carpel maturation. Another four DEGs were associated with *SEP-like*, and most of them did not present obvious difference during floral sex differentiation process, except Zardc28170(*ZaSEP3*).

Moreover, 14 of DEGs were obtained to regulate the transition from vegetative to reproductive growth in this study (Figure 6C). Two DEGs were, respectively, annotated with the antagonistic function, Zardc51954(*ZaSOC1*) and Zardc30745(*ZaSVP*), they were mainly detected in female flower samples. Interestingly, three *ZaFYF* genes, the regulators of floral transition, were found significantly up-regulated in male floral samples, which might be involved in stamen differentiation and maturation. Furthermore, a total of nine DEGs were annotated with *AG-like* (*AGL*). Six of them were mainly detected in female floral samples, containing Zardc02276(*ZaAGL15*), Zardc08871(*ZaAGL19*), Zardc26470(*ZaAGL29*), Zardc19020(*ZaAGL30*), Zardc28223(*ZaAGL82*) and Zardc19106(*ZaAGL82*), and the rest of them showed relatively high expression in male floral initiation and differentiation process. To further confirm the genetic information of these MADS-box DEGs, the qRT-PCR was conducted to detect the expression patterns in 15 differentiation and maturation points during male and female floral differentiation process (Figure 6D). The results showed that all of them exhibited similar expression patterns with RNA-seq dataset during the floral sex differentiation, especially Zardc41655(*ZaAP3*) showed the extremely high expression in male samples.

### Co-expression network associated with floral sex differentiation in *Zanthoxylum armatum*

**Figure 7:**
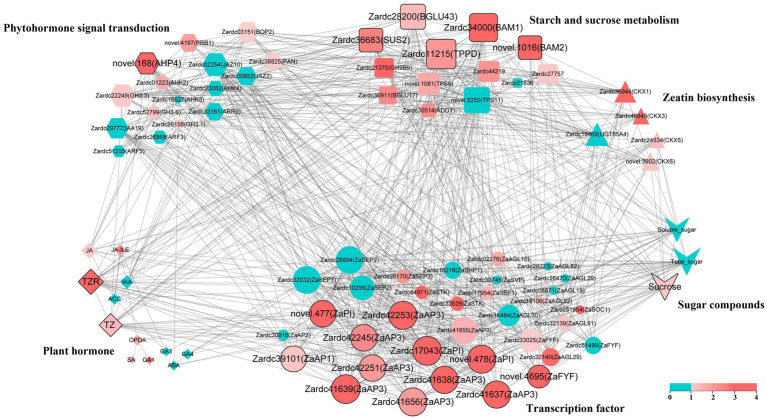
The co-expression network reveals the key genes involved in floral sex differentiation. The color of each bubble indicated the root mean square of log2(foldchange) among M3*vs.*F3 and M4*vs.*F4, and the size of bubble was the degree of each factor. The hub factors were highlighted with thick edging, which were identified by the top 40% of their degrees coupled with their color.

To further explore the crucial factors related to floral sex differentiation in *Z. armatum*, a total of 83 key factors based on their function and expression was chosen to construct the co-expression network in this study (Supplementary Table 10), including the plant hormone and sugar compounds. The DEGs selected from significant enrichment pathways among male and female samples, as well as the MADS TFs isolated from male and female DEG profiles. Pearson correlation was estimated using the data from 24 samples of these key factors. The crucial factors were identified by the top 40% of their degrees coupled with the root mean square of log2(foldchange) among M3*vs.*F3 and M4*vs.*F4. The *p* value was also set as a threshold to confirm the crucial factors in this study. Finally, the visualization in Cytoscape uncovered that 21 crucial factors were obtained in the co-expression network (Figure 7), which imply their core roles during floral sex differentiation process, especially male floral differentiation in *Z. armatum*.

Furthermore, TZ and TZR were isolated from the 12 plant hormone compounds (Figure 7), both of which belonged to CTK class. The compounds of JA class were also significantly high in abundance in the co-expression network. The majority of these components were likewise demonstrated to be considerably up-regulated, even if they were not all shown to the highest degree in the DEGs of zeatin biosynthesis. Meanwhile, a novel gene detected in the present RNA-seq dataset, novel.168(*ZaAHP4*) associated with CTK signal transduction, was filtered from the 16 phytohormone signal transduction pathways. The up-regulation of these factors in male samples suggested that the CTK, JA and *ZaAHP* may play important roles during male floral differentiation process. Additionally, the sugar substance was another crucial factor based on the co-expression network. Five DEGs, Zardc36683(*ZaSUS2*), Zardc28200(*ZaBGLU43*), Zardc11215(*ZaTPPD*), Zardc34000(*ZaBAM1*), and novel.1016(*ZaBAM2*), were identified in the subgroup of starch and sucrose metabolism. Moreover, sucrose was also selected in the sugar compounds. These results indicated the important functions of sucrose in male floral differentiation process. Because the vital roles of transcription factor, the DEGs regulated floral initiation and differentiation were also identified in the co-expression network. The results showed that a total of 12 TFs were obtained, and most of which were belonged to AP3/PI subfamily. Furthermore, other than the DEGs involved in ABCDE clusters, a new gene, novel.4695(*ZaFYF*), was also isolated and related to the transition from vegetative to reproductive growth.

### qRT-PCR validation

To experimentally verify the RNA-seq data, a total of 47 objects with 15 DEGs detected in M2*vs.*M1, M3*vs.*M2, M3*vs.*M1, M4*vs.*M3, F2*vs.*F1, F3*vs.*F2, F3*vs.*F1, M1*vs.*F1, M2*vs.*F2, M3*vs.*F3, M4*vs.*F4 were tested by qRT-PCR. These genes were selected because of their high foldchange and important function in floral sex differentiation process. The results for all of these genes were consistent and showed the similar trend of up- or down-regulation between the qRT-PCR and the RNA-seq platform (Supplementary Figure 11), and the correlation coefficient was 0.8027 among the two expression measurements (*R*^2^ = 0.8027). In summary, the results suggested that our transcriptome data accurately reflected the expression patterns of most genes in *Z. armatum*.

## Discussion

### The selective abortion of stamen or pistil primordia caused the floral sex differentiation in *Zanthoxylum armatum*

The formation of floral organs is an important product with the most varied structures in angiosperms during the long-term evolution. Previous studies have reported two different development mechanisms in floral sex formation among different plant species. In some species, the flowers were initially tended toward hermaphroditic differentiation, and the pistil or stamen primordia would be aborted during subsequent developmental process, resulting in the formation of unisexual flowers (Li S. et al., 2021; Li H. et al., 2021). However, the stamen and pistil proceeded independently without the degeneration of any floral organs in other unisexual species (Müller et al., 2020; Xue et al., 2020). In this study, the central position of male flower presents a slightly raised platform, which was a pistil position in female flower. Meanwhile, a slight space was also observed around the pistil, which would grow stamen in a male flower. Interestingly, we also observed the bisexual and unisexual flowers in the same tree or on the same branch, even in the same inflorescence during the previous field investigation. *Z. armatum* usually uses apomixis as the main reproduction way, but can produce male flowers and various floral types, which indicates its specific floral sex differentiation characteristics. Based on the observation of 26 floral samples using different methods, we suggested that it should belong to a pseudo-dioecious plants group in *Z. armatum*, which attributed the floral differentiation in *Z. armatum* to the first floral formation mechanism. Although three previous studies have reported that a few viruses might be related to the male flower differentiation (Cao et al., 2019, 2021; Xu et al., 2021), however, the underlying information of genetic response factors were not uncovered in *Z. armatum*. Additionally, a various type of bisexual flowers could also be specifically observed with different number of stamens or pistils in *Z. armatum*. Thus, our study suggested that the expression abundance of some endogenous factors may play more vital roles to regulate the floral sex differentiation in *Z. armatum*.

### Cytokinins might be associated with stamen differentiation in *Zanthoxylum armatum*

Plant hormones are suggested to be one of the important floral differentiation regulators in many plants and their roles on sex determination are extensively studied (Li S. et al., 2021; Li H. et al., 2021). In previous studies, cytokinins are shown to regulate the plant reproductive growth as the vital signal molecules (Ni et al., 2018). The contents of zeatin are reported to be higher in hermaphroditic flowers than male flowers of persimmon (Li S. et al., 2021; Li H. et al., 2021). Some publications also indicated that cytokinin response regulators (RR) act as a master regulator of sex determination for the entire Salicaceae family (Yang et al., 2020). Similarly, the male-specific *SyGI* annotated with ARR24 homologous gene could induce floral sex differentiation as a pistil inhibitory factor in kiwifruit (Akagi et al., 2018). It was reported that cytokinin signaling can interact with MADS-box proteins to regulate the inflorescence development (Han et al., 2014a,b). The *AG* could negatively regulates *WUS* expression to control the floral meristems activation, which was intermediated by *KNU*, encoding a C2H2-type zinc protein (Zúñiga-Mayo et al., 2019). Furthermore, *WUS* directly repressed the transcription of several A-type ARRs (Zhong and Kong, 2021), whereas type-B ARRs directly activated the expression of *WUS* and regulated carpel initiation by directly binding to transcriptional regulatory regions of *AG* in *Arabidopsis* (Rong et al., 2018). The recent research found that the expression of carpel identity genes, containing *AG*, *SHP* and *STK*, was significantly up-regulated and promoted gynoecial development in *arr24* homologous mutation of male kiwifruit (Varkonyi-Gasic et al., 2021). Meanwhile, the high expression levels of *AoARR* genes were detected in male flowers of garden asparagus (Li S. et al., 2021; Li H. et al., 2021). Despite the fact that the roles of ARRs varied among different species, all of these results can clarify that cytokinin is a crucial regulatory factor to control the floral sex differentiation in plants. Recently, Zhang et al. (2020) found that the cytokinin biosynthesis and signal transduction pathways were significantly up-regulation in male flower of *Z. armatum*. Similarly in the present study not only the genes of cytokinin biosynthesis and signal transduction pathways were significantly enriched and up-regulated in male floral samples, but also the content of zeatin and trans-zeatin showed very significantly high abundances in male floral differentiation stages than female flowers. These results may imply the cytokinin contributes to floral sex differentiation, especially in the stamen formation in *Z. armatum*. The further studies could attempt to validate their roles using the exogenous treatment to regulate the reproductive growth, and the genetic functions of *ARR* genes associated with male floral initiation could also be explored in *Z. armatum*.

### Carbohydrates might collaborate with cytokinin to regulate reproductive growth in *Zanthoxylum armatum*

It was usually present differential reproductive investment for male and female flowers in dioecious plants (Cai et al., 2021). Due to the directly roles associated with physiological processes, such as photosynthesis, translocation and respirations, the carbohydrates were one of the important factors for reproductive growth. In previous studies, the up-regulated DEGs were most enriched in starch and sucrose metabolism pathways in male floral buds of watermelon and persimmon (Li S. et al., 2021; Li H. et al., 2021). The down-regulation of starch and sucrose caused nutrients deficiency and disordered energy metabolism, which could lead to stamen abortion in *Solanum lycopersicum* and *Brassica campestris* (Wang J. et al., 2021; Yang et al., 2021). Similarly, the male trees of *Populus cathayana* had a higher capacity of carbon fixation and carbon sequestration (Wu et al., 2021). Furthermore, the SWEET transporter genes were significantly up-regulated during floral differentiation process, and could be capable to complement yeast cell uptake on sugar substrates (Wang P. et al. 2019). Similarly, in the present study, the starch and sucrose metabolism pathways were also significantly enriched in floral sex differentiation process in *Z. armatum*, especially in male floral samples.

Meanwhile, the elevated levels of nutrient can increase the rate of cytokinin biosynthesis (Kiba et al., 2011). Cytokinin biosynthesis genes (*IPT*, *CYP735A*) were significantly down-regulated under carbon starvation (Nardozza et al., 2020). In previous study, melatonin was found to synergistically interact with cytokinin biosynthesis and signaling pathways (Ma et al., 2018). The hydrolysis of sucrose can reduce pollen viability and germination, whereas the exogenous melatonin can regulate the carbohydrate balance to improve male fertility (Hu et al., 2020). At whole genome level, it has been reported that glucose could agonistically regulate about 70% of cytokinin regulatory genes (Kushwah and Laxmi, 2014). Based on the transcriptome sequencing analysis of various plants in recent years, both the cytokinin signaling and carbohydrate metabolism pathways were significantly enriched in floral sex differentiation process (Xin et al., 2019; Zhu et al., 2022), which was consistent with our results and also verified by the qRT-PCR, phytohormone and carbohydrate investigations in the present study. The DEGs after exogenous cytokinin treatment were also observed to be significantly involved in carbohydrate metabolism (Ni et al., 2018; Li et al., 2019). One of the reasons was that cytokinin response factors were involved in the regulation of intracellular chloroplast numbers, and exogenous cytokinins could induce the expression of sucrose transporters SUT and SWEET to affect energy metabolism pathways, a vital way to regulate the plant reproductive growth (McIntyre et al., 2021). In tomato, SiSWEET5b-mediated hexose transport was significantly required for pollen maturation (Ko et al., 2022). The present study also detected the prominently high abundances of sucrose content during male floral formations in *Z. armatum*. In summary, we suggested that the carbohydrates may play the crucial roles by collaborating with cytokinins in floral sex differentiation of *Z. armatum*, especially the male floral differentiation. However, there are few reports on whether there is a direct interaction between the genes associated with cytokinin and carbohydrate metabolism pathways. Therefore, exploring the crucial interaction among sugar compounds and cytokinin pathways using the various genetic methods was an important step in systematically revealing the genetic regulation mechanism involved in floral sex differentiation in *Z. armatum*.

### The floral differentiation could be identified as ABCDE model in *Zanthoxylum armatum*

Although the phytohormone and carbohydrates could induce the plant reproductive growth, the various regulatory signals could be directly or indirectly integrated into the genes associated with floral organ differentiation to control male and female floral formation. Cytokinin biosynthesis and signaling pathway could mediate the *AP1* function in the establishment of determinate floral meristems in *Arabidopsis* (Han et al., 2014a,b). The *AG* and *SHP* could be induced by cytokinin signaling *via* the type-B ARR proteins (Gómez-Felipe et al., 2021). Similarly, the hormone-mediated sugar pathways and high activity of MADS transcription factors could promote both the male and female floral initiation in *Metasequoia* (Zhao et al., 2015). In previous studies, the MADS genes have been well characterized in many plant species (Thapa et al., 2022), but their function has not been elucidated in *Z. armatum.* In this study, a total of 22 DEGs were annotated with the floral organ identity genes and belonged to ABDE class, respectively. Combined with the RNA-seq and qRT-PCR analysis, it was confirmed that *ZaAP3/PI* were significantly referred to male floral differentiation, and *ZaSTK/SHP* were specifically involved in female floral maturation, which positively coincided with the previous studies (Hsu et al., 2021; Varkonyi-Gasic et al., 2021). Recently, one publication reported that *ZbAGL11* could regulated the sporophytic apomixes by interacting with *ZbCYP450* and *ZbCAD11* proteins in *Z. bungeanum* (Red prickly ash; Fei et al., 2021). To further confirm the floral differentiation model in *Z. armatum* (Green prickly ash), the gene of C class was scanned from its genomic dataset. Interestingly, we obtained only one *AG* gene which was previously undetected in the significantly differential expression among male and female samples. One possible reason might be that many genes of B and D class already played their functions to regulate the stamen and pistil differentiation, and the more genetic information could be ascertained in the further studies. Thus, the current study clarifies that the floral organ regulation could be identified as ABCDE model in *Z. armatum*. Future studies are required to further investigate the alterations of floral meristem and organ identity genes in response to the ectopic expression of these MADS TFs, which would be benefited to explore more genetic information related to floral sex differentiation in *Z. armatum*.

**Figure 8:**
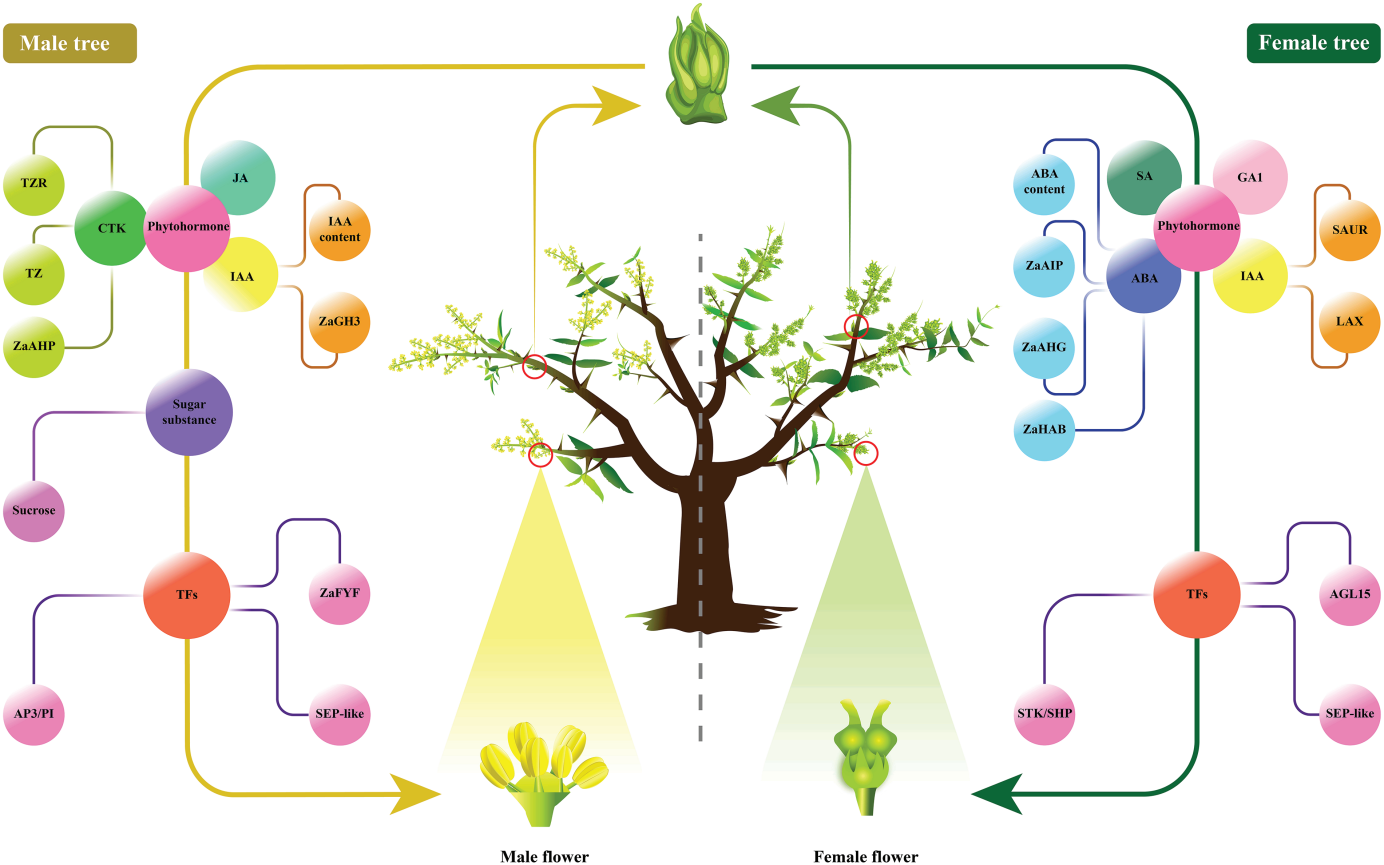
The schematic model illustrating the floral sex regulatory in *Z. armatum*. The male floral differentiation might be triggered by plant growth compounds and genes involved in phytohormone, sugar substance, and TFs, such as CTK, JA, IAA, sucrose, AP3/PI, ZaFYF, SEP-like. While, the female floral differentiation might be initiated by SA, GA1, ABA and IAA biosynthesis and signal transduction, as well as the transcription factors of STK/SHP, AGL15, SEP-like.

To sum up, our results comprehensively integrated the transcriptome, phytohormones and sugar compounds profiles coupled with the detailed morphological observations to identify the key factors involved in floral sex differentiation in Z. *armatum* (Figure 8). We hypothesized that the flower buds might be initially differentiated toward the same direction without gender differences in *Z. armatum*. However, a part of floral primordiums could form stamens under the induction of some plant growth substances, especially CTKs and sucrose signal molecules (Feng et al., 2020; Wang M. et al., 2021). The other flowers would continuously proceed to differentiate into female flowers without CTKs and sucrose stimulations. Moreover, these endogenous regulators associated with reproductive growth might be integrated into the ABCDE model to regulate the floral organ differentiation in *Z. armatum*. Nevertheless, our study only isolated the crucial pathways to link floral sex differentiation with multi-level endogenous regulatory factors in *Z. armatum*, especially to explore the important elements related to male floral formation. Further study is needed to investigate the abundances of the key factors in the various types of bisexual flowers with different number of stamens or pistils by HPLC-MS/MS and qRT-PCR, which could be used to validate and identify more important members. The whole genome sequencing (WGS) could be performed to search the sex-linked regions of chromosome, which would benefit to unfold more genetic information involved in floral sex determination in *Zanthoxylum*. Additionally, the exogenous phytohormone or sucrose treatments could be conduct to verify the floral sex alteration, it could also focus on generating the transgenic *Zanthoxylum* lines using *35*S-promoter over-expression and CRISPR/Cas9 constructs to verify the present results. The molecular techniques of promoter identification, yeast hybrid, BiFC and *in situ* hybridization could be carried out to clarify the genetic mechanism association with floral sex differentiation in forestry plants, which would contribute to developing the efficient floral sex regulation technologies and breeding high-yielding cultivars, so as to boost the yield of national and international productions and create more economic values.

## Data availability statement

The data presented in the study are deposited in the GEO repository, accession number GSE195749 and the data has been released in GEO database, and the GEO entries are accessible at: https://www.ncbi.nlm.nih.gov/geo/query/acc.cgi?acc=GSE195749.

## Author contributions

WH and WG formulated and designed the experiments. JF and XL collected the materials. JF, XL, JZ, and YX performed the experiments. WH drawn the figures, analyzed the data, and wrote the paper. JW, XZ, AW, YZ, TS, GC, and WG revised and proofread the paper. All authors contributed to the article and approved the submitted version.

## Funding

This work was supported by the Sichuan Science and Technology Fund Project (WH, 2020YJ0130), National Key Research and Development Program of China (WG, 2018YFD1000605, 2020YFD1000700), and the Forest and Bamboo Breeding Project of Sichuan Province for the Fifth Year Plan (W G, 2016NYZ0035, 2021YFYZ0032). The funding bodies had no role in the experimental design, sample collection, data analysis and interpretation, and manuscript writing.

## Conflict of interest

The authors declare that the research was conducted in the absence of any commercial or financial relationships that could be construed as a potential conflict of interest.

## Publisher’s note

All claims expressed in this article are solely those of the authors and do not necessarily represent those of their affiliated organizations, or those of the publisher, the editors and the reviewers. Any product that may be evaluated in this article, or claim that may be made by its manufacturer, is not guaranteed or endorsed by the publisher.

## Supplementary material

The Supplementary material for this article can be found online at: https://www.frontiersin.org/articles/10.3389/fpls.2022.976338/full#supplementary-material

## Footnotes

^1^https://doi.org/10.6084/m9.figshare.14400884.v1 (Accessed August 7, 2022).

^2^http://www.geneontology.org/ (Accessed August 7, 2022).

^3^https://www.kegg.jp/ (Accessed August 7, 2022).

^4^https://www.arabidopsis.org/ (Accessed August 7, 2022).
