# Supplementary material for: Integrated transcriptome and plant growth substance profiles to identify the regulatory factors involved in floral sex differentiation in Zanthoxylum armatum DC
Source: Front Plant Sci. 2022 Sep 2;13:976338. doi: 10.3389/fpls.2022.976338 (PMC9479546; doi:10.3389/fpls.2022.976338)
Supplement: Supplementary file 1 [file Data_Sheet_1.docx]

***Supplementary Figures***


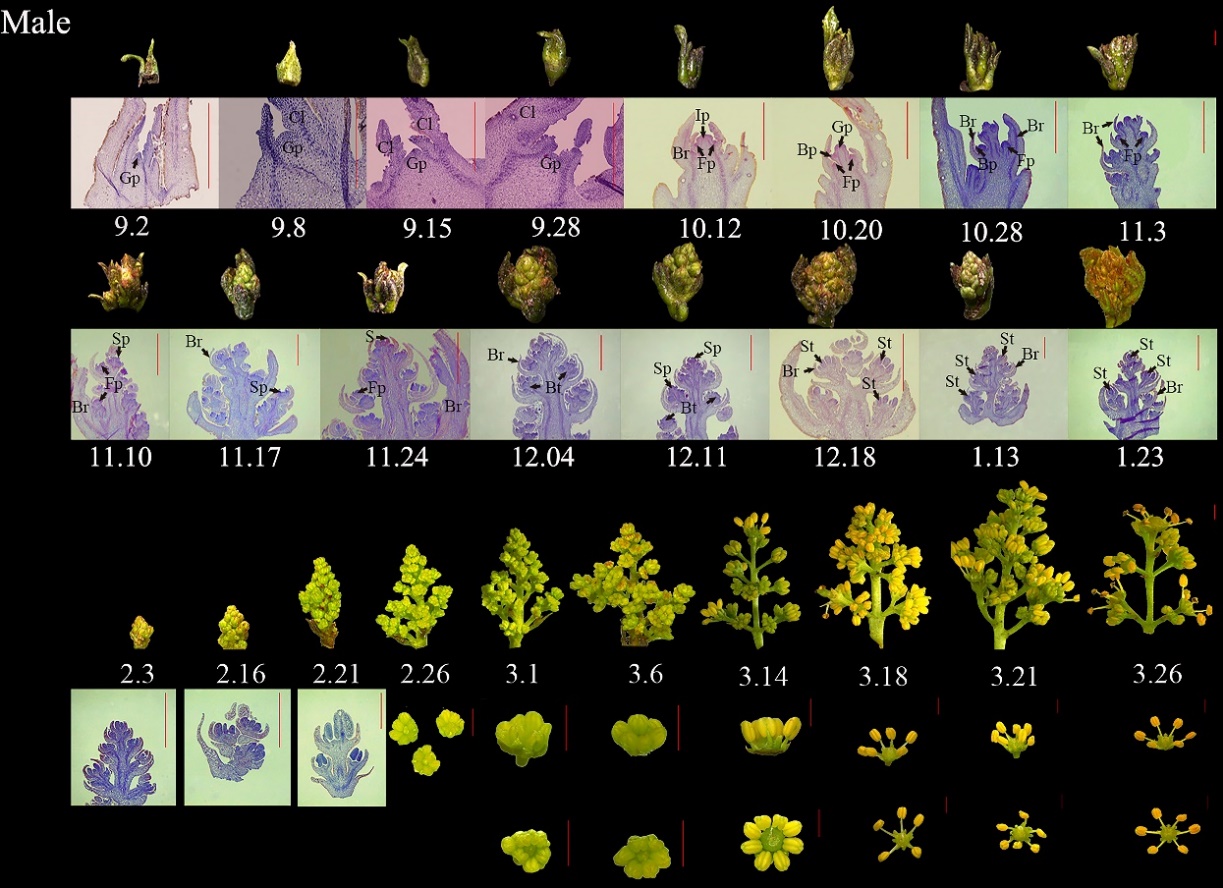


**Supplementary Figure 1 The observation of morphology and paraffin section during male floral differentiation process.**


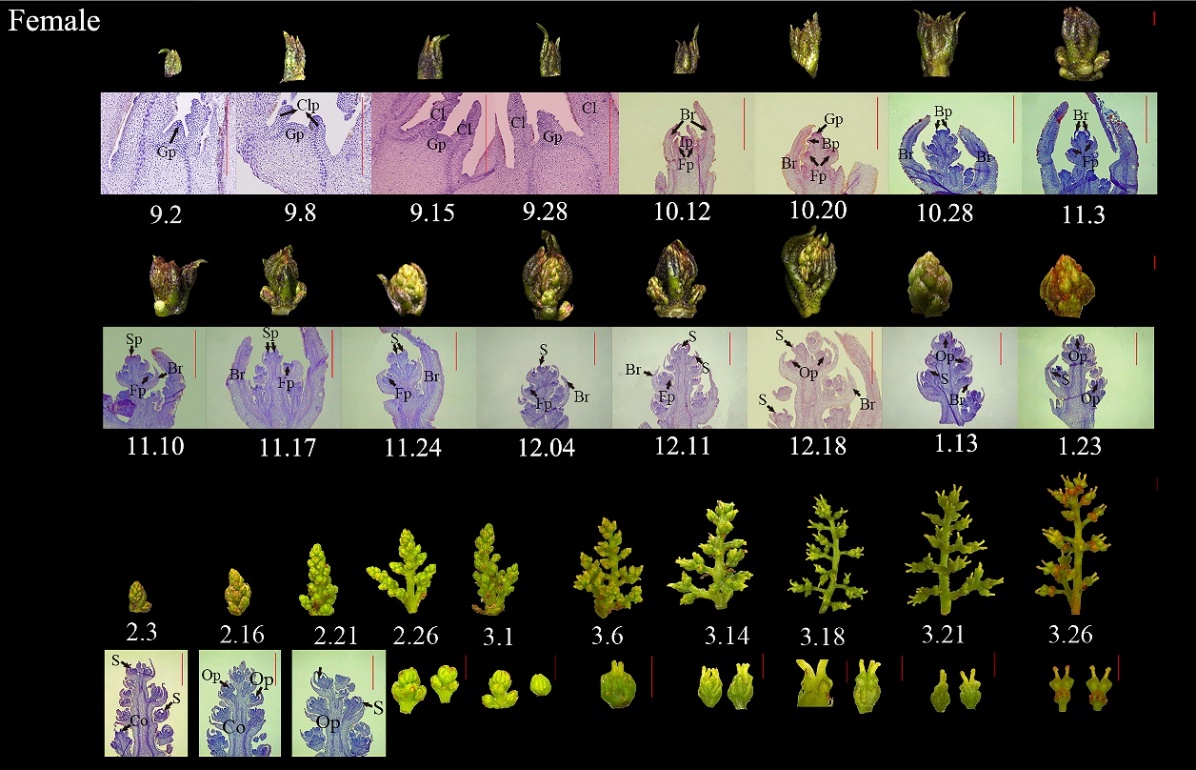


**Supplementary Figure 2 The observation of morphology and paraffin section in female floral differentiation process.**


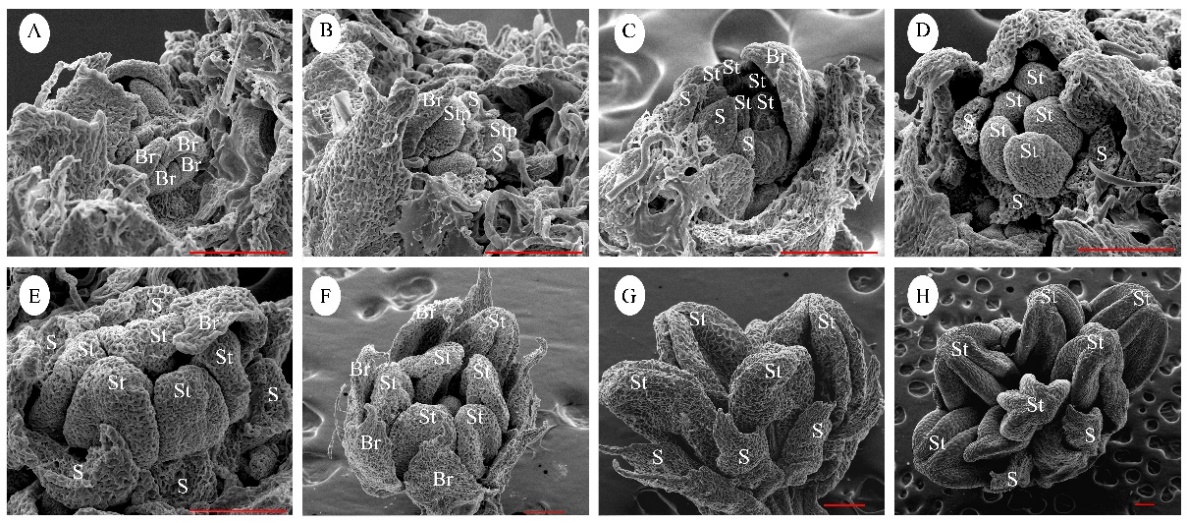


**Supplementary Figure 3 The SEM observation of eight male floral differentiation stage.** (A-H) The male flower samples respectively collected at 3 November, 4 December, and the next year 13 January, 3 February, 16 February, 21 February, 1 March, and 6 March.


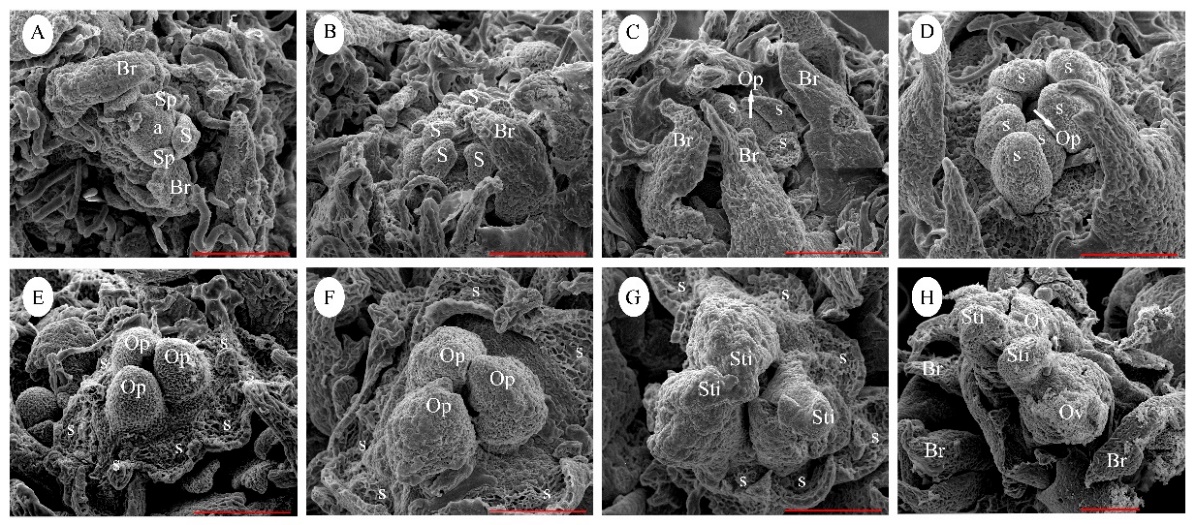


**Supplementary Figure 4 The SEM observation of eight female floral differentiation stage.** (A-H) The female flower samples respectively collected at the same date of male flowers.


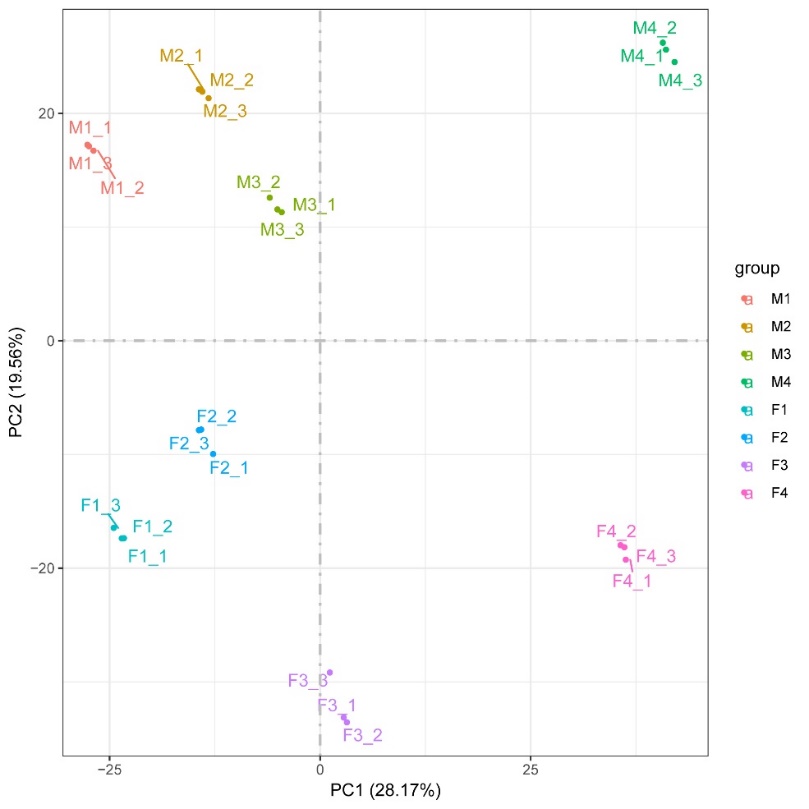


**Supplementary Figure 5 The principal component analysis (PCA) of each sample using RNA-seq in this study.**


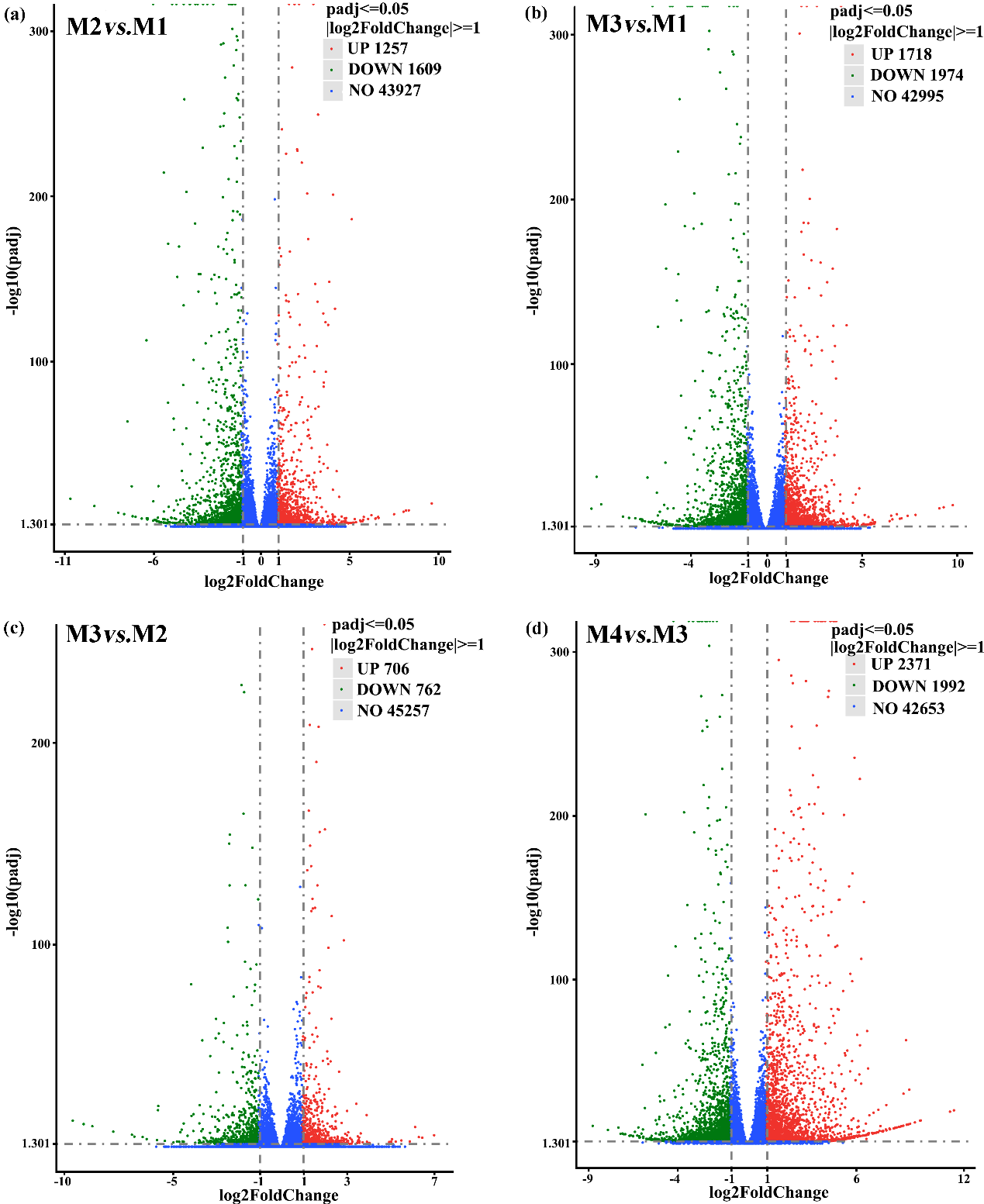


**Supplementary Figure 6 The volcano plot of DEGs in male floral differentiation process.** The abscissa shown the log2(fold change) of each DEG and the ordinate shown the significance of DEGs, *p*-adjust ≤ 0.05.


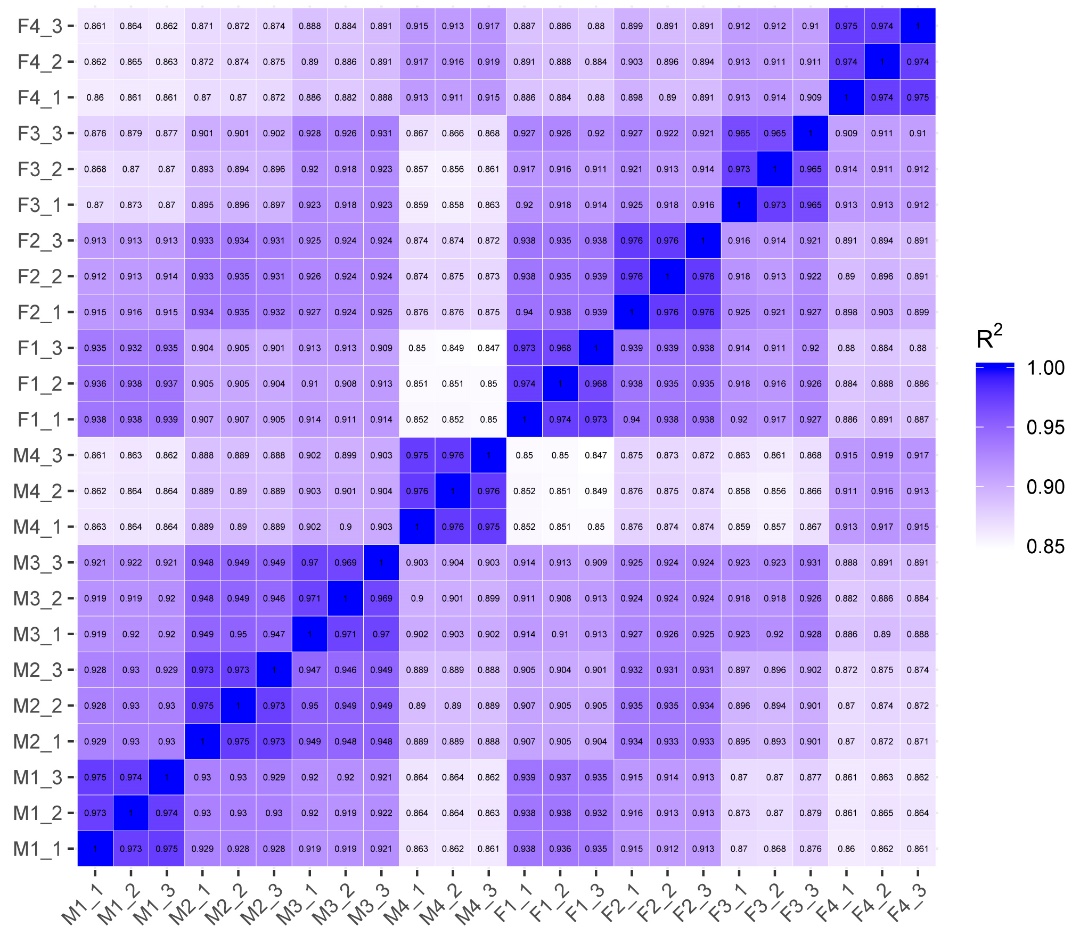


**Supplementary Figure 7 The correlation analysis of each sample using RNA-seq in this study.**


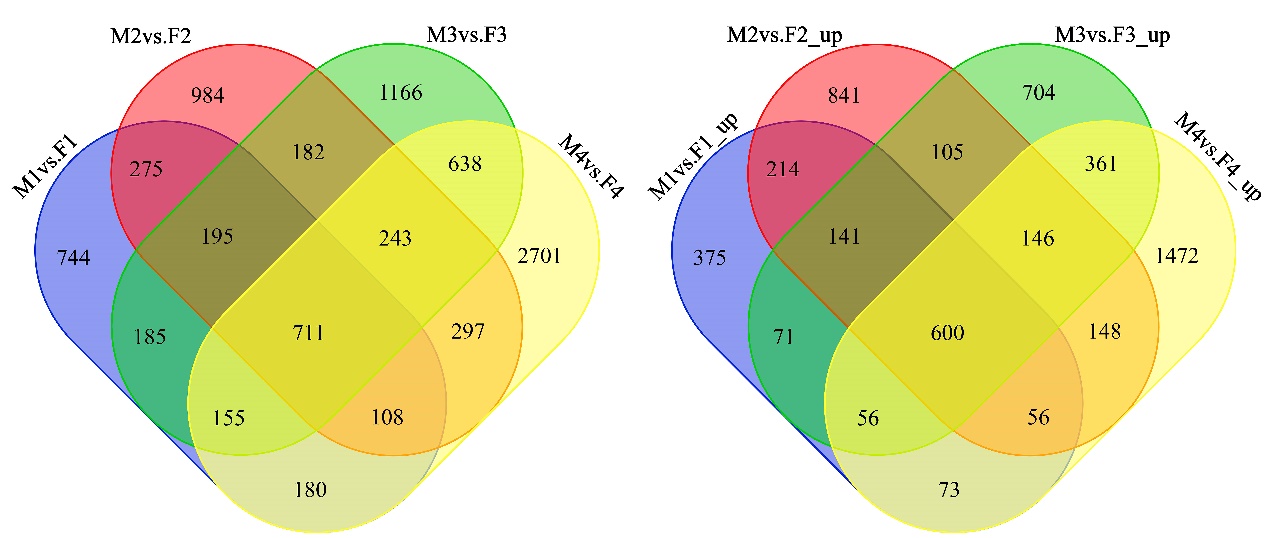


**Supplementary Figure 8 The venn diagram shared with the numbers of DEGs among male and female samples.** (a) The venn diagram shared with the numbers of all DEGs in various comparisons. (b) The venn diagram shared with the numbers of up-regulated DEGs in each comparison.

**
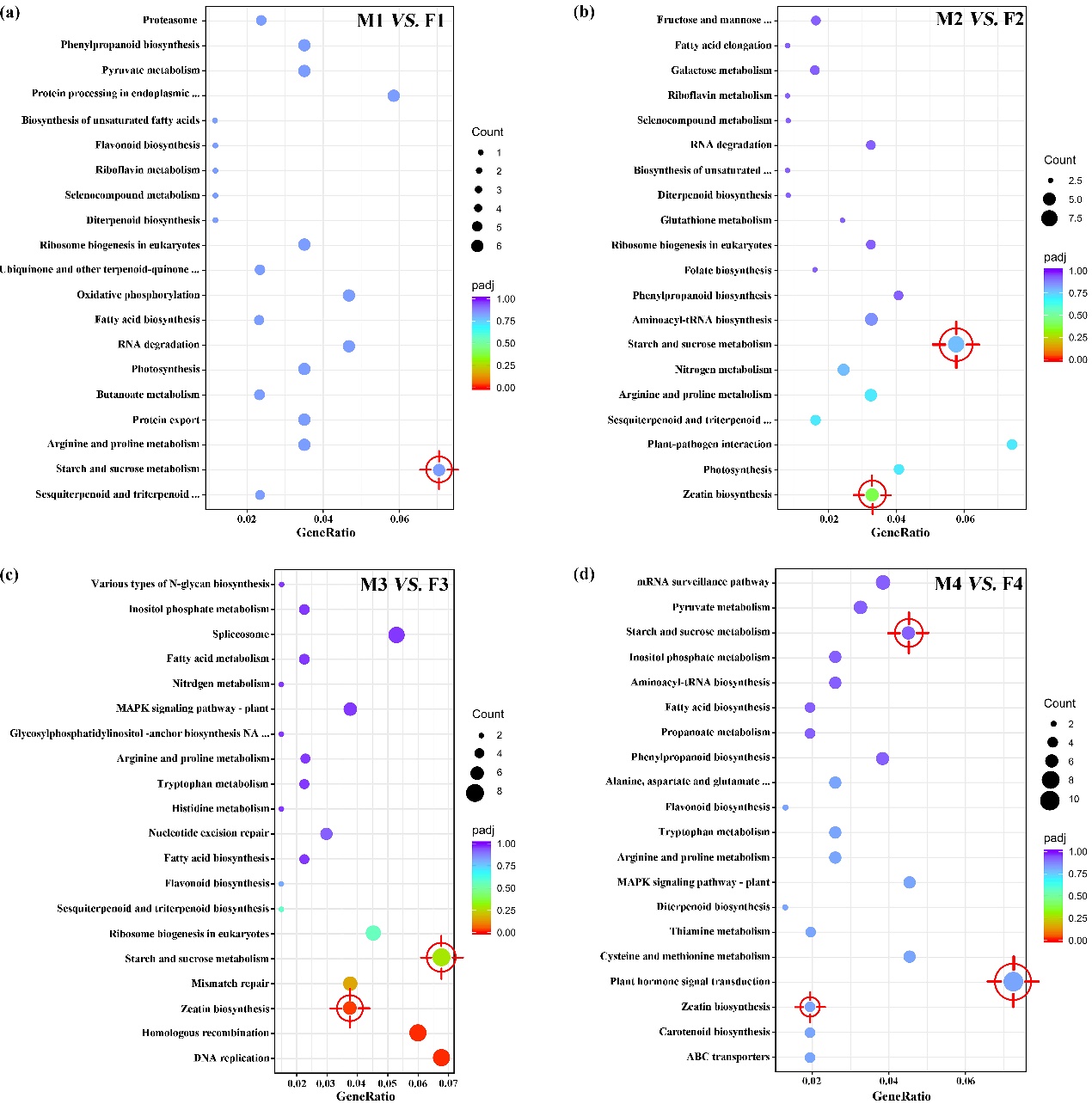
**

**Supplementary Figure 9 The KEGG enrichment of the up-regulated DEGs among male and female samples.**


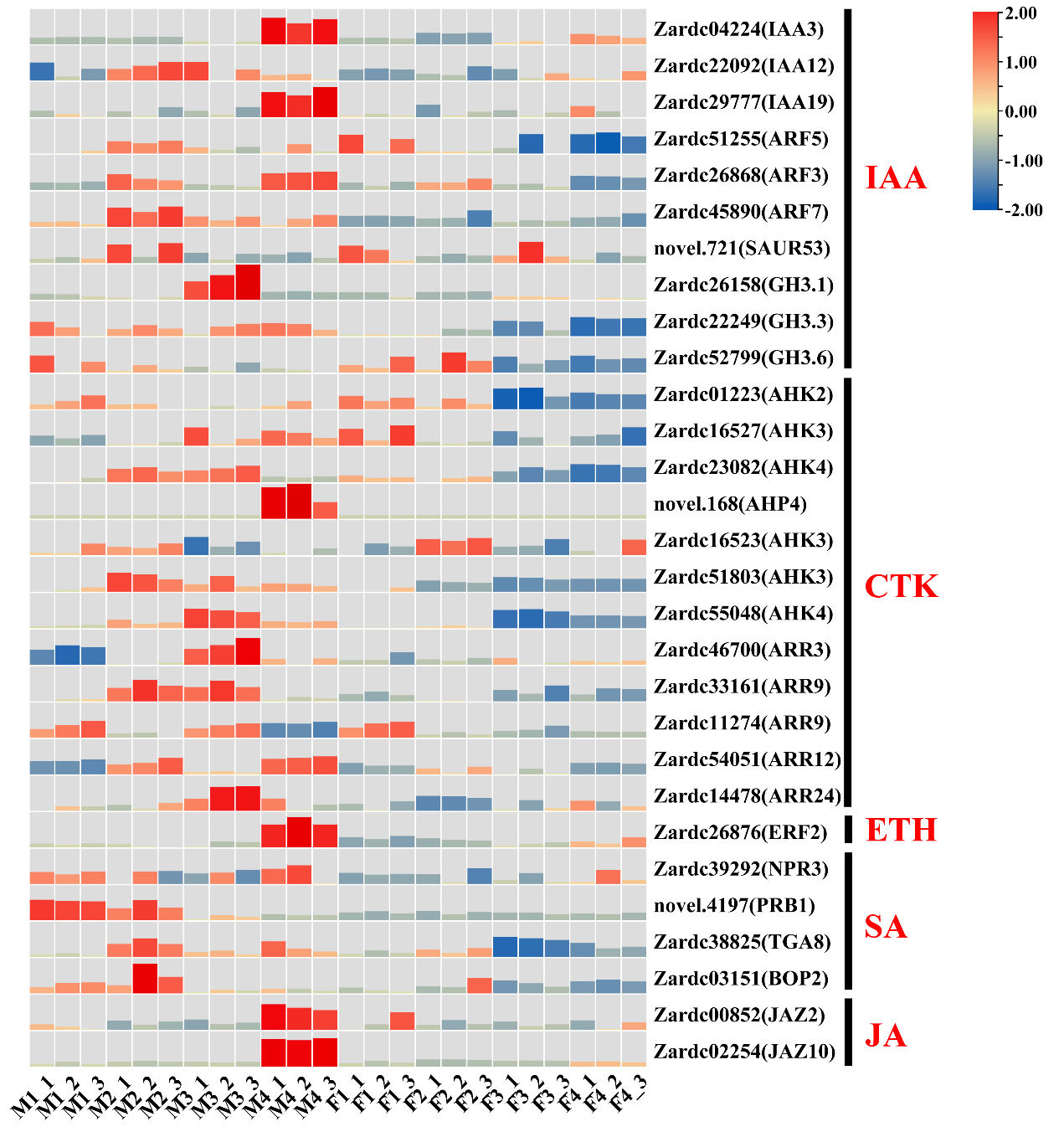
 **Supplementary Figure 10 The heat map of the DEGs associated with plant hormone signal transduction significant enriched in male vs. female samples.**


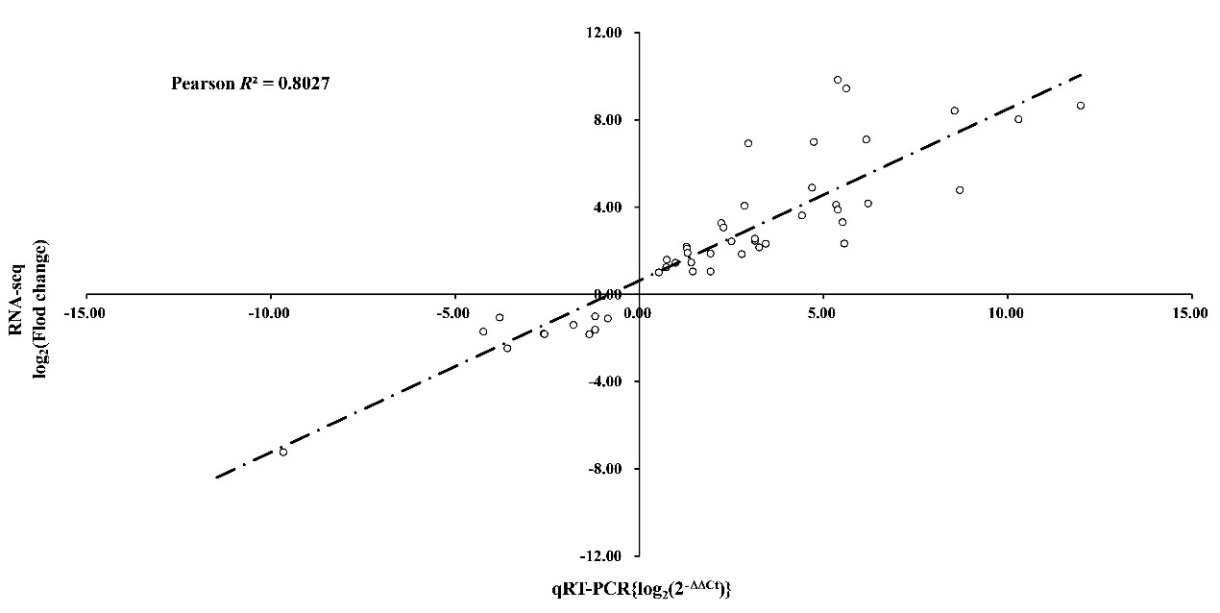


**Supplementary Figure 11 The correlation between qRT-PCR and RNA-seq data.**
