# Supplementary material for: Integrated transcriptome and plant growth substance profiles to identify the regulatory factors involved in floral sex differentiation in Zanthoxylum armatum DC
Source: Front Plant Sci. 2022 Sep 2;13:976338. doi: 10.3389/fpls.2022.976338 (PMC9479546; doi:10.3389/fpls.2022.976338)
Supplement: Supplementary file 2 [file Data_Sheet_2.docx]

*Supplementary tables*

**Supplementary Table 1 Regression equation of calibration curve for each phytohormone**

| **Compounds** | **Regression equation** | **R-square** | **Retention time / min** |
| --- | --- | --- | --- |
| ACC | y = 3827.86 x +63.0783 | 0.9934 | 0.72 |
| TZ | y = 10700.8 x + 57.8289 | 0.9991 | 5.31 |
| TZR | y = 2560.22 x + 449.907 | 0.9948 | 6.37 |
| GA1 | y =18.694 x +40.5036 | 0.9916 | 7.12 |
| GA3 | y = 5.81733 x+19.3679 | 0.9997 | 7.77 |
| SA | y = 484.246x + 244.266 | 0.9955 | 8.10 |
| ABA | y = 457.256 x +- 18.5114 | 0.9992 | 8.72 |
| JA | y = 271.476 x +-7.90756 | 0.9964 | 9.36 |
| JA-ILE | y =3880.21 x +5016.41 | 0.9927 | 10.37 |
| IAA | y = 17.7389 x + 1019.14 | 0.9916 | 10.48 |
| GA4 | y =44.721 x+-31.2617 | 0.9905 | 10.60 |
| OPDA | y = 8419.1 x +13365.7 | 0.9987 | 11.57 |

**Supplementary Table 2 The primers of qRT-PCR used for vaildation of expression trend in *Zanthoxylum armatum***

| **Gene ID** | **Forward primer** | **Reverse primer** |
| --- | --- | --- |
| novel.1016 | GGGATCGGCCAAACCCTA | GCCTGCTTTGCCCTTGTG |
| novel.168 | CTTGATGAGGGAATTCTGGACG | AATTTGTGAAGGGTCCTGTCC |
| novel.3902 | ATGGTGAAATGGATCAGAGTGC | TGGCTGGCTTGTACTGGGTC |
| Zardc08871 | TAGAACGCTATCAGAAGAGATCCAA | ACGGTTCAAGTCCACCTCCA |
| Zardc14478 | ATGAATCATCAGAAAATAACAGCACTTG | TCAACCGCTATGGTTGATCTTATGT |
| Zardc16527 | ACATTTACTGCTGTCTTCGGCA | GGCTCGGGTAGGTCTGGTG |
| Zardc17043 | AAGAAGTCAAGCAAGAAGTTGTGGG | CTGTTTGTTGCTAATGCCAGTGAG |
| Zardc19469 | ATCGTCTTCTCCGTAACACTCAC | GGCAAACCCGAGTCAGTCA |
| Zardc28170 | CGTTATTTGCGATGCTGAGGT | ACACATTTGGTTCTGGTGCTCC |
| Zardc28804 | ATCTTCTTGGAGAGGATTTGGGA | AGAATAAACTGCGTCTTGGTGGA |
| Zardc30745 | AAGTTGGACTGGGACGGGTG | TCAGTTGCTGTCTTAGTCGCTCA |
| Zardc33529 | TAGAGAATAGACTTGAACGAGGCAT | CTGAAACCTCTCCACTTCTGCTATC |
| Zardc36683 | TGGTATTCTTCGCAAGTGGATT | ATCGGGAACACCCTGTAACTC |
| Zardc41655 | ACATAAGCCCTTCAACAACGACTAA | TGGTTACATCCTCCAAATCTTCC |
| Zardc44971 | GGAGAATAGGCTTGAACGAGGC | TTGTCGGGGTGAGAGTAAGTAGTG |
| Zardc46645 | TCAGCCTCTAGCGATTACGG | TTTCAACTACTACTCCTCCTTCAGC |
| Zardc51954 | AGCCAACAGAGCAACACATACAGA | CCTTCTCCCAATAGTTTCTGCTTT |
| ZaUBQ | TCGAAGATGGCCGTACATTG | TCCTCTAAGCCTCAGCACCA |

**Supplementary Table 3 The quality of RNA extration in this study**

| **Sample name** | **Concentration (ng/ul)** | **Volume (ul)** | **Dose (ug)** | **RIN** | **Results** |
| --- | --- | --- | --- | --- | --- |
| M1 | 174 | 30 | 5.22 | 9.90 | qualified |
| M1 | 156 | 30 | 4.68 | 9.90 | qualified |
| M1 | 200 | 30 | 6 | 9.80 | qualified |
| M2 | 116 | 30 | 3.48 | 10.00 | qualified |
| M2 | 125 | 30 | 3.75 | 9.90 | qualified |
| M2 | 83 | 30 | 2.49 | 9.50 | qualified |
| M3 | 52 | 30 | 1.56 | 8.60 | qualified |
| M3 | 61 | 30 | 1.83 | 9.00 | qualified |
| M3 | 77 | 30 | 2.31 | 9.10 | qualified |
| M4 | 89 | 30 | 2.67 | 9.70 | qualified |
| M4 | 106 | 30 | 3.18 | 9.80 | qualified |
| M4 | 73 | 30 | 2.19 | 9.70 | qualified |
| F1 | 91 | 30 | 2.73 | 9.70 | qualified |
| F1 | 75 | 30 | 2.25 | 9.80 | qualified |
| F1 | 101 | 30 | 3.03 | 9.80 | qualified |
| F2 | 184 | 30 | 5.52 | 9.50 | qualified |
| F2 | 141 | 30 | 4.23 | 9.90 | qualified |
| F2 | 159 | 30 | 4.77 | 9.90 | qualified |
| F3 | 121 | 30 | 3.63 | 7.20 | qualified |
| F3 | 108 | 30 | 3.24 | 6.90 | qualified |
| F3 | 365 | 35 | 12.775 | 8.40 | qualified |
| F4 | 164 | 30 | 4.92 | 7.80 | qualified |
| F4 | 172 | 30 | 5.16 | 8.30 | qualified |
| F4 | 179 | 30 | 5.37 | 8.20 | qualified |

**Supplementary Table 4 Overview of the sequencing data in each sample**

| **Sample** | **Raw reads** | **Clean reads** | **Clean bases** | **Error rate** | **Q20** | **Q30** | **GC content / %** |
| --- | --- | --- | --- | --- | --- | --- | --- |
| M1_1 | 46132546 | 44124138 | 6.62G | 0.03 | 97.30 | 92.59 | 44.26 |
| M1_2 | 45051826 | 42921042 | 6.44G | 0.03 | 97.25 | 92.53 | 44.29 |
| M1_3 | 47209342 | 44811354 | 6.72G | 0.03 | 97.39 | 92.78 | 44.15 |
| M2_1 | 44297318 | 42458868 | 6.37G | 0.03 | 97.24 | 92.47 | 43.85 |
| M2_2 | 43689422 | 42073306 | 6.31G | 0.03 | 97.28 | 92.48 | 43.91 |
| M2_3 | 44048880 | 41667864 | 6.25G | 0.03 | 97.26 | 92.51 | 43.85 |
| M3_1 | 41955988 | 40777940 | 6.12G | 0.03 | 97.05 | 92.03 | 44.27 |
| M3_2 | 44288980 | 42712106 | 6.41G | 0.03 | 97.26 | 92.54 | 44.37 |
| M3_3 | 44716346 | 42440238 | 6.37G | 0.03 | 97.33 | 92.68 | 44.31 |
| M4_1 | 45065810 | 43174684 | 6.48G | 0.03 | 97.25 | 92.45 | 43.88 |
| M4_2 | 46191962 | 44149902 | 6.62G | 0.03 | 97.25 | 92.48 | 43.92 |
| M4_3 | 47167824 | 44888964 | 6.73G | 0.03 | 97.12 | 92.17 | 43.94 |
| F1_1 | 45579774 | 44092766 | 6.61G | 0.03 | 96.66 | 91.22 | 44.16 |
| F1_2 | 48534652 | 46357784 | 6.95G | 0.03 | 97.18 | 92.35 | 44.07 |
| F1_3 | 47565092 | 46176092 | 6.93G | 0.03 | 97.14 | 92.28 | 44.14 |
| F2_1 | 48171372 | 46637908 | 7.00G | 0.03 | 97.22 | 92.43 | 44.01 |
| F2_2 | 41294252 | 40103222 | 6.02G | 0.03 | 97.09 | 92.10 | 43.97 |
| F2_3 | 44537252 | 42949458 | 6.44G | 0.03 | 97.18 | 92.34 | 43.95 |
| F3_1 | 41793822 | 40876874 | 6.13G | 0.03 | 97.06 | 92.10 | 44.27 |
| F3_2 | 46608872 | 45029366 | 6.75G | 0.03 | 97.33 | 92.67 | 44.13 |
| F3_3 | 44458798 | 42976480 | 6.45G | 0.03 | 97.66 | 93.39 | 44.50 |
| F4_1 | 45974150 | 44286544 | 6.64G | 0.03 | 97.71 | 93.39 | 43.99 |
| F4_2 | 45760934 | 44295806 | 6.64G | 0.03 | 97.72 | 93.39 | 44.11 |
| F4_3 | 43467428 | 42095668 | 6.31G | 0.03 | 97.76 | 93.57 | 43.96 |

**Supplementary Table 5 The statistics of RNA-seq data mapping to the genome profile in this study**

| **Sample** | **Total reads** | **Total map** | **Unique map** | **Multi map** | **Splice map** | **Unsplice map** | **Proper map** |
| --- | --- | --- | --- | --- | --- | --- | --- |
| M1_1 | 44124138 | 36970508(83.79%) | 32381813(73.39%) | 4588695(10.4%) | 13259404(30.05%) | 19122409(43.34%) | 30158470(68.35%) |
| M1_2 | 42921042 | 35933519(83.72%) | 31567636(73.55%) | 4365883(10.17%) | 12882509(30.01%) | 18685127(43.53%) | 29312152(68.29%) |
| M1_3 | 44811354 | 37800885(84.36%) | 33138488(73.95%) | 4662397(10.4%) | 13588114(30.32%) | 19550374(43.63%) | 30818766(68.77%) |
| M2_1 | 42458868 | 36056916(84.92%) | 31772751(74.83%) | 4284165(10.09%) | 12986079(30.59%) | 18786672(44.25%) | 29383998(69.21%) |
| M2_2 | 42073306 | 35737189(84.94%) | 31504014(74.88%) | 4233175(10.06%) | 12892502(30.64%) | 18611512(44.24%) | 29023638(68.98%) |
| M2_3 | 41667864 | 35134576(84.32%) | 30823882(73.98%) | 4310694(10.35%) | 12528049(30.07%) | 18295833(43.91%) | 28608812(68.66%) |
| M3_1 | 40777940 | 32830255(80.51%) | 29070556(71.29%) | 3759699(9.22%) | 11832270(29.02%) | 17238286(42.27%) | 26446360(64.85%) |
| M3_2 | 42712106 | 34151822(79.96%) | 29980725(70.19%) | 4171097(9.77%) | 12212364(28.59%) | 17768361(41.6%) | 27657272(64.75%) |
| M3_3 | 42440238 | 34056385(80.25%) | 29878438(70.4%) | 4177947(9.84%) | 12103022(28.52%) | 17775416(41.88%) | 27607350(65.05%) |
| M4_1 | 43174684 | 36290911(84.06%) | 32174044(74.52%) | 4116867(9.54%) | 13220618(30.62%) | 18953426(43.9%) | 29649568(68.67%) |
| M4_2 | 44149902 | 36960643(83.72%) | 32670104(74.0%) | 4290539(9.72%) | 13367219(30.28%) | 19302885(43.72%) | 30093618(68.16%) |
| M4_3 | 44888964 | 37277126(83.04%) | 33000332(73.52%) | 4276794(9.53%) | 13449774(29.96%) | 19550558(43.55%) | 30288644(67.47%) |
| F1_1 | 44092766 | 35773352(81.13%) | 31425464(71.27%) | 4347888(9.86%) | 12786756(29.0%) | 18638708(42.27%) | 28767658(65.24%) |
| F1_2 | 46357784 | 38193043(82.39%) | 33432061(72.12%) | 4760982(10.27%) | 13484691(29.09%) | 19947370(43.03%) | 30855302(66.56%) |
| F1_3 | 46176092 | 38303732(82.95%) | 33603317(72.77%) | 4700415(10.18%) | 13740067(29.76%) | 19863250(43.02%) | 30715950(66.52%) |
| F2_1 | 46637908 | 39755442(85.24%) | 35186762(75.45%) | 4568680(9.8%) | 14588381(31.28%) | 20598381(44.17%) | 32044040(68.71%) |
| F2_2 | 40103222 | 34305764(85.54%) | 30293761(75.54%) | 4012003(10.0%) | 12594564(31.41%) | 17699197(44.13%) | 27595278(68.81%) |
| F2_3 | 42949458 | 36725946(85.51%) | 32349046(75.32%) | 4376900(10.19%) | 13469353(31.36%) | 18879693(43.96%) | 29728812(69.22%) |
| F3_1 | 40876874 | 32276224(78.96%) | 28496146(69.71%) | 3780078(9.25%) | 11336759(27.73%) | 17159387(41.98%) | 25708118(62.89%) |
| F3_2 | 45029366 | 35579672(79.01%) | 31273038(69.45%) | 4306634(9.56%) | 12426521(27.6%) | 18846517(41.85%) | 28485158(63.26%) |
| F3_3 | 42976480 | 33718627(78.46%) | 29610981(68.9%) | 4107646(9.56%) | 11898763(27.69%) | 17712218(41.21%) | 27182552(63.25%) |
| F4_1 | 44286544 | 37078704(83.72%) | 32855302(74.19%) | 4223402(9.54%) | 13362344(30.17%) | 19492958(44.02%) | 30268960(68.35%) |
| F4_2 | 44295806 | 37040546(83.62%) | 32863404(74.19%) | 4177142(9.43%) | 13386734(30.22%) | 19476670(43.97%) | 29949854(67.61%) |
| F4_3 | 42095668 | 35154646(83.51%) | 31168641(74.04%) | 3986005(9.47%) | 12641251(30.03%) | 18527390(44.01%) | 28825204(68.48%) |

**Supplementary Table 6 The statistics of the gene structure information**

| **Sample** | **Exon** | **Intron** | **Intergenic** |
| --- | --- | --- | --- |
| M1_1 | 4420499937(80.1466%) | 316536021(5.739%) | 778479008(14.1143%) |
| M1_2 | 4290846437(80.0611%) | 303577861(5.6643%) | 765043567(14.2746%) |
| M1_3 | 4504591822(79.8819%) | 326129605(5.7834%) | 808339962(14.3347%) |
| M2_1 | 4229692156(78.6457%) | 350725973(6.5213%) | 797743405(14.833%) |
| M2_2 | 4196103050(78.713%) | 349905657(6.5637%) | 784881611(14.7233%) |
| M2_3 | 4086790025(78.0003%) | 344272653(6.5708%) | 808388379(15.4289%) |
| M3_1 | 3843570720(78.4961%) | 321468111(6.5652%) | 731472684(14.9386%) |
| M3_2 | 3983687109(78.1987%) | 344040466(6.7534%) | 766588449(15.0479%) |
| M3_3 | 3951266980(77.8156%) | 328287809(6.4652%) | 798175668(15.7191%) |
| M4_1 | 4226111910(78.0759%) | 366668859(6.7741%) | 820046352(15.1501%) |
| M4_2 | 4283536722(77.7076%) | 386272259(7.0074%) | 842572449(15.2851%) |
| M4_3 | 4306590114(77.4697%) | 381096398(6.8554%) | 871373843(15.6748%) |
| F1_1 | 4257807713(79.8043%) | 316211418(5.9268%) | 761293152(14.269%) |
| F1_2 | 4493009133(78.9002%) | 345377241(6.065%) | 856162776(15.0348%) |
| F1_3 | 4563289368(79.8556%) | 362399422(6.3418%) | 788734417(13.8025%) |
| F2_1 | 4711101110(79.4521%) | 355206615(5.9905%) | 863179547(14.5574%) |
| F2_2 | 4057261336(79.2884%) | 323951590(6.3308%) | 735877188(14.3808%) |
| F2_3 | 4343160265(79.2868%) | 346660570(6.3285%) | 787961784(14.3847%) |
| F3_1 | 3698180162(76.8588%) | 300089194(6.2367%) | 813387948(16.9045%) |
| F3_2 | 4030912246(76.0036%) | 335853544(6.3326%) | 936816676(17.6638%) |
| F3_3 | 3920840883(77.9943%) | 306843552(6.1038%) | 799400427(15.9019%) |
| F4_1 | 4267874653(77.186%) | 348842438(6.3089%) | 912617288(16.505%) |
| F4_2 | 4293089013(77.7209%) | 347629227(6.2934%) | 883009611(15.9858%) |
| F4_3 | 4059632729(77.4424%) | 326991913(6.2378%) | 855509868(16.3199%) |

**Supplementary Table 7 The TAIR annotation of the genes selected from the KEGG enrichment**

| **Comparison** | **Pathways** | **Genes** | ***At.* ID** | ***At.* name** | **Blastx to TAIR10 database** |
| --- | --- | --- | --- | --- | --- |
| **M3*vs.*M1** | **Plant hormone  signal transduction** | Zardc49467 | AT2G14580 | AtPRB1 | response to ethylene,response to jasmonic acid, response to salicylic acid |
|  |  | Zardc09485 | AT3G11410 | AtAHG3 | abscisic acid-activated signaling pathway |
|  |  | Zardc46850 | AT5G51760 | AtAHG1 | response to abscisic acid |
|  |  | Zardc53596 | AT1G07430 | AtAIP1 | negative regulation of abscisic acid-activated signaling pathway |
|  |  | Zardc46700 | AT1G59940 | AtARR3 | cytokinin-activated signaling pathway, phosphorelay signal transduction system |
|  |  | novel.5084 | AT1G72770 | AtHAB1 | abscisic acid-activated signaling pathway |
|  |  | Zardc35827 | AT3G12830 | AtSAUR72 | meristem development, response to salicylic acid |
|  |  | Zardc26158 | AT2G14960 | AtGH3.1 | acts upstream of or within |
|  |  | Zardc15205 | AT4G28640 | AtIAA11 | response to auxin,regulation of transcription, DNA-templated |
|  |  | novel.3256 | AT2G46690 | AtSAUR32 | alpha-amino acid metabolic process, carboxylic acid biosynthetic process |
|  |  | Zardc22092 | AT1G04550 | AtBDL | response to auxin, root development, xylem and phloem pattern formation |
|  |  | novel.1294 | AT1G75590 | AtSAUR52 | response to auxin,developmental growth, plant epidermis development |
|  |  |  |  |  |  |
| **M4*vs.*M3** | **Starch and sucrose  metabolism** | Zardc31704 | AT4G17090 | AtBAM3 | maltose biosynthetic process, response to cold, starch catabolic process |
|  |  | novel.5250 | AT2G18700 | AtTPS11 | trehalose biosynthetic process, trehalose metabolism in response to stress |
|  |  | Zardc33027 | AT5G51820 | AtPGMP | carbohydrate metabolic process, starch biosynthetic process |
|  |  | Zardc34000 | AT3G23920 | AtBAM1 | starch catabolic process |
|  |  | Zardc48948 | AT4G39210 | AtAPL3 | glycogen biosynthetic process, starch biosynthetic process |
|  |  | Zardc33026 | AT5G51830 | AtFRK1 | starch biosynthetic process, fructose metabolic process |
|  |  | Zardc08022 | AT2G18700 | AtTPS11 | trehalose biosynthetic process, trehalose metabolism in response to stress |
|  |  | Zardc31272 | AT4G12430 | AtTPPF | trehalose biosynthetic process |
|  |  | Zardc11215 | AT1G35910 | AtTPPD | trehalose biosynthetic process |
|  |  | Zardc10599 | AT1G23870 | AtTPS9 | trehalose biosynthetic process, trehalose metabolism in response to stress |
|  |  | Zardc12008 | AT5G03650 | AtSBE2.2 | carbohydrate metabolic process, glycogen biosynthetic process |
|  |  | Zardc45769 | AT3G13790 | AtCWI1 | carbohydrate metabolic process |
|  |  | Zardc11194 | AT5G10100 | AtTPPI | trehalose biosynthetic process |
|  |  | Zardc08999 | AT1G76130 | AtAMY2 | extracellular region, response to inorganic substance, response to radiation |
|  |  | Zardc02022 | AT2G45880 | AtBAM7 | regulation of shoot system development |
|  |  |  |  |  |  |
|  | **Galactose metabolism** | Zardc03128 | AT3G57520 | AtSIP2 | raffinose catabolic process, response to oxidative stress |
|  |  | Zardc33027 | AT5G51820 | AtPGMP | carbohydrate metabolic process, starch biosynthetic process |
|  |  | Zardc04821 | AT4G01970 | AtSTS | response to oxidative stress |
|  |  | Zardc23456 | AT5G40390 | AtRS5 | mannitol biosynthetic process, sucrose biosynthetic process |
|  |  | Zardc45769 | AT3G13790 | AtCWI1 | [carbohydrate metabolic process](https://www.arabidopsis.org/servlets/TairObject?type=keyword&id=5291) |
|  |  | Zardc31601 | AT5G20250 | AtDIN10 | cellular response to hypoxia, response to oxidative stress |
|  |  |  |  |  |  |
|  | **Plant hormone  signal transduction** | Zardc02254 | AT5G13220 | AtJAS1 | regulation of jasmonic acid mediated signaling pathway |
|  |  | Zardc06900 | AT1G19180 | AtJAZ1 | regulation of jasmonic acid mediated signaling pathway |
|  |  | Zardc25044 | AT5G44080 | not found | positive regulation of transcription, DNA-templated |
|  |  | Zardc04224 | AT1G04240 | AtIAA3 | response to auxin |
|  |  | Zardc26876 | AT5G47220 | AtERF2 | ethylene-activated signaling pathway, jasmonic acid mediated signaling pathway |
|  |  | Zardc00852 | AT1G74950 | AtJAZ2 | regulation of jasmonic acid mediated signaling pathway, response to wounding |
|  |  | novel.168 | AT3G16360 | AtAHP4 | cytokinin-activated signaling pathway, phosphorelay signal transduction system |
|  |  | Zardc29777 | AT3G15540 | AtIAA19 | response to auxin, stamen filament development |
|  |  | Zardc48440 | AT1G67710 | AtARR11 | cytokinin-activated signaling pathway, response to cytokinin |
|  |  | Zardc28302 | AT2G38310 | AtPYL4 | abscisic acid-activated signaling pathway |
|  |  | Zardc09977 | AT5G05440 | AtPYL5 | abscisic acid-activated signaling pathway |
|  |  | Zardc44966 | AT4G32280 | ATIAA29 | response to auxin |
|  |  |  |  |  |  |
| **F3*vs.*F1** | **Plant hormone  signal transduction** | Zardc49467 | AT2G14580 | AtPRB1 | response to ethylene, response to jasmonic acid, response to salicylic acid |
|  |  | Zardc09485 | AT3G11410 | AtAHG3 | abscisic acid-activated signaling pathway |
|  |  | Zardc53596 | AT1G07430 | AtHAI2 | negative regulation of abscisic acid-activated signaling pathway |
|  |  | Zardc35827 | AT3G12830 | AtSAUR72 | response to salicylic acid, response to temperature stimulus |
|  |  | Zardc46850 | AT5G51760 | AtAHG1 | response to abscisic acid |
|  |  | novel.5084 | AT1G72770 | AtHAB1 | abscisic acid-activated signaling pathway |
|  |  | novel.3256 | AT2G46690 | AtSAUR32 | alpha-amino acid metabolic process, carboxylic acid biosynthetic process |
|  |  | Zardc55290 | AT2G21050 | AtLAX2 | cotyledon vascular tissue pattern formation, response to nematode |
|  |  | Zardc51967 | AT4G14550 | AtIAA14 | response to auxin |
|  |  | novel.4116 | AT3G04580 | AtEIN4 | [negative regulation of ethylene-activated signaling pathway](https://www.arabidopsis.org/servlets/TairObject?type=keyword&id=14708) |
|  |  | Zardc55291 | AT1G77690 | AtLAX3 | response to auxin, root cap development, transmembrane transport |
|  |  | Zardc13708 | AT4G33720 | AtCAPE3 | biological_process |
|  |  | Zardc26876 | AT5G47220 | AtERF2 | ethylene-activated signaling pathway, jasmonic acid mediated signaling pathway |
|  |  | Zardc26158 | AT2G14960 | AtGH3.1 | response to auxin |
|  |  |  |  |  |  |
| **F4*vs.*F3** | **Starch and sucrose metabolism** | Zardc31704 | AT4G17090 | AtBAM3 | maltose biosynthetic process, response to cold, starch catabolic process |
|  |  | Zardc54087 | AT3G18080 | AtBGLU44 | carbohydrate metabolic process |
|  |  | Zardc33027 | AT5G51820 | AtPGMP | carbohydrate metabolic process |
|  |  | Zardc06800 | AT3G20440 | AtBE1 | carbohydrate metabolic process, glycogen biosynthetic process |
|  |  | Zardc32360 | AT3G13560 | not found | carbohydrate metabolic process |
|  |  | novel.5250 | AT2G18700 | AtTPS11 | cellular amino acid metabolic process, cellular catabolic process |
|  |  | Zardc31272 | AT4G12430 | AtTPPF | Haloacid dehalogenase-like hydrolase (HAD) superfamily protein |
|  |  | Zardc27757 | AT1G66250 | not found | carbohydrate metabolic process |
|  |  | Zardc45769 | AT3G13790 | AtBFRUCT1 | carbohydrate metabolic process |
|  |  | Zardc33026 | AT5G51830 | AtFRK1 | [starch biosynthetic process](https://www.arabidopsis.org/servlets/TairObject?type=keyword&id=10497) |
|  |  | Zardc01552 | AT1G70710 | AtGH9B1 | chloroplast, extracellular region |
|  |  | Zardc31270 | AT1G62660 | AtVI1 | carbohydrate metabolic process |
|  |  | Zardc31209 | AT1G02800 | AtCEL2 | pattern specification process, response to nematode |
|  |  | Zardc11194 | AT5G10100 | AtTPPI | trehalose biosynthetic process |
|  |  | Zardc34000 | AT3G23920 | AtBAM1 | response to water deprivation, starch catabolic process |
|  |  | Zardc12008 | AT5G03650 | AtSBE2.2 | carbohydrate metabolic process, glycogen biosynthetic process |
|  |  | Zardc32583 | AT4G10120 | AtSPSC | sucrose biosynthetic process |
|  |  |  |  |  |  |
|  | **Galactose metabolism** | Zardc07320 | AT1G55740 | AtSIP1 | flavonoid metabolic process, root development |
|  |  | Zardc33027 | AT5G51820 | AtPGMP | carbohydrate metabolic process |
|  |  | Zardc23456 | AT5G40390 | AtRS5 | sucrose biosynthetic process |
|  |  | Zardc45769 | AT3G13790 | AtBFRUCT1 | carbohydrate metabolic process |
|  |  | Zardc31270 | AT1G62660 | AtVI1 | carbohydrate metabolic process, sucrose metabolic process |
|  |  |  |  |  |  |
| **M1vs.F1** | **Zeatin biosynthesis** | Zardc45722 | AT1G22400.1 | AtUGT85A1 | alpha-amino acid metabolic process, cellular catabolic process |
|  |  |  |  |  |  |
|  | **Plant hormone signal  transduction** | Zardc26876 | AT5G47220.1 | AtERF2 | ethylene-activated signaling pathway |
|  |  | Zardc39292 | AT5G45110.1 | AtNPR3 | regulation of jasmonic acid mediated signaling pathway |
|  |  | novel.4197 | AT2G14580.1 | AtPRB1 | response to ethylene, response to jasmonic acid, response to salicylic acid |
|  |  |  |  |  |  |
|  | **Starch and sucrose  metabolism** | Zardc44219 | AT1G11820 | no name | carbohydrate metabolic process |
|  |  | Zardc27757 | AT1G66250 | no name | carbohydrate metabolic process |
|  |  | Zardc21370 | AT2G44540 | AtGH9B9 | extracellular region, cellulase activity |
|  |  | Zardc11215 | AT1G35910 | AtTPPD | trehalose biosynthetic process |
|  |  | Zardc34000 | AT3G23920 | AtBAM1 | response to water deprivation, starch catabolic process |
|  |  | novel.1016 | AT4G00490 | AtBAM2 | meristem development, terpenoid biosynthetic process |
|  |  |  |  |  |  |
| **M2vs.F2** | **Zeatin biosynthesis** | Zardc24534 | AT1G75450.1 | AtCKX5 | cytokinin metabolic process |
|  |  | Zardc46645 | AT5G56970 | AtCKX3 | cytokinin metabolic process |
|  |  | Zardc36944 | AT2G41510 | AtCKX1 | cytokinin metabolic process |
|  |  | novel.3902 | AT3G63440 | AtCKX6 | cytokinin metabolic process |
|  |  |  |  |  |  |
|  | **Plant hormone signal transduction** | Zardc22092 | AT1G04550.2 | AtIAA12 | embryonic pattern specification, response to auxin, root development |
|  |  | Zardc04224 | [AT1G04240.1](https://www.arabidopsis.org/servlets/TairObject?type=gene&id=29724) | AtIAA3 | response to auxin |
|  |  | Zardc45890 | [AT5G20730.1](https://www.arabidopsis.org/servlets/TairObject?type=gene&id=136867) | AtARF7 | response to auxin, response to ethylene |
|  |  | novel.721 | [AT1G19840.1](https://www.arabidopsis.org/servlets/TairObject?type=gene&id=32089) | AtSAUR53 | cell differentiation, plant epidermis development |
|  |  | novel.4197 | [AT2G14580.1](https://www.arabidopsis.org/servlets/TairObject?type=gene&id=431959) | AtPRB1 | response to ethylene, response to jasmonic acid, response to salicylic acid |
|  |  | Zardc51803 | AT1G27320.1 | AtAHK3 | cytokinin-activated signaling pathway |
|  |  |  |  |  |  |
|  | **Starch and sucrose  metabolism** | Zardc04055 | AT1G04920.1 | AtSPSB | sucrose biosynthetic process |
|  |  | Zardc36683 | AT5G49190 | AtSUS2 | sucrose metabolic process, sucrose metabolic process |
|  |  | Zardc44219 | AT1G11820 | no name | carbohydrate metabolic process |
|  |  | Zardc21370 | AT2G44540 | AtGH9B9 | extracellular region, cellulase activity |
|  |  | Zardc11215 | AT1G35910 | AtTPPD | trehalose biosynthetic process |
|  |  | Zardc34000 | AT3G23920 | AtBAM1 | response to water deprivation, starch catabolic process |
|  |  | novel.1016 | AT4G00490 | AtBAM2 | meristem development, terpenoid biosynthetic process |
|  |  |  |  |  |  |
| **M3vs.F3** | **Zeatin biosynthesis** | Zardc46645 | AT5G56970 | AtCKX3 | cytokinin metabolic process |
|  |  | Zardc24534 | AT1G75450 | AtCKX5 | cytokinin metabolic process |
|  |  | novel.3902 | AT3G63440 | AtCKX6 | cytokinin metabolic process |
|  |  | Zardc19469 | AT1G78270 | AtUGT85A4 | glucuronosyltransferase activity, quercetin 3-O-glucosyltransferase activity |
|  |  | Zardc36944 | AT2G41510 | AtCKX1 | cytokinin metabolic process |
|  |  |  |  |  |  |
|  | **Plant hormone  signal transduction** | Zardc23082 | AT2G01830 | AtAHK4 | phosphorelay signal transduction system, regulation of meristem development |
|  |  | Zardc51803 | AT1G27320.1 | AHK3 | cytokinin-activated signaling pathway |
|  |  | Zardc55048 | AT2G01830.2 | AHK4 | phosphorelay signal transduction system, regulation of meristem development |
|  |  | Zardc11274 | AT3G57040.1 | AtARR9 | cytokinin-activated signaling pathway, phosphorelay signal transduction system |
|  |  | Zardc33161 | AT3G57040.1 | AtARR9 | cytokinin-activated signaling pathway, phosphorelay signal transduction system |
|  |  | Zardc14478 | [AT5G26594](https://www.arabidopsis.org/servlets/TairObject?type=gene&id=1000640955) | AtARR24 | expressed in flower, flower bud, fruit, pollen |
|  |  | Zardc38825 | AT1G68640 | AtTGA8 | regulation of transcription, DNA-templated, transcription, DNA-templated |
|  |  | Zardc01223 | AT5G35750 | AHK2 | cellular response to abscisic acid stimulus, regulation of flower development |
|  |  | novel.4197 | AT2G14580 | AtPRB1 | responses to ethylene and methyl jasmonate |
|  |  | Zardc26158 | AT2G14960 | GH3.1 | response to auxin |
|  |  |  |  |  |  |
|  | **Starch and sucrose metabolism** | Zardc27757 | AT1G66250 | no name | carbohydrate metabolic process |
|  |  | novel.1016 | AT4G00490 | AtBAM2 | meristem development, organic substance catabolic process |
|  |  | Zardc36683 | AT5G49190 | AtSUS2 | sucrose metabolic process, starch metabolic process, sucrose metabolic process |
|  |  | Zardc21370 | AT2G44540 | AtGH9B9 | extracellular region, cellulase activity |
|  |  | Zardc01536 | AT2G01630 | no name | carbohydrate metabolic process |
|  |  | Zardc44219 | AT1G11820 | no name | carbohydrate metabolic process |
|  |  | Zardc36911 | AT2G44480 | AtBGLU17 | [carbohydrate metabolic process](https://www.arabidopsis.org/servlets/TairObject?type=keyword&id=5291) |
|  |  | Zardc34000 | AT3G23920 | AtBAM1 | response to water deprivation, starch catabolic process |
|  |  | novel.1081 | AT1G68020 | AtTPS6 | trehalose biosynthetic process |
|  |  |  |  |  |  |
| **M4vs.F4** | **Zeatin biosynthesis** | Zardc46645 | AT5G56970 | AtCKX3 | cytokinin metabolic process |
|  |  | Zardc36944 | AT2G41510 | AtCKX1 | cytokinin metabolic process |
|  |  | novel.3902 | AT3G63440 | AtCKX6 | cytokinin metabolic process |
|  |  |  |  |  |  |
|  | **Plant hormone  signal transduction** | Zardc02254 | AT5G13220 | AtJAZ10 | regulation of jasmonic acid mediated signaling pathway |
|  |  | Zardc26868 | AT2G33860 | AtARF3 | regulation of transcription, DNA-templated |
|  |  | Zardc01223 | AT5G35750 | AtAHK2 | cellular response to abscisic acid stimulus, regulation of flower development |
|  |  | Zardc16527 | AT1G27320 | AtAHK3 | cellular response to abscisic acid stimulus, regulation of flower development |
|  |  | Zardc16532 | AT1G27320.1 | AtAHK3 | cellular response to abscisic acid stimulus, regulation of flower development |
|  |  | Zardc51803 | AT1G27320.1 | AtAHK3 | cellular response to abscisic acid stimulus, regulation of flower development |
|  |  | Zardc54051 | AT2G25180.1 | AtARR12 | cellular response to cytokinin stimulus, cytokinin-activated signaling pathway |
|  |  | Zardc46700 | AT1G59940 | AtARR3 | cytokinin-activated signaling pathway, phosphorelay signal transduction system |
|  |  | novel.168 | AT3G16360 | AtAHP4 | cytokinin-activated signaling pathway, phosphorelay signal transduction system |
|  |  | Zardc22249 | AT2G23170 | AtGH3.3 | [auxin homeostasis](https://www.arabidopsis.org/servlets/TairObject?type=keyword&id=21561) |
|  |  | Zardc52799 | AT5G54510 | AtGH3.6 | auxin homeostasis, auxin-activated signaling pathway |
|  |  | Zardc51255 | AT1G19850 | AtARF5 | flower development, meristem development, response to auxin |
|  |  | Zardc00852 | AT1G74950 | AtJAZ2 | regulation of jasmonic acid mediated signaling pathway |
|  |  | Zardc29777 | AT3G15540 | AtIAA19 | flower development, response to auxin, stamen filament development |
|  |  | Zardc03151 | AT2G41370 | AtBOP2 | jasmonic acid mediated signaling pathway, floral meristem determinacy |
|  |  |  |  |  |  |
|  | **Starch and sucrose metabolism** | novel.5250 | AT2G18700 | AtTPS11 | trehalose biosynthetic process, trehalose metabolism in response to stress |
|  |  | Zardc34000 | AT3G23920 | AtBAM1 | response to water deprivation, starch catabolic process |
|  |  | Zardc11215 | AT1G35910 | AtTPPD | trehalose biosynthetic process, response to salt stress |
|  |  | novel.1016 | AT4G00490 | AtBAM2 | meristem development, terpenoid biosynthetic process |
|  |  | Zardc36683 | AT5G49190 | AtSUS2, | sucrose metabolic process, starch metabolic process, sucrose metabolic process |
|  |  | Zardc30514 | AT5G48300 | AtAPS1 | glycogen biosynthetic process, starch biosynthetic process |
|  |  | Zardc28200 | AT3G18070 | AtBGLU43 | macromolecule catabolic process, tissue development |

**Supplementary Table 8 The FPKM of the genes selected from the KEGG enrichment**

|  | ID | M1_1 | M1_2 | M1_3 | M2_1 | M2_2 | M2_3 | M3_1 | M3_2 | M3_3 | M4_1 | M4_2 | M4_3 | F1_1 | F1_2 | F1_3 | F2_1 | F2_2 | F2_3 | F3_1 | F3_2 | F3_3 | F4_1 | F4_2 | F4_3 |
| --- | --- | --- | --- | --- | --- | --- | --- | --- | --- | --- | --- | --- | --- | --- | --- | --- | --- | --- | --- | --- | --- | --- | --- | --- | --- |
| **M3vs.M1** | Zardc46850(AHG1) | 10.70 | 13.14 | 9.36 | 32.38 | 33.28 | 34.08 | 48.14 | 42.83 | 49.52 | 42.21 | 45.56 | 42.74 | 31.63 | 32.36 | 26.11 | 41.63 | 39.47 | 37.05 | 60.38 | 64.24 | 61.06 | 29.39 | 33.02 | 31.69 |
|  | Zardc53596(AIP1) | 19.36 | 17.89 | 20.31 | 31.06 | 30.22 | 28.81 | 45.15 | 44.54 | 53.09 | 70.99 | 64.09 | 73.34 | 30.23 | 33.89 | 29.07 | 42.60 | 43.54 | 42.09 | 100.34 | 101.20 | 92.57 | 43.45 | 46.96 | 44.87 |
|  | Zardc46700(ARR3) | 27.97 | 22.97 | 25.12 | 46.24 | 45.92 | 41.99 | 63.47 | 67.35 | 75.06 | 52.35 | 46.21 | 52.66 | 39.17 | 39.13 | 30.48 | 38.35 | 39.97 | 36.76 | 53.25 | 43.85 | 41.95 | 49.93 | 48.57 | 50.40 |
|  | Zardc22092(BDL) | 2.01 | 3.95 | 2.88 | 6.30 | 6.76 | 7.30 | 7.30 | 4.44 | 6.24 | 5.48 | 5.60 | 4.82 | 3.00 | 2.81 | 2.95 | 3.58 | 3.73 | 2.53 | 2.91 | 4.15 | 5.71 | 5.03 | 4.81 | 6.01 |
|  | Zardc26158(GH3.1) | 1.05 | 1.12 | 1.41 | 1.51 | 1.93 | 1.44 | 4.02 | 4.77 | 6.00 | 0.84 | 0.75 | 0.90 | 0.88 | 0.91 | 1.48 | 0.85 | 0.85 | 0.79 | 2.27 | 2.27 | 2.23 | 1.89 | 2.11 | 1.58 |
|  | novel.5084(HAB1) | 19.95 | 22.36 | 21.02 | 40.21 | 36.68 | 43.99 | 48.55 | 45.39 | 45.18 | 52.30 | 52.55 | 59.17 | 34.45 | 31.77 | 32.03 | 41.07 | 38.26 | 37.02 | 67.28 | 76.84 | 60.29 | 44.72 | 40.89 | 40.33 |
|  | Zardc49467(PRB1) | 424.10 | 418.41 | 395.47 | 372.46 | 367.93 | 383.71 | 1474.70 | 1544.75 | 1547.31 | 487.60 | 498.98 | 551.82 | 375.88 | 331.06 | 338.92 | 607.20 | 584.25 | 690.21 | 983.13 | 1140.75 | 1143.48 | 369.13 | 335.76 | 349.88 |
|  | Zardc09485(PP2CA) | 21.77 | 22.21 | 21.72 | 44.54 | 48.43 | 44.14 | 71.06 | 76.43 | 72.79 | 58.92 | 62.60 | 56.65 | 40.88 | 39.92 | 40.18 | 45.95 | 43.07 | 43.76 | 112.76 | 106.90 | 109.15 | 58.56 | 59.00 | 50.87 |
|  | Zardc15205(IAA11) | 5.08 | 5.95 | 5.85 | 10.01 | 16.89 | 13.81 | 10.04 | 16.40 | 12.98 | 19.94 | 16.38 | 16.02 | 10.28 | 4.56 | 8.01 | 8.60 | 14.41 | 7.23 | 12.70 | 14.74 | 12.13 | 6.77 | 14.72 | 6.91 |
|  | novel.3256(SAUR32) | 3.74 | 2.77 | 3.52 | 5.99 | 6.01 | 4.89 | 8.22 | 6.61 | 7.11 | 2.54 | 2.89 | 3.08 | 4.90 | 4.59 | 4.63 | 4.39 | 4.89 | 4.33 | 13.21 | 13.50 | 13.21 | 8.87 | 7.33 | 6.28 |
|  | novel.1294(SAUR52) | 1.41 | 1.39 | 1.01 | 1.75 | 2.32 | 2.19 | 2.17 | 2.27 | 3.48 | 3.40 | 2.51 | 1.49 | 1.73 | 0.96 | 1.18 | 2.24 | 2.27 | 2.83 | 1.08 | 2.73 | 3.39 | 5.32 | 4.24 | 4.49 |
|  | Zardc35827(SAUR72) | 8.51 | 7.51 | 5.26 | 28.64 | 28.37 | 32.31 | 28.09 | 35.01 | 28.67 | 26.76 | 24.62 | 26.99 | 19.89 | 21.66 | 16.42 | 33.65 | 29.58 | 27.78 | 55.82 | 63.51 | 60.16 | 41.78 | 35.12 | 39.28 |
|  |  |  |  |  |  |  |  |  |  |  |  |  |  |  |  |  |  |  |  |  |  |  |  |  |  |
| **M4vs.M3** | Zardc08999(AMY2) | 3.78 | 3.91 | 2.73 | 5.26 | 5.01 | 4.62 | 1.20 | 1.77 | 1.56 | 3.84 | 2.79 | 3.72 | 5.08 | 2.80 | 3.91 | 3.06 | 3.54 | 4.08 | 2.53 | 1.83 | 1.70 | 4.05 | 3.95 | 3.07 |
|  | Zardc48948(APL3) | 14.53 | 12.02 | 14.70 | 30.95 | 28.78 | 28.50 | 25.19 | 22.19 | 23.16 | 49.12 | 48.37 | 47.11 | 19.80 | 16.98 | 17.35 | 31.75 | 32.76 | 32.22 | 37.77 | 38.92 | 44.38 | 41.23 | 37.12 | 43.45 |
|  | Zardc34000(BAM1) | 1.13 | 1.55 | 1.34 | 2.51 | 2.75 | 1.79 | 4.02 | 2.25 | 3.80 | 17.72 | 18.60 | 19.28 | 0.39 | 0.45 | 0.20 | 0.11 | 0.09 | 0.29 | 0.05 | 0.00 | 0.57 | 6.60 | 9.28 | 9.40 |
|  | Zardc31704(BAM3) | 3.09 | 3.19 | 2.66 | 4.03 | 3.08 | 3.59 | 0.72 | 1.15 | 1.03 | 31.54 | 30.84 | 31.05 | 1.27 | 1.41 | 1.85 | 2.23 | 2.16 | 1.58 | 1.38 | 0.88 | 1.03 | 39.46 | 46.91 | 43.43 |
|  | Zardc02022(BAM7) | 0.00 | 0.23 | 0.33 | 0.89 | 0.11 | 0.58 | 0.13 | 0.00 | 0.61 | 1.45 | 1.88 | 0.66 | 0.00 | 0.00 | 0.00 | 0.21 | 0.12 | 0.22 | 0.13 | 0.00 | 0.25 | 0.33 | 0.45 | 0.23 |
|  | Zardc45769(CWI1) | 2.30 | 1.53 | 2.32 | 2.96 | 3.28 | 3.81 | 2.91 | 4.13 | 4.36 | 7.03 | 8.67 | 8.57 | 2.24 | 2.01 | 1.79 | 3.31 | 3.95 | 3.87 | 4.72 | 3.10 | 4.45 | 10.75 | 7.71 | 10.10 |
|  | Zardc33026(FRK1) | 7.85 | 9.19 | 9.12 | 7.67 | 7.55 | 8.57 | 7.00 | 4.56 | 5.73 | 21.37 | 21.24 | 24.32 | 6.61 | 7.96 | 6.57 | 9.21 | 9.03 | 7.84 | 6.92 | 7.74 | 7.52 | 14.71 | 17.50 | 14.56 |
|  | Zardc33027(PGMP) | 16.96 | 17.46 | 19.39 | 32.48 | 31.99 | 32.20 | 34.36 | 36.66 | 36.52 | 81.34 | 88.85 | 83.18 | 13.86 | 14.03 | 16.19 | 28.22 | 28.04 | 27.85 | 24.45 | 24.46 | 23.62 | 50.68 | 51.20 | 48.00 |
|  | Zardc12008(SBE2.2) | 0.94 | 0.29 | 0.35 | 3.61 | 1.38 | 2.40 | 2.43 | 1.23 | 1.16 | 4.03 | 6.29 | 3.69 | 0.38 | 0.38 | 1.02 | 1.50 | 1.67 | 2.53 | 1.37 | 0.50 | 1.06 | 2.35 | 2.55 | 10.08 |
|  | Zardc10599(TPS9) | 3.66 | 4.84 | 4.31 | 4.13 | 3.37 | 3.70 | 4.83 | 5.66 | 4.79 | 11.84 | 11.07 | 10.44 | 5.85 | 6.24 | 5.98 | 2.12 | 3.64 | 2.94 | 4.29 | 5.71 | 5.99 | 7.45 | 6.43 | 6.58 |
|  | novel.5250(TPS11) | 0.93 | 1.36 | 1.11 | 0.85 | 1.34 | 0.77 | 3.11 | 3.82 | 3.45 | 18.47 | 17.36 | 19.33 | 1.90 | 2.37 | 2.50 | 1.60 | 1.26 | 1.27 | 3.04 | 3.33 | 3.46 | 7.72 | 8.63 | 8.33 |
|  | Zardc08022(TPS11) | 1.57 | 1.98 | 2.21 | 1.36 | 1.68 | 1.89 | 5.04 | 4.82 | 5.09 | 11.87 | 11.00 | 12.02 | 4.91 | 5.53 | 4.50 | 2.49 | 2.29 | 1.99 | 7.34 | 7.57 | 7.35 | 12.28 | 12.52 | 12.39 |
|  | Zardc31272(TPPF) | 1.88 | 1.81 | 3.12 | 2.27 | 1.81 | 1.97 | 1.27 | 1.28 | 1.69 | 6.59 | 6.98 | 9.57 | 1.39 | 2.31 | 1.11 | 1.60 | 1.41 | 1.43 | 0.50 | 1.09 | 0.68 | 5.79 | 3.77 | 5.12 |
|  | Zardc11215(TPPD) | 1.20 | 1.05 | 0.81 | 1.59 | 0.83 | 0.97 | 1.06 | 1.01 | 0.13 | 5.32 | 7.36 | 6.84 | 0.25 | 0.12 | 0.23 | 0.22 | 0.32 | 0.12 | 0.35 | 0.19 | 0.40 | 0.41 | 1.53 | 1.17 |
|  | Zardc11194(TPPI) | 1.32 | 1.42 | 0.99 | 1.12 | 0.54 | 1.22 | 1.19 | 1.65 | 1.67 | 3.62 | 3.92 | 3.49 | 1.13 | 0.82 | 1.27 | 0.88 | 0.70 | 1.06 | 1.26 | 1.40 | 1.01 | 3.80 | 3.02 | 4.70 |
|  | Zardc31601(DIN10) | 1.54 | 2.48 | 1.66 | 1.86 | 1.71 | 1.92 | 1.33 | 2.35 | 2.20 | 4.57 | 6.69 | 3.82 | 1.07 | 1.34 | 3.14 | 3.13 | 1.99 | 1.85 | 2.01 | 3.63 | 3.28 | 2.51 | 3.72 | 4.07 |
|  | Zardc23456(RS5) | 9.88 | 9.45 | 9.81 | 12.77 | 12.86 | 12.43 | 5.22 | 4.40 | 3.70 | 17.30 | 16.15 | 16.99 | 6.49 | 6.26 | 5.75 | 12.44 | 13.86 | 13.74 | 6.31 | 5.32 | 7.10 | 11.93 | 13.71 | 12.78 |
|  | Zardc03128(SIP2) | 62.06 | 81.75 | 66.48 | 69.57 | 70.56 | 66.49 | 83.79 | 88.73 | 74.67 | 215.26 | 212.72 | 212.97 | 56.90 | 56.18 | 60.69 | 30.82 | 33.96 | 33.11 | 59.95 | 56.46 | 66.03 | 81.40 | 92.26 | 85.28 |
|  | Zardc04821(STS) | 2.15 | 1.96 | 1.85 | 5.77 | 5.62 | 5.19 | 2.65 | 2.65 | 3.39 | 15.98 | 16.77 | 16.76 | 2.68 | 2.05 | 1.91 | 4.38 | 5.61 | 4.46 | 4.00 | 3.24 | 2.85 | 4.00 | 3.78 | 4.40 |
|  | Zardc33027(PGMP) | 16.96 | 17.46 | 19.39 | 32.48 | 31.99 | 32.20 | 34.36 | 36.66 | 36.52 | 81.34 | 88.85 | 83.18 | 13.86 | 14.03 | 16.19 | 28.22 | 28.04 | 27.85 | 24.45 | 24.46 | 23.62 | 50.68 | 51.20 | 48.00 |
|  | Zardc45769(CWI1) | 2.30 | 1.53 | 2.32 | 2.96 | 3.28 | 3.81 | 2.91 | 4.13 | 4.36 | 7.03 | 8.67 | 8.57 | 2.24 | 2.01 | 1.79 | 3.31 | 3.95 | 3.87 | 4.72 | 3.10 | 4.45 | 10.75 | 7.71 | 10.10 |
|  | novel.168(AHP4) | 0.00 | 0.00 | 0.00 | 0.00 | 0.00 | 0.00 | 0.00 | 0.00 | 0.00 | 2.68 | 3.03 | 1.60 | 0.00 | 0.00 | 0.00 | 0.00 | 0.00 | 0.00 | 0.00 | 0.00 | 0.00 | 0.00 | 0.00 | 0.00 |
|  | Zardc48440(ARR11) | 3.35 | 3.11 | 3.59 | 7.68 | 7.60 | 8.01 | 4.41 | 3.67 | 2.97 | 8.80 | 6.83 | 8.12 | 1.63 | 2.83 | 3.12 | 8.19 | 7.99 | 7.09 | 3.45 | 4.35 | 3.84 | 7.83 | 6.73 | 8.47 |
|  | Zardc26876(ERF2) | 8.40 | 9.40 | 8.30 | 8.69 | 10.44 | 10.95 | 10.53 | 6.85 | 7.38 | 27.11 | 32.15 | 27.12 | 4.03 | 5.40 | 3.32 | 4.91 | 5.97 | 6.36 | 9.85 | 8.48 | 7.46 | 15.72 | 14.28 | 18.63 |
|  | Zardc02254(JAS1) | 52.53 | 38.36 | 51.06 | 38.81 | 32.67 | 36.78 | 38.84 | 42.89 | 37.58 | 234.29 | 229.24 | 240.61 | 46.01 | 33.09 | 42.33 | 25.18 | 25.19 | 28.16 | 32.57 | 31.12 | 31.48 | 101.98 | 104.12 | 98.60 |
|  | Zardc06900(JAZ1) | 146.79 | 146.36 | 150.08 | 91.39 | 95.72 | 94.56 | 58.41 | 62.34 | 64.37 | 187.56 | 179.29 | 188.56 | 128.30 | 118.62 | 132.41 | 91.15 | 102.35 | 99.79 | 85.92 | 79.60 | 83.94 | 178.93 | 179.48 | 170.04 |
|  | Zardc04224(IAA3) | 11.73 | 10.96 | 10.91 | 12.18 | 10.56 | 10.54 | 17.05 | 21.35 | 17.17 | 60.26 | 52.49 | 58.23 | 12.14 | 11.61 | 13.15 | 4.96 | 5.87 | 4.95 | 24.54 | 26.91 | 21.05 | 37.29 | 34.76 | 31.52 |
|  | Zardc29777(IAA19) | 3.12 | 5.43 | 4.41 | 3.21 | 4.24 | 2.31 | 3.14 | 3.90 | 2.28 | 10.24 | 9.56 | 11.28 | 5.04 | 3.75 | 4.11 | 1.82 | 4.20 | 3.34 | 2.99 | 4.21 | 3.52 | 7.19 | 3.22 | 4.69 |
|  | Zardc44966(IAA29) | 0.68 | 0.12 | 0.33 | 2.02 | 1.71 | 1.16 | 0.89 | 0.85 | 0.37 | 2.03 | 2.45 | 2.88 | 0.96 | 0.11 | 0.44 | 0.63 | 0.84 | 1.12 | 0.40 | 0.97 | 0.90 | 1.00 | 1.13 | 1.18 |
|  | Zardc00852(JAZ2) | 6.89 | 6.29 | 5.65 | 2.91 | 3.92 | 3.19 | 2.67 | 3.78 | 3.45 | 11.76 | 10.92 | 10.32 | 5.15 | 3.85 | 9.75 | 4.01 | 2.84 | 3.78 | 3.14 | 4.27 | 3.83 | 2.88 | 5.74 | 7.39 |
|  | Zardc28302(PYL4) | 1.12 | 1.45 | 0.82 | 0.46 | 0.09 | 0.38 | 0.00 | 0.00 | 0.20 | 2.31 | 1.91 | 0.99 | 0.59 | 0.55 | 0.54 | 0.17 | 0.20 | 0.00 | 1.20 | 0.49 | 0.00 | 3.28 | 4.51 | 2.79 |
|  | Zardc09977(PYL5) | 2.49 | 0.86 | 1.21 | 0.55 | 0.83 | 0.56 | 0.15 | 0.29 | 0.45 | 3.01 | 1.22 | 2.28 | 1.59 | 1.76 | 1.07 | 1.14 | 1.17 | 0.14 | 0.32 | 1.32 | 1.25 | 2.16 | 1.92 | 3.29 |
|  | Zardc25044 | 35.87 | 35.34 | 38.31 | 79.07 | 81.53 | 76.82 | 70.02 | 70.80 | 74.08 | 143.15 | 149.85 | 149.54 | 44.31 | 44.14 | 41.22 | 71.30 | 67.69 | 70.83 | 82.29 | 80.25 | 88.70 | 72.20 | 90.85 | 86.01 |
|  |  |  |  |  |  |  |  |  |  |  |  |  |  |  |  |  |  |  |  |  |  |  |  |  |  |
| **F3vs.F1** | Zardc49467(CAPE7) | 424.10 | 418.41 | 395.47 | 372.46 | 367.93 | 383.71 | 1474.70 | 1544.75 | 1547.31 | 487.60 | 498.98 | 551.82 | 375.88 | 331.06 | 338.92 | 607.20 | 584.25 | 690.21 | 983.13 | 1140.75 | 1143.48 | 369.13 | 335.76 | 349.88 |
|  | Zardc09485(AHG3) | 21.77 | 22.21 | 21.72 | 44.54 | 48.43 | 44.14 | 71.06 | 76.43 | 72.79 | 58.92 | 62.60 | 56.65 | 40.88 | 39.92 | 40.18 | 45.95 | 43.07 | 43.76 | 112.76 | 106.90 | 109.15 | 58.56 | 59.00 | 50.87 |
|  | Zardc53596(AIP1) | 19.36 | 17.89 | 20.31 | 31.06 | 30.22 | 28.81 | 45.15 | 44.54 | 53.09 | 70.99 | 64.09 | 73.34 | 30.23 | 33.89 | 29.07 | 42.60 | 43.54 | 42.09 | 100.34 | 101.20 | 92.57 | 43.45 | 46.96 | 44.87 |
|  | Zardc35827(SAUR72) | 8.51 | 7.51 | 5.26 | 28.64 | 28.37 | 32.31 | 28.09 | 35.01 | 28.67 | 26.76 | 24.62 | 26.99 | 19.89 | 21.66 | 16.42 | 33.65 | 29.58 | 27.78 | 55.82 | 63.51 | 60.16 | 41.78 | 35.12 | 39.28 |
|  | Zardc46850(AHG1) | 10.70 | 13.14 | 9.36 | 32.38 | 33.28 | 34.08 | 48.14 | 42.83 | 49.52 | 42.21 | 45.56 | 42.74 | 31.63 | 32.36 | 26.11 | 41.63 | 39.47 | 37.05 | 60.38 | 64.24 | 61.06 | 29.39 | 33.02 | 31.69 |
|  | novel.5084(HAB1) | 19.95 | 22.36 | 21.02 | 40.21 | 36.68 | 43.99 | 48.55 | 45.39 | 45.18 | 52.30 | 52.55 | 59.17 | 34.45 | 31.77 | 32.03 | 41.07 | 38.26 | 37.02 | 67.28 | 76.84 | 60.29 | 44.72 | 40.89 | 40.33 |
|  | novel.3256(SAUR32) | 3.74 | 2.77 | 3.52 | 5.99 | 6.01 | 4.89 | 8.22 | 6.61 | 7.11 | 2.54 | 2.89 | 3.08 | 4.90 | 4.59 | 4.63 | 4.39 | 4.89 | 4.33 | 13.21 | 13.50 | 13.21 | 8.87 | 7.33 | 6.28 |
|  | Zardc55290(LAX2) | 6.54 | 7.99 | 6.35 | 6.83 | 8.10 | 7.77 | 15.91 | 13.73 | 10.19 | 8.82 | 7.45 | 7.68 | 9.32 | 7.76 | 9.18 | 9.51 | 9.84 | 8.75 | 33.20 | 23.86 | 20.66 | 17.68 | 18.19 | 18.82 |
|  | Zardc51967(IAA14) | 11.07 | 14.28 | 12.84 | 12.31 | 11.14 | 12.22 | 14.96 | 17.30 | 16.76 | 18.08 | 18.45 | 19.11 | 14.55 | 15.92 | 12.65 | 19.22 | 20.93 | 14.46 | 29.64 | 31.90 | 34.81 | 19.42 | 21.36 | 25.90 |
|  | novel.4116(EIN4) | 2.93 | 2.88 | 3.66 | 6.59 | 5.62 | 6.13 | 5.33 | 4.46 | 6.21 | 6.77 | 6.68 | 6.86 | 2.60 | 2.43 | 2.90 | 8.87 | 7.07 | 7.16 | 7.14 | 7.58 | 6.87 | 4.77 | 5.20 | 6.45 |
|  | Zardc55291(LAX3) | 6.13 | 4.96 | 5.96 | 6.59 | 5.78 | 4.55 | 9.51 | 8.14 | 8.72 | 6.28 | 5.55 | 6.27 | 7.00 | 5.79 | 6.24 | 8.26 | 5.77 | 6.78 | 14.37 | 14.43 | 13.70 | 12.83 | 11.34 | 8.98 |
|  | Zardc13708(CAPE3) | 0.72 | 0.00 | 0.00 | 0.70 | 0.71 | 0.91 | 1.78 | 0.38 | 1.34 | 0.00 | 0.00 | 0.17 | 0.00 | 0.00 | 0.00 | 0.49 | 0.00 | 0.35 | 0.42 | 1.71 | 3.43 | 0.17 | 1.77 | 0.55 |
|  | Zardc26876(ERF2) | 8.40 | 9.40 | 8.30 | 8.69 | 10.44 | 10.95 | 10.53 | 6.85 | 7.38 | 27.11 | 32.15 | 27.12 | 4.03 | 5.40 | 3.32 | 4.91 | 5.97 | 6.36 | 9.85 | 8.48 | 7.46 | 15.72 | 14.28 | 18.63 |
|  | Zardc26158(GH3.1) | 1.05 | 1.12 | 1.41 | 1.51 | 1.93 | 1.44 | 4.02 | 4.77 | 6.00 | 0.84 | 0.75 | 0.90 | 0.88 | 0.91 | 1.48 | 0.85 | 0.85 | 0.79 | 2.27 | 2.27 | 2.23 | 1.89 | 2.11 | 1.58 |
|  |  |  |  |  |  |  |  |  |  |  |  |  |  |  |  |  |  |  |  |  |  |  |  |  |  |
| **F4vs.F3** | Zardc31704(BAM3) | 3.09 | 3.19 | 2.66 | 4.03 | 3.08 | 3.59 | 0.72 | 1.15 | 1.03 | 31.54 | 30.84 | 31.05 | 1.27 | 1.41 | 1.85 | 2.23 | 2.16 | 1.58 | 1.38 | 0.88 | 1.03 | 39.46 | 46.91 | 43.43 |
|  | Zardc54087(BGLU44) | 153.62 | 139.52 | 159.13 | 49.72 | 41.48 | 51.30 | 63.41 | 65.91 | 60.31 | 42.40 | 42.16 | 36.05 | 116.28 | 102.44 | 114.59 | 55.73 | 55.85 | 54.56 | 49.16 | 50.51 | 42.19 | 129.59 | 116.86 | 122.03 |
|  | Zardc33027(PGMP) | 16.96 | 17.46 | 19.39 | 32.48 | 31.99 | 32.20 | 34.36 | 36.66 | 36.52 | 81.34 | 88.85 | 83.18 | 13.86 | 14.03 | 16.19 | 28.22 | 28.04 | 27.85 | 24.45 | 24.46 | 23.62 | 50.68 | 51.20 | 48.00 |
|  | Zardc06800(BE1) | 13.16 | 13.27 | 12.69 | 11.12 | 11.74 | 11.24 | 12.63 | 13.21 | 11.10 | 21.65 | 22.06 | 18.62 | 9.83 | 9.31 | 10.85 | 10.84 | 12.49 | 11.74 | 6.99 | 7.12 | 8.35 | 16.13 | 17.96 | 15.89 |
|  | Zardc32360 | 22.52 | 21.37 | 20.49 | 21.15 | 21.21 | 21.78 | 14.16 | 13.26 | 13.06 | 18.87 | 19.56 | 16.95 | 13.74 | 13.20 | 13.16 | 19.77 | 21.42 | 19.07 | 8.31 | 6.89 | 6.14 | 18.12 | 19.43 | 18.97 |
|  | novel.5250(TPS11) | 0.93 | 1.36 | 1.11 | 0.85 | 1.34 | 0.77 | 3.11 | 3.82 | 3.45 | 18.47 | 17.36 | 19.33 | 1.90 | 2.37 | 2.50 | 1.60 | 1.26 | 1.27 | 3.04 | 3.33 | 3.46 | 7.72 | 8.63 | 8.33 |
|  | Zardc31272(TPPF) | 1.88 | 1.81 | 3.12 | 2.27 | 1.81 | 1.97 | 1.27 | 1.28 | 1.69 | 6.59 | 6.98 | 9.57 | 1.39 | 2.31 | 1.11 | 1.60 | 1.41 | 1.43 | 0.50 | 1.09 | 0.68 | 5.79 | 3.77 | 5.12 |
|  | Zardc27757 | 26.07 | 23.80 | 25.06 | 15.82 | 13.49 | 13.31 | 15.68 | 16.89 | 13.99 | 8.67 | 8.98 | 8.60 | 13.56 | 11.48 | 11.96 | 14.38 | 14.05 | 11.88 | 5.19 | 5.51 | 5.59 | 11.85 | 11.20 | 11.32 |
|  | Zardc45769(CWI1) | 2.30 | 1.53 | 2.32 | 2.96 | 3.28 | 3.81 | 2.91 | 4.13 | 4.36 | 7.03 | 8.67 | 8.57 | 2.24 | 2.01 | 1.79 | 3.31 | 3.95 | 3.87 | 4.72 | 3.10 | 4.45 | 10.75 | 7.71 | 10.10 |
|  | Zardc33026(FRK1) | 7.85 | 9.19 | 9.12 | 7.67 | 7.55 | 8.57 | 7.00 | 4.56 | 5.73 | 21.37 | 21.24 | 24.32 | 6.61 | 7.96 | 6.57 | 9.21 | 9.03 | 7.84 | 6.92 | 7.74 | 7.52 | 14.71 | 17.50 | 14.56 |
|  | Zardc01552(GH9B1) | 16.37 | 14.33 | 15.72 | 5.36 | 3.84 | 3.19 | 2.88 | 4.43 | 4.49 | 3.76 | 4.98 | 4.62 | 8.81 | 9.95 | 8.59 | 7.22 | 5.59 | 6.66 | 4.63 | 3.17 | 2.23 | 7.46 | 8.37 | 9.49 |
|  | Zardc31270(VI1) | 8.27 | 6.43 | 8.26 | 1.57 | 2.95 | 2.24 | 4.20 | 4.25 | 4.47 | 7.50 | 3.26 | 5.15 | 4.44 | 4.53 | 3.88 | 1.80 | 3.61 | 2.01 | 2.67 | 4.02 | 4.53 | 8.80 | 11.01 | 9.16 |
|  | Zardc31209(CEL2) | 3.83 | 4.91 | 4.85 | 1.96 | 1.88 | 2.27 | 0.91 | 1.23 | 1.56 | 1.82 | 2.22 | 1.60 | 2.68 | 2.37 | 3.04 | 2.13 | 2.05 | 2.99 | 1.08 | 1.70 | 3.71 | 6.99 | 5.83 | 5.70 |
|  | Zardc11194(TPPI) | 1.32 | 1.42 | 0.99 | 1.12 | 0.54 | 1.22 | 1.19 | 1.65 | 1.67 | 3.62 | 3.92 | 3.49 | 1.13 | 0.82 | 1.27 | 0.88 | 0.70 | 1.06 | 1.26 | 1.40 | 1.01 | 3.80 | 3.02 | 4.70 |
|  | Zardc34000(BAM1) | 1.13 | 1.55 | 1.34 | 2.51 | 2.75 | 1.79 | 4.02 | 2.25 | 3.80 | 17.72 | 18.60 | 19.28 | 0.39 | 0.45 | 0.20 | 0.11 | 0.09 | 0.29 | 0.05 | 0.00 | 0.57 | 6.60 | 9.28 | 9.40 |
|  | Zardc12008(SBE2) | 0.94 | 0.29 | 0.35 | 3.61 | 1.38 | 2.40 | 2.43 | 1.23 | 1.16 | 4.03 | 6.29 | 3.69 | 0.38 | 0.38 | 1.02 | 1.50 | 1.67 | 2.53 | 1.37 | 0.50 | 1.06 | 2.35 | 2.55 | 10.08 |
|  | Zardc32583(SPS4F) | 0.33 | 0.51 | 0.59 | 0.55 | 0.42 | 0.31 | 0.34 | 0.37 | 0.68 | 0.42 | 0.66 | 0.77 | 0.39 | 0.37 | 0.45 | 0.53 | 0.42 | 0.64 | 0.33 | 0.42 | 0.29 | 1.05 | 0.76 | 0.82 |
|  | Zardc07320(SIP1) | 310.31 | 317.61 | 304.03 | 128.78 | 126.91 | 130.97 | 84.50 | 78.93 | 84.41 | 44.43 | 43.11 | 43.30 | 180.10 | 191.59 | 176.20 | 171.05 | 170.59 | 171.22 | 42.71 | 42.09 | 39.38 | 109.69 | 116.18 | 112.09 |
|  | Zardc23456(RS5) | 9.88 | 9.45 | 9.81 | 12.77 | 12.86 | 12.43 | 5.22 | 4.40 | 3.70 | 17.30 | 16.15 | 16.99 | 6.49 | 6.26 | 5.75 | 12.44 | 13.86 | 13.74 | 6.31 | 5.32 | 7.10 | 11.93 | 13.71 | 12.78 |
|  | Zardc33027(PGMP) | 16.96 | 17.46 | 19.39 | 32.48 | 31.99 | 32.20 | 34.36 | 36.66 | 36.52 | 81.34 | 88.85 | 83.18 | 13.86 | 14.03 | 16.19 | 28.22 | 28.04 | 27.85 | 24.45 | 24.46 | 23.62 | 50.68 | 51.20 | 48.00 |
|  | Zardc45769(CWI1) | 2.30 | 1.53 | 2.32 | 2.96 | 3.28 | 3.81 | 2.91 | 4.13 | 4.36 | 7.03 | 8.67 | 8.57 | 2.24 | 2.01 | 1.79 | 3.31 | 3.95 | 3.87 | 4.72 | 3.10 | 4.45 | 10.75 | 7.71 | 10.10 |
|  | Zardc31270(VI1) | 8.27 | 6.43 | 8.26 | 1.57 | 2.95 | 2.24 | 4.20 | 4.25 | 4.47 | 7.50 | 3.26 | 5.15 | 4.44 | 4.53 | 3.88 | 1.80 | 3.61 | 2.01 | 2.67 | 4.02 | 4.53 | 8.80 | 11.01 | 9.16 |
|  |  |  |  |  |  |  |  |  |  |  |  |  |  |  |  |  |  |  |  |  |  |  |  |  |  |
| **M1vs.F1** | Zardc45722(UGT85A1) | 2.28 | 1.82 | 2.17 | 2.16 | 2.51 | 2.91 | 3.58 | 2.57 | 2.40 | 2.12 | 2.51 | 3.09 | 0.95 | 1.07 | 0.46 | 0.83 | 1.21 | 2.05 | 1.67 | 1.01 | 1.77 | 2.55 | 2.25 | 1.82 |
|  | Zardc26876(ERF2) | 8.40 | 9.40 | 8.30 | 8.69 | 10.44 | 10.95 | 10.53 | 6.85 | 7.38 | 27.11 | 32.15 | 27.12 | 4.03 | 5.40 | 3.32 | 4.91 | 5.97 | 6.36 | 9.85 | 8.48 | 7.46 | 15.72 | 14.28 | 18.63 |
|  | Zardc39292(NPR3) | 2.45 | 2.28 | 2.49 | 1.59 | 2.48 | 0.68 | 0.85 | 2.41 | 0.60 | 2.67 | 2.91 | 1.67 | 0.81 | 0.93 | 0.88 | 0.90 | 1.44 | 0.48 | 1.27 | 0.82 | 1.42 | 1.78 | 2.60 | 1.92 |
|  | novel.4197(PRB1) | 10.10 | 9.63 | 9.60 | 7.40 | 9.95 | 7.55 | 2.73 | 5.10 | 4.57 | 1.00 | 1.42 | 1.31 | 0.47 | 0.11 | 0.76 | 0.00 | 1.07 | 0.33 | 0.53 | 0.48 | 1.01 | 0.77 | 1.00 | 0.58 |
|  | Zardc44219 | 2.43 | 2.27 | 2.13 | 2.30 | 2.18 | 1.90 | 1.39 | 1.48 | 3.45 | 0.42 | 0.68 | 1.81 | 0.24 | 0.32 | 0.27 | 1.11 | 0.94 | 0.46 | 0.49 | 0.49 | 0.05 | 0.32 | 0.41 | 0.29 |
|  | Zardc27757 | 26.07 | 23.80 | 25.06 | 15.82 | 13.49 | 13.31 | 15.68 | 16.89 | 13.99 | 8.67 | 8.98 | 8.60 | 13.56 | 11.48 | 11.96 | 14.38 | 14.05 | 11.88 | 5.19 | 5.51 | 5.59 | 11.85 | 11.20 | 11.32 |
|  | Zardc21370(GH9B9) | 0.64 | 1.18 | 1.20 | 2.43 | 2.56 | 2.42 | 0.66 | 0.78 | 2.28 | 0.00 | 0.00 | 0.00 | 0.00 | 0.00 | 0.00 | 0.00 | 0.00 | 0.00 | 0.00 | 0.00 | 0.00 | 0.10 | 0.05 | 0.00 |
|  | Zardc11215(TPPD) | 1.20 | 1.05 | 0.81 | 1.59 | 0.83 | 0.97 | 1.06 | 1.01 | 0.13 | 5.32 | 7.36 | 6.84 | 0.25 | 0.12 | 0.23 | 0.22 | 0.32 | 0.12 | 0.35 | 0.19 | 0.40 | 0.41 | 1.53 | 1.17 |
|  | Zardc34000(BAM1) | 1.13 | 1.55 | 1.34 | 2.51 | 2.75 | 1.79 | 4.02 | 2.25 | 3.80 | 17.72 | 18.60 | 19.28 | 0.39 | 0.45 | 0.20 | 0.11 | 0.09 | 0.29 | 0.05 | 0.00 | 0.57 | 6.60 | 9.28 | 9.40 |
|  | novel.1016(BAM2) | 3.04 | 2.38 | 2.62 | 4.26 | 3.92 | 4.13 | 3.80 | 3.39 | 3.01 | 3.56 | 3.84 | 3.16 | 0.00 | 0.00 | 0.00 | 0.00 | 0.00 | 0.00 | 0.00 | 0.00 | 0.00 | 0.00 | 0.00 | 0.00 |
|  |  |  |  |  |  |  |  |  |  |  |  |  |  |  |  |  |  |  |  |  |  |  |  |  |  |
| **M2vs.F2** | Zardc24534(CKX5) | 43.79 | 48.90 | 48.80 | 22.89 | 24.13 | 20.53 | 91.76 | 94.01 | 93.32 | 32.37 | 36.96 | 36.34 | 31.05 | 27.40 | 29.94 | 6.03 | 6.48 | 5.93 | 39.27 | 34.58 | 35.92 | 80.48 | 87.58 | 87.72 |
|  | Zardc46645(CKX3) | 141.94 | 130.74 | 136.04 | 56.24 | 58.75 | 56.37 | 92.41 | 97.63 | 98.51 | 17.64 | 16.73 | 17.39 | 128.71 | 128.10 | 139.26 | 1.60 | 1.45 | 1.57 | 3.32 | 3.40 | 3.09 | 1.29 | 0.98 | 0.73 |
|  | Zardc36944(CKX1) | 0.72 | 0.19 | 0.87 | 0.71 | 1.43 | 1.01 | 0.50 | 1.23 | 0.48 | 0.44 | 0.88 | 0.52 | 0.09 | 0.09 | 0.00 | 0.00 | 0.00 | 0.09 | 0.00 | 0.10 | 0.00 | 0.00 | 0.00 | 0.00 |
|  | novel.3902(CKX6) | 0.43 | 0.91 | 1.06 | 2.99 | 2.42 | 2.69 | 3.09 | 3.18 | 2.54 | 1.80 | 1.87 | 1.83 | 1.08 | 0.95 | 1.35 | 0.58 | 1.09 | 1.04 | 1.32 | 1.35 | 1.10 | 0.62 | 1.20 | 0.82 |
|  | Zardc22092(IAA12) | 2.01 | 3.95 | 2.88 | 6.30 | 6.76 | 7.30 | 7.30 | 4.44 | 6.24 | 5.48 | 5.60 | 4.82 | 3.00 | 2.81 | 2.95 | 3.58 | 3.73 | 2.53 | 2.91 | 4.15 | 5.71 | 5.03 | 4.81 | 6.01 |
|  | Zardc04224(IAA3) | 11.73 | 10.96 | 10.91 | 12.18 | 10.56 | 10.54 | 17.05 | 21.35 | 17.17 | 60.26 | 52.49 | 58.23 | 12.14 | 11.61 | 13.15 | 4.96 | 5.87 | 4.95 | 24.54 | 26.91 | 21.05 | 37.29 | 34.76 | 31.52 |
|  | Zardc45890(ARF7) | 9.50 | 9.66 | 9.00 | 13.03 | 11.95 | 13.33 | 10.86 | 9.88 | 10.84 | 8.77 | 10.26 | 11.24 | 5.37 | 5.34 | 5.48 | 6.05 | 5.92 | 4.05 | 6.84 | 6.44 | 6.58 | 5.77 | 5.69 | 4.74 |
|  | novel.721(SAUR53) | 0.99 | 0.80 | 2.04 | 3.48 | 0.77 | 3.60 | 0.37 | 1.05 | 0.47 | 0.55 | 0.32 | 0.86 | 3.35 | 2.92 | 1.81 | 0.71 | 0.47 | 0.65 | 2.33 | 3.75 | 2.24 | 1.08 | 0.33 | 0.68 |
|  | novel.4197(PRB1) | 10.10 | 9.63 | 9.60 | 7.40 | 9.95 | 7.55 | 2.73 | 5.10 | 4.57 | 1.00 | 1.42 | 1.31 | 0.47 | 0.11 | 0.76 | 0.00 | 1.07 | 0.33 | 0.53 | 0.48 | 1.01 | 0.77 | 1.00 | 0.58 |
|  | Zardc51803(AHK3) | 11.37 | 10.10 | 13.62 | 20.24 | 19.44 | 17.18 | 14.91 | 18.89 | 14.02 | 15.45 | 15.17 | 14.25 | 11.57 | 11.13 | 13.59 | 5.85 | 6.58 | 6.98 | 4.26 | 4.67 | 5.42 | 5.08 | 5.03 | 5.11 |
|  | Zardc04055(SPSB) | 2.84 | 2.63 | 2.76 | 2.15 | 1.51 | 1.68 | 1.20 | 1.17 | 1.01 | 0.47 | 0.57 | 0.52 | 3.49 | 3.95 | 4.33 | 0.37 | 0.45 | 0.44 | 2.09 | 1.84 | 3.48 | 2.42 | 3.30 | 2.63 |
|  | Zardc36683(SUS2) | 0.75 | 0.69 | 0.65 | 2.00 | 2.08 | 1.72 | 2.12 | 1.66 | 1.93 | 1.12 | 1.59 | 1.29 | 0.58 | 0.26 | 0.48 | 0.85 | 0.89 | 0.97 | 0.27 | 0.31 | 0.10 | 0.57 | 0.32 | 0.42 |
|  | Zardc44219 | 2.43 | 2.27 | 2.13 | 2.30 | 2.18 | 1.90 | 1.39 | 1.48 | 3.45 | 0.42 | 0.68 | 1.81 | 0.24 | 0.32 | 0.27 | 1.11 | 0.94 | 0.46 | 0.49 | 0.49 | 0.05 | 0.32 | 0.41 | 0.29 |
|  | Zardc21370(GH9B9) | 0.64 | 1.18 | 1.20 | 2.43 | 2.56 | 2.42 | 0.66 | 0.78 | 2.28 | 0.00 | 0.00 | 0.00 | 0.00 | 0.00 | 0.00 | 0.00 | 0.00 | 0.00 | 0.00 | 0.00 | 0.00 | 0.10 | 0.05 | 0.00 |
|  | Zardc11215(TPPD) | 1.20 | 1.05 | 0.81 | 1.59 | 0.83 | 0.97 | 1.06 | 1.01 | 0.13 | 5.32 | 7.36 | 6.84 | 0.25 | 0.12 | 0.23 | 0.22 | 0.32 | 0.12 | 0.35 | 0.19 | 0.40 | 0.41 | 1.53 | 1.17 |
|  | Zardc34000(BAM1) | 1.13 | 1.55 | 1.34 | 2.51 | 2.75 | 1.79 | 4.02 | 2.25 | 3.80 | 17.72 | 18.60 | 19.28 | 0.39 | 0.45 | 0.20 | 0.11 | 0.09 | 0.29 | 0.05 | 0.00 | 0.57 | 6.60 | 9.28 | 9.40 |
|  | novel.1016(BAM2) | 3.04 | 2.38 | 2.62 | 4.26 | 3.92 | 4.13 | 3.80 | 3.39 | 3.01 | 3.56 | 3.84 | 3.16 | 0.00 | 0.00 | 0.00 | 0.00 | 0.00 | 0.00 | 0.00 | 0.00 | 0.00 | 0.00 | 0.00 | 0.00 |
|  |  |  |  |  |  |  |  |  |  |  |  |  |  |  |  |  |  |  |  |  |  |  |  |  |  |
| **M3vs.F3** | Zardc46645(CKX3) | 141.94 | 130.74 | 136.04 | 56.24 | 58.75 | 56.37 | 92.41 | 97.63 | 98.51 | 17.64 | 16.73 | 17.39 | 128.71 | 128.10 | 139.26 | 1.60 | 1.45 | 1.57 | 3.32 | 3.40 | 3.09 | 1.29 | 0.98 | 0.73 |
|  | Zardc24534(CKX5) | 43.79 | 48.90 | 48.80 | 22.89 | 24.13 | 20.53 | 91.76 | 94.01 | 93.32 | 32.37 | 36.96 | 36.34 | 31.05 | 27.40 | 29.94 | 6.03 | 6.48 | 5.93 | 39.27 | 34.58 | 35.92 | 80.48 | 87.58 | 87.72 |
|  | novel.3902(CKX6) | 0.43 | 0.91 | 1.06 | 2.99 | 2.42 | 2.69 | 3.09 | 3.18 | 2.54 | 1.80 | 1.87 | 1.83 | 1.08 | 0.95 | 1.35 | 0.58 | 1.09 | 1.04 | 1.32 | 1.35 | 1.10 | 0.62 | 1.20 | 0.82 |
|  | Zardc19469(UGT85A4) | 19.38 | 15.52 | 18.84 | 15.56 | 14.07 | 13.47 | 14.89 | 16.94 | 9.84 | 11.41 | 11.05 | 12.38 | 9.84 | 8.34 | 12.32 | 9.63 | 11.83 | 10.44 | 6.30 | 5.94 | 6.69 | 9.83 | 9.29 | 8.44 |
|  | Zardc36944(CKX1) | 0.72 | 0.19 | 0.87 | 0.71 | 1.43 | 1.01 | 0.50 | 1.23 | 0.48 | 0.44 | 0.88 | 0.52 | 0.09 | 0.09 | 0.00 | 0.00 | 0.00 | 0.09 | 0.00 | 0.10 | 0.00 | 0.00 | 0.00 | 0.00 |
|  | Zardc01223(AHK2) | 33.95 | 38.01 | 43.65 | 34.19 | 34.60 | 28.08 | 26.67 | 24.09 | 29.76 | 32.72 | 37.82 | 28.40 | 43.33 | 38.03 | 41.14 | 31.54 | 40.34 | 34.60 | 5.85 | 4.95 | 14.19 | 9.94 | 11.57 | 11.76 |
|  | Zardc51803(AHK3) | 11.37 | 10.10 | 13.62 | 20.24 | 19.44 | 17.18 | 14.91 | 18.89 | 14.02 | 15.45 | 15.17 | 14.25 | 11.57 | 11.13 | 13.59 | 5.85 | 6.58 | 6.98 | 4.26 | 4.67 | 5.42 | 5.08 | 5.03 | 5.11 |
|  | Zardc55048(AHK4) | 64.68 | 63.69 | 63.13 | 81.23 | 75.89 | 77.47 | 96.48 | 94.62 | 91.84 | 79.00 | 77.78 | 79.51 | 69.82 | 67.52 | 67.53 | 71.74 | 73.57 | 71.04 | 42.38 | 41.13 | 44.37 | 49.75 | 49.67 | 50.61 |
|  | Zardc23082(AHK4) | 15.58 | 15.24 | 13.57 | 22.15 | 22.97 | 20.85 | 21.44 | 22.35 | 23.51 | 13.00 | 13.42 | 12.91 | 19.22 | 18.09 | 18.43 | 16.13 | 17.98 | 18.60 | 10.61 | 8.70 | 10.02 | 7.39 | 7.67 | 8.78 |
|  | Zardc33161(ARR9) | 42.00 | 38.58 | 44.80 | 57.50 | 66.25 | 59.89 | 58.50 | 65.76 | 58.48 | 39.41 | 36.07 | 37.32 | 32.92 | 30.16 | 34.04 | 40.36 | 43.14 | 42.47 | 28.20 | 32.36 | 23.07 | 33.27 | 26.46 | 27.36 |
|  | Zardc11274(ARR9) | 26.39 | 29.22 | 32.17 | 15.81 | 15.24 | 19.16 | 26.80 | 28.96 | 30.17 | 8.37 | 8.91 | 7.57 | 27.37 | 30.26 | 31.62 | 16.49 | 14.87 | 16.62 | 14.17 | 13.30 | 10.29 | 14.37 | 14.55 | 14.65 |
|  | Zardc14478(ARR24) | 2.09 | 2.56 | 1.48 | 1.31 | 1.71 | 2.91 | 3.37 | 4.63 | 4.69 | 3.39 | 1.86 | 1.29 | 1.19 | 1.86 | 0.92 | 0.35 | 0.40 | 0.56 | 1.78 | 0.81 | 2.36 | 3.15 | 0.94 | 2.55 |
|  | novel.4197(PRB1) | 10.10 | 9.63 | 9.60 | 7.40 | 9.95 | 7.55 | 2.73 | 5.10 | 4.57 | 1.00 | 1.42 | 1.31 | 0.47 | 0.11 | 0.76 | 0.00 | 1.07 | 0.33 | 0.53 | 0.48 | 1.01 | 0.77 | 1.00 | 0.58 |
|  | Zardc26158(GH3.1) | 1.05 | 1.12 | 1.41 | 1.51 | 1.93 | 1.44 | 4.02 | 4.77 | 6.00 | 0.84 | 0.75 | 0.90 | 0.88 | 0.91 | 1.48 | 0.85 | 0.85 | 0.79 | 2.27 | 2.27 | 2.23 | 1.89 | 2.11 | 1.58 |
|  | Zardc38825(TGA8) | 12.59 | 12.05 | 12.93 | 18.55 | 20.57 | 18.52 | 15.52 | 16.09 | 14.84 | 19.48 | 16.89 | 15.81 | 12.31 | 11.04 | 11.99 | 16.51 | 15.27 | 17.03 | 6.22 | 6.62 | 7.22 | 8.33 | 10.27 | 9.72 |
|  | Zardc27757 | 26.07 | 23.80 | 25.06 | 15.82 | 13.49 | 13.31 | 15.68 | 16.89 | 13.99 | 8.67 | 8.98 | 8.60 | 13.56 | 11.48 | 11.96 | 14.38 | 14.05 | 11.88 | 5.19 | 5.51 | 5.59 | 11.85 | 11.20 | 11.32 |
|  | novel.1016(BAM2) | 3.04 | 2.38 | 2.62 | 4.26 | 3.92 | 4.13 | 3.80 | 3.39 | 3.01 | 3.56 | 3.84 | 3.16 | 0.00 | 0.00 | 0.00 | 0.00 | 0.00 | 0.00 | 0.00 | 0.00 | 0.00 | 0.00 | 0.00 | 0.00 |
|  | Zardc36683(SUS2) | 0.75 | 0.69 | 0.65 | 2.00 | 2.08 | 1.72 | 2.12 | 1.66 | 1.93 | 1.12 | 1.59 | 1.29 | 0.58 | 0.26 | 0.48 | 0.85 | 0.89 | 0.97 | 0.27 | 0.31 | 0.10 | 0.57 | 0.32 | 0.42 |
|  | Zardc21370(GH9B9) | 0.64 | 1.18 | 1.20 | 2.43 | 2.56 | 2.42 | 0.66 | 0.78 | 2.28 | 0.00 | 0.00 | 0.00 | 0.00 | 0.00 | 0.00 | 0.00 | 0.00 | 0.00 | 0.00 | 0.00 | 0.00 | 0.10 | 0.05 | 0.00 |
|  | Zardc01536 | 9.70 | 8.74 | 9.95 | 5.96 | 6.75 | 6.02 | 12.88 | 7.77 | 10.35 | 8.03 | 7.74 | 10.06 | 7.79 | 7.62 | 9.83 | 9.79 | 9.44 | 10.47 | 5.40 | 4.84 | 4.87 | 7.87 | 8.30 | 6.89 |
|  | Zardc44219 | 2.43 | 2.27 | 2.13 | 2.30 | 2.18 | 1.90 | 1.39 | 1.48 | 3.45 | 0.42 | 0.68 | 1.81 | 0.24 | 0.32 | 0.27 | 1.11 | 0.94 | 0.46 | 0.49 | 0.49 | 0.05 | 0.32 | 0.41 | 0.29 |
|  | Zardc36911(BGLU17) | 0.34 | 0.10 | 0.28 | 0.28 | 0.00 | 0.20 | 0.74 | 1.01 | 0.82 | 0.24 | 0.42 | 0.33 | 0.10 | 0.28 | 0.05 | 0.04 | 0.05 | 0.05 | 0.22 | 0.05 | 0.05 | 0.19 | 0.05 | 0.15 |
|  | Zardc34000(BAM1) | 1.13 | 1.55 | 1.34 | 2.51 | 2.75 | 1.79 | 4.02 | 2.25 | 3.80 | 17.72 | 18.60 | 19.28 | 0.39 | 0.45 | 0.20 | 0.11 | 0.09 | 0.29 | 0.05 | 0.00 | 0.57 | 6.60 | 9.28 | 9.40 |
|  | novel.1081(TPS6) | 0.87 | 1.06 | 0.92 | 1.16 | 0.78 | 0.96 | 1.48 | 1.00 | 2.11 | 1.01 | 1.38 | 1.30 | 0.41 | 0.08 | 0.46 | 0.79 | 0.33 | 0.93 | 0.18 | 0.50 | 0.62 | 0.23 | 0.54 | 0.41 |
|  |  |  |  |  |  |  |  |  |  |  |  |  |  |  |  |  |  |  |  |  |  |  |  |  |  |
| **M4vs.F4** | Zardc02254(JAZ10) | 52.53 | 38.36 | 51.06 | 38.81 | 32.67 | 36.78 | 38.84 | 42.89 | 37.58 | 234.29 | 229.24 | 240.61 | 46.01 | 33.09 | 42.33 | 25.18 | 25.19 | 28.16 | 32.57 | 31.12 | 31.48 | 101.98 | 104.12 | 98.60 |
|  | Zardc26868(ARF3) | 15.84 | 16.04 | 15.33 | 29.23 | 26.83 | 25.92 | 16.83 | 17.37 | 18.05 | 29.79 | 30.15 | 30.69 | 15.92 | 17.80 | 15.60 | 24.85 | 24.74 | 26.90 | 16.93 | 16.53 | 18.08 | 12.26 | 12.57 | 13.38 |
|  | Zardc01223(AHK2) | 33.95 | 38.01 | 43.65 | 34.19 | 34.60 | 28.08 | 26.67 | 24.09 | 29.76 | 32.72 | 37.82 | 28.40 | 43.33 | 38.03 | 41.14 | 31.54 | 40.34 | 34.60 | 5.85 | 4.95 | 14.19 | 9.94 | 11.57 | 11.76 |
|  | Zardc16527(AHK3) | 3.23 | 3.56 | 3.17 | 4.29 | 4.74 | 4.02 | 6.74 | 4.83 | 5.43 | 6.35 | 6.11 | 5.49 | 6.57 | 5.24 | 6.98 | 3.96 | 4.20 | 3.94 | 2.73 | 3.46 | 4.32 | 3.29 | 3.12 | 2.26 |
|  | Zardc16532(AHK3) | 12.27 | 10.87 | 15.60 | 16.75 | 15.81 | 12.43 | 18.02 | 17.91 | 16.26 | 19.66 | 21.57 | 18.06 | 16.79 | 16.19 | 21.16 | 12.11 | 12.44 | 11.88 | 9.61 | 8.13 | 8.96 | 7.33 | 7.75 | 7.71 |
|  | Zardc51803(AHK3) | 11.37 | 10.10 | 13.62 | 20.24 | 19.44 | 17.18 | 14.91 | 18.89 | 14.02 | 15.45 | 15.17 | 14.25 | 11.57 | 11.13 | 13.59 | 5.85 | 6.58 | 6.98 | 4.26 | 4.67 | 5.42 | 5.08 | 5.03 | 5.11 |
|  | novel.168(AHP4) | 0.00 | 0.00 | 0.00 | 0.00 | 0.00 | 0.00 | 0.00 | 0.00 | 0.00 | 2.68 | 3.03 | 1.60 | 0.00 | 0.00 | 0.00 | 0.00 | 0.00 | 0.00 | 0.00 | 0.00 | 0.00 | 0.00 | 0.00 | 0.00 |
|  | Zardc54051(ARR12) | 1.27 | 1.21 | 0.86 | 7.12 | 7.48 | 8.70 | 5.29 | 5.51 | 5.16 | 8.44 | 8.68 | 9.06 | 1.74 | 2.36 | 2.32 | 6.18 | 5.01 | 6.57 | 4.36 | 3.04 | 4.19 | 1.73 | 1.61 | 1.90 |
|  | Zardc46700(ARR3) | 27.97 | 22.97 | 25.12 | 46.24 | 45.92 | 41.99 | 63.47 | 67.35 | 75.06 | 52.35 | 46.21 | 52.66 | 39.17 | 39.13 | 30.48 | 38.35 | 39.97 | 36.76 | 53.25 | 43.85 | 41.95 | 49.93 | 48.57 | 50.40 |
|  | Zardc22249(GH3.3) | 4.92 | 4.41 | 3.66 | 4.28 | 4.62 | 4.30 | 3.31 | 4.51 | 4.74 | 4.82 | 4.72 | 4.17 | 3.34 | 3.77 | 3.40 | 3.71 | 2.81 | 2.90 | 2.11 | 2.17 | 2.96 | 1.76 | 1.90 | 1.86 |
|  | Zardc52799(GH3.6) | 23.38 | 13.58 | 19.69 | 14.47 | 17.76 | 15.20 | 9.12 | 11.54 | 6.68 | 10.13 | 11.09 | 12.95 | 17.93 | 16.13 | 22.56 | 15.06 | 25.17 | 20.32 | 3.13 | 7.32 | 5.27 | 2.61 | 4.84 | 4.14 |
|  | Zardc51255(ARF5) | 3.16 | 3.14 | 3.55 | 4.43 | 4.25 | 4.46 | 3.84 | 2.83 | 2.54 | 3.37 | 4.16 | 2.95 | 5.03 | 3.52 | 4.64 | 3.50 | 3.47 | 2.93 | 2.64 | 1.36 | 3.29 | 1.36 | 1.20 | 1.57 |
|  | Zardc00852(JAZ2) | 6.89 | 6.29 | 5.65 | 2.91 | 3.92 | 3.19 | 2.67 | 3.78 | 3.45 | 11.76 | 10.92 | 10.32 | 5.15 | 3.85 | 9.75 | 4.01 | 2.84 | 3.78 | 3.14 | 4.27 | 3.83 | 2.88 | 5.74 | 7.39 |
|  | Zardc29777(IAA19) | 3.12 | 5.43 | 4.41 | 3.21 | 4.24 | 2.31 | 3.14 | 3.90 | 2.28 | 10.24 | 9.56 | 11.28 | 5.04 | 3.75 | 4.11 | 1.82 | 4.20 | 3.34 | 2.99 | 4.21 | 3.52 | 7.19 | 3.22 | 4.69 |
|  | Zardc03151(BOP2) | 3.16 | 3.57 | 3.64 | 3.35 | 5.56 | 4.22 | 2.24 | 2.87 | 2.06 | 2.98 | 1.88 | 2.10 | 1.75 | 2.03 | 2.19 | 1.62 | 1.72 | 4.11 | 1.04 | 1.31 | 1.67 | 1.11 | 0.93 | 1.12 |
|  | Zardc46645(CKX3) | 141.94 | 130.74 | 136.04 | 56.24 | 58.75 | 56.37 | 92.41 | 97.63 | 98.51 | 17.64 | 16.73 | 17.39 | 128.71 | 128.10 | 139.26 | 1.60 | 1.45 | 1.57 | 3.32 | 3.40 | 3.09 | 1.29 | 0.98 | 0.73 |
|  | Zardc36944(CKX1) | 0.72 | 0.19 | 0.87 | 0.71 | 1.43 | 1.01 | 0.50 | 1.23 | 0.48 | 0.44 | 0.88 | 0.52 | 0.09 | 0.09 | 0.00 | 0.00 | 0.00 | 0.09 | 0.00 | 0.10 | 0.00 | 0.00 | 0.00 | 0.00 |
|  | novel.3902(CKX6) | 0.43 | 0.91 | 1.06 | 2.99 | 2.42 | 2.69 | 3.09 | 3.18 | 2.54 | 1.80 | 1.87 | 1.83 | 1.08 | 0.95 | 1.35 | 0.58 | 1.09 | 1.04 | 1.32 | 1.35 | 1.10 | 0.62 | 1.20 | 0.82 |
|  | novel.5250(TPS11) | 0.93 | 1.36 | 1.11 | 0.85 | 1.34 | 0.77 | 3.11 | 3.82 | 3.45 | 18.47 | 17.36 | 19.33 | 1.90 | 2.37 | 2.50 | 1.60 | 1.26 | 1.27 | 3.04 | 3.33 | 3.46 | 7.72 | 8.63 | 8.33 |
|  | Zardc11215(TPPD) | 1.20 | 1.05 | 0.81 | 1.59 | 0.83 | 0.97 | 1.06 | 1.01 | 0.13 | 5.32 | 7.36 | 6.84 | 0.25 | 0.12 | 0.23 | 0.22 | 0.32 | 0.12 | 0.35 | 0.19 | 0.40 | 0.41 | 1.53 | 1.17 |
|  | Zardc30514(ADG1) | 0.07 | 0.22 | 0.21 | 0.11 | 0.25 | 0.05 | 0.16 | 0.06 | 0.13 | 0.09 | 0.23 | 0.16 | 0.09 | 0.14 | 0.09 | 0.02 | 0.08 | 0.18 | 0.06 | 0.04 | 0.12 | 0.02 | 0.02 | 0.02 |
|  | Zardc28200(BGLU43) | 2.21 | 1.14 | 0.72 | 0.61 | 0.74 | 1.25 | 2.31 | 1.17 | 1.84 | 2.31 | 3.00 | 3.10 | 1.16 | 0.60 | 1.07 | 0.45 | 0.39 | 0.84 | 1.44 | 0.39 | 0.28 | 0.84 | 1.21 | 1.14 |
|  | Zardc34000(BAM1) | 1.13 | 1.55 | 1.34 | 2.51 | 2.75 | 1.79 | 4.02 | 2.25 | 3.80 | 17.72 | 18.60 | 19.28 | 0.39 | 0.45 | 0.20 | 0.11 | 0.09 | 0.29 | 0.05 | 0.00 | 0.57 | 6.60 | 9.28 | 9.40 |
|  | Zardc36683(SUS2) | 0.75 | 0.69 | 0.65 | 2.00 | 2.08 | 1.72 | 2.12 | 1.66 | 1.93 | 1.12 | 1.59 | 1.29 | 0.58 | 0.26 | 0.48 | 0.85 | 0.89 | 0.97 | 0.27 | 0.31 | 0.10 | 0.57 | 0.32 | 0.42 |
|  | novel.1016(BAM2) | 3.04 | 2.38 | 2.62 | 4.26 | 3.92 | 4.13 | 3.80 | 3.39 | 3.01 | 3.56 | 3.84 | 3.16 | 0.00 | 0.00 | 0.00 | 0.00 | 0.00 | 0.00 | 0.00 | 0.00 | 0.00 | 0.00 | 0.00 | 0.00 |

**Supplementary Table 9 The detailed information of the DEGs related to MADS genes detected in this study**

| **Genes** | ***At.* ID** | ***At.* name** | **Blastx to TAIR11 database** |
| --- | --- | --- | --- |
| novel.4695 | AT5G62165 | AtFYF | regulation of flower development |
| novel.477 | AT5G20240 | AtPI | specification of floral organ identity |
| novel.478 | AT5G20240 | AtPI | specification of floral organ identity |
| Zardc02276 | AT5G13790 | AtAGL15 | negative regulation of flower development |
| Zardc08871 | AT4G22950 | AtAGL19 | positive regulation of transcription by RNA polymerase II |
| Zardc10218 | AT3G58780 | AtSHP1 | carpel development, plant ovule development |
| Zardc10299 | AT3G02310 | AtSEP2 | flower development, plant ovule development |
| Zardc16484 | AT2G03060 | AtAGL30 | pollen maturation, regulation of pollen tube growth |
| Zardc17043 | AT5G20240 | AtPI | specification of floral organ identity |
| Zardc17954 | AT3G58780 | AtSHP1 | carpel development, plant ovule development |
| Zardc19020 | AT2G03060 | AtAGL30 | pollen maturation, regulation of pollen tube growth |
| Zardc19106 | AT5G58890 | AtAGL82 | positive regulation of transcription by RNA polymerase II |
| Zardc26470 | AT2G34440 | AtAGL29 | regulation of transcription by RNA polymerase II |
| Zardc28170 | AT1G24260 | AtSEP3 | flower development, plant ovule development |
| Zardc28223 | AT5G58890 | AtAGL82 | positive regulation of transcription by RNA polymerase II |
| Zardc28804 | AT3G02310 | AtSEP2 | flower development, plant ovule development |
| Zardc30745 | AT2G22540 | AtSVP | negative regulation of flower development |
| Zardc30916 | AT4G36920 | AtAP2 | specification of floral organ identity |
| Zardc32032 | AT5G15800 | AtSEP1 | flower development, plant ovule development |
| Zardc32139 | AT3G66656 | AtAGL91 | regulation of transcription by RNA polymerase II |
| Zardc32140 | AT2G34440 | AtAGL29 | regulation of transcription by RNA polymerase II |
| Zardc33025 | AT5G62165 | AtFYF | floral organ senescence, regulation of flower development |
| Zardc33529 | AT4G09960 | AtSTK | carpel development, plant ovule development |
| Zardc39101 | AT1G69120 | AtAP1 | floral meristem determinacy |
| Zardc39213 | AT1G69120 | AtAP1 | regulates the expression of flowering time |
| Zardc41637 | AT3G54340 | AtAP3 | specification of floral organ identity of floral organ identity |
| Zardc41638 | AT3G54340 | AtAP3 | specification of floral organ identity of floral organ identity |
| Zardc41639 | AT3G54340 | AtAP3 | specification of floral organ identity of floral organ identity |
| Zardc41655 | AT3G54340 | AtAP3 | specification of floral organ identity of floral organ identity |
| Zardc41656 | AT3G54340 | AtAP3 | specification of floral organ identity of floral organ identity |
| Zardc42245 | AT3G54340 | AtAP3 | specification of floral organ identity of floral organ identity |
| Zardc42251 | AT3G54340 | AtAP3 | specification of floral organ identity of floral organ identity |
| Zardc42253 | AT3G54340 | AtAP3 | specification of floral organ identity of floral organ identity |
| Zardc44971 | AT4G09960 | AtSTK | carpel development, plant ovule development |
| Zardc51499 | AT5G62165 | AtFYF | floral organ senescence, regulation of flower development |
| Zardc51954 | AT2G45660 | AtSOC1 | positive regulation of flower development |
| Zardc02402 | AT4G18960 | AtAG | carpel development, stamen development |

**Supplementary Table 10 The data information to conduct the co-expression network analysis**

| **Name** | **Cluster** | **Degree** | **M3vsF3_log2FC** | **M3vsF3_log2FC** | **M3vsF3_pvalue** | **M4vsF4_pvalue** | **Root Mean Square** |
| --- | --- | --- | --- | --- | --- | --- | --- |
| novel.168(AHP4) | Phytohormone signal transduction | 24 | . | 7.10 | -- | 0.00 | 7.10 |
| novel.4197(PRB1) | Phytohormone signal transduction | 18 | 2.64 | 0.67 | 0.00 | 0.34 | 1.92 |
| Zardc00852(JAZ2) | Phytohormone signal transduction | 19 | -0.17 | 1.05 | 0.64 | 0.00 | 0.75 |
| Zardc01223(AHK2) | Phytohormone signal transduction | 11 | 1.70 | 1.57 | 0.00 | 0.00 | 1.64 |
| Zardc02254(JAZ10) | Phytohormone signal transduction | 26 | 0.34 | 1.21 | 0.02 | 0.00 | 0.89 |
| Zardc03151(BOP2) | Phytohormone signal transduction | 21 | 0.85 | 1.14 | 0.03 | 0.00 | 1.00 |
| Zardc22249(GH3.3) | Phytohormone signal transduction | 25 | 0.81 | 1.31 | 0.00 | 0.00 | 1.09 |
| Zardc23082(AHK4) | Phytohormone signal transduction | 14 | 1.21 | 0.72 | 0.00 | 0.00 | 1.00 |
| Zardc26158(GH3.1) | Phytohormone signal transduction | 5 | 1.14 | -1.17 | 0.00 | 0.00 | 1.16 |
| Zardc26868(ARF3) | Phytohormone signal transduction | 11 | 0.03 | 1.25 | 0.78 | 0.00 | 0.88 |
| Zardc29777(IAA19) | Phytohormone signal transduction | 22 | -0.19 | 1.04 | 0.68 | 0.00 | 0.75 |
| Zardc33161(ARR9) | Phytohormone signal transduction | 17 | 1.14 | 0.37 | 0.00 | 0.00 | 0.85 |
| Zardc38825(PAN) | Phytohormone signal transduction | 16 | 1.22 | 0.88 | 0.00 | 0.00 | 1.07 |
| Zardc51255(ARF5) | Phytohormone signal transduction | 9 | 0.35 | 1.34 | 0.36 | 0.00 | 0.98 |
| Zardc52799(GH3.6) | Phytohormone signal transduction | 9 | 0.80 | 1.56 | 0.07 | 0.00 | 1.24 |
| ABA | Plant hormone | 4 | -0.25 | -0.05 | 0.00 | 0.36 | 0.18 |
| ACC | Plant hormone | 6 | -0.30 | -0.24 | 0.45 | 0.58 | 0.27 |
| GA1 | Plant hormone | 1 | -2.88 | -3.45 | 0.00 | 0.00 | 3.18 |
| GA3 | Plant hormone | 3 | 0.11 | 0.00 | 0.50 | 0.99 | 0.08 |
| GA4 | Plant hormone | 5 | -0.21 | 0.07 | 0.54 | 0.85 | 0.16 |
| IAA | Plant hormone | 9 | 1.00 | -0.42 | 0.00 | 0.04 | 0.77 |
| JA | Plant hormone | 12 | -1.45 | 0.30 | 0.00 | 0.25 | 1.05 |
| JA-ILE | Plant hormone | 5 | -2.24 | 2.51 | 0.12 | 0.08 | 2.38 |
| OPDA | Plant hormone | 5 | -0.77 | -2.35 | 0.00 | 0.01 | 1.75 |
| SA | Plant hormone | 1 | -1.38 | -1.85 | 0.00 | 0.00 | 1.63 |
| TZ | Plant hormone | 22 | 3.42 | 2.22 | 0.00 | 0.00 | 2.88 |
| TZR | Plant hormone | 25 | 4.32 | 1.39 | 0.00 | 0.00 | 3.21 |
| novel.1016(BAM2) | Starch and sucrose metablism | 23 | 8.21 | 8.41 | 0.00 | 0.00 | 8.31 |
| novel.1081(TPS6) | Starch and sucrose metablism | 20 | 1.82 | 1.65 | 0.01 | 0.02 | 1.73 |
| novel.5250(TPS11) | Starch and sucrose metablism | 26 | 0.09 | 1.16 | 0.64 | 0.00 | 0.82 |
| Zardc01536 | Starch and sucrose metablism | 3 | 1.05 | 0.16 | 0.00 | 0.36 | 0.75 |
| Zardc11215(TPPD) | Starch and sucrose metablism | 31 | 1.23 | 2.65 | 0.16 | 0.00 | 2.07 |
| Zardc21370(GH9B9) | Starch and sucrose metablism | 17 | 6.94 | -2.45 | 0.00 | 0.45 | 5.20 |
| Zardc27757 | Starch and sucrose metablism | 17 | 1.53 | -0.39 | 0.00 | 0.01 | 1.11 |
| Zardc28200(BGLU43) | Starch and sucrose metablism | 25 | 1.36 | 1.40 | 0.09 | 0.01 | 1.38 |
| Zardc30514(ADG1) | Starch and sucrose metablism | 9 | 0.67 | 3.14 | 0.51 | 0.01 | 2.27 |
| Zardc34000(BAM1) | Starch and sucrose metablism | 29 | 4.05 | 1.14 | 0.00 | 0.00 | 2.98 |
| Zardc36683(SUS2) | Starch and sucrose metablism | 23 | 3.08 | 1.62 | 0.00 | 0.00 | 2.46 |
| Zardc36911(BGLU17) | Starch and sucrose metablism | 15 | 3.00 | 1.36 | 0.00 | 0.18 | 2.33 |
| Zardc44219 | Starch and sucrose metablism | 19 | 2.61 | 1.51 | 0.00 | 0.02 | 2.13 |
| Soluble_sugar | Sugar compounds | 24 | 0.67 | 0.12 | 0.03 | 0.67 | 0.48 |
| Sucrose | Sugar compounds | 27 | 1.49 | 0.42 | 0.00 | 0.01 | 1.09 |
| Total_sugar | Sugar compounds | 28 | 0.67 | 0.01 | 0.00 | 0.90 | 0.48 |
| novel.4695(ZaFYF) | Transcription factor | 22 | 8.02 | 6.68 | 0.00 | 0.00 | 7.38 |
| novel.477(ZaPI) | Transcription factor | 27 | 5.27 | 5.87 | 0.00 | 0.00 | 5.58 |
| novel.478(ZaPI) | Transcription factor | 29 | 4.51 | 5.66 | 0.00 | 0.00 | 5.11 |
| Zardc02276(ZaAGL15) | Transcription factor | 10 | -0.02 | -2.42 | 0.99 | 0.00 | 1.71 |
| Zardc08871(ZaAGL19) | Transcription factor | 2 | -0.57 | -1.01 | 0.00 | 0.00 | 0.82 |
| Zardc10218(ZaSHP1) | Transcription factor | 14 | 0.55 | -1.28 | 0.24 | 0.00 | 0.99 |
| Zardc10299(ZaSEP2) | Transcription factor | 24 | 0.74 | 0.78 | 0.00 | 0.00 | 0.76 |
| Zardc16484(ZaAGL30) | Transcription factor | 20 | -0.31 | 1.07 | 0.56 | 0.00 | 0.78 |
| Zardc16527(AHK3) | Transcription factor | 2 | 0.70 | 1.05 | 0.00 | 0.00 | 0.89 |
| Zardc17043(ZaPI) | Transcription factor | 30 | 3.61 | 4.73 | 0.00 | 0.00 | 4.21 |
| Zardc17954(ZaSEP1) | Transcription factor | 9 | -1.43 | -1.63 | 0.41 | 0.00 | 1.54 |
| Zardc19106(ZaAGL82) | Transcription factor | 2 | -1.70 | -0.98 | 0.00 | 0.00 | 1.39 |
| Zardc26470(ZaAGL29) | Transcription factor | 2 | -0.56 | -0.19 | 0.05 | 0.59 | 0.42 |
| Zardc28170(ZaSEP3) | Transcription factor | 10 | -2.07 | -2.12 | 0.04 | 0.00 | 2.10 |
| Zardc28223(ZaAGL82) | Transcription factor | 2 | -0.33 | -1.16 | 0.35 | 0.00 | 0.86 |
| Zardc28804(ZaSEP2) | Transcription factor | 28 | 0.42 | -0.24 | 0.05 | 0.00 | 0.34 |
| Zardc30745(ZaSVP) | Transcription factor | 3 | -0.39 | -1.11 | 0.25 | 0.00 | 0.83 |
| Zardc30916(ZaAP2) | Transcription factor | 6 | 0.35 | -1.11 | 0.00 | 0.00 | 0.82 |
| Zardc32032(ZaSEP1) | Transcription factor | 28 | 0.45 | -0.38 | 0.03 | 0.00 | 0.42 |
| Zardc32139(ZaAGL91) | Transcription factor | 11 | 0.85 | 1.40 | 0.80 | 0.39 | 1.15 |
| Zardc32140(ZaAGL29) | Transcription factor | 15 | 2.86 | 2.89 | 0.15 | 0.07 | 2.87 |
| Zardc33025(ZaFYF) | Transcription factor | 22 | 0.23 | 1.44 | 0.17 | 0.00 | 1.03 |
| Zardc33529(ZaSTK) | Transcription factor | 10 | . | -5.83 | -- | 0.00 | 5.83 |
| Zardc39101(ZaAP1) | Transcription factor | 26 | 1.27 | 1.05 | 0.00 | 0.00 | 1.16 |
| Zardc41637(ZaAP3) | Transcription factor | 28 | 4.15 | 2.02 | 0.04 | 0.01 | 3.26 |
| Zardc41638(ZaAP3) | Transcription factor | 28 | 4.62 | 1.64 | 0.01 | 0.02 | 3.47 |
| Zardc41639(ZaAP3) | Transcription factor | 30 | 4.71 | 0.52 | 0.01 | 0.45 | 3.35 |
| Zardc41655(ZaAP3) | Transcription factor | 27 | 0.63 | 2.10 | 0.58 | 0.00 | 1.55 |
| Zardc41656(ZaAP3) | Transcription factor | 29 | 1.89 | 2.12 | 0.09 | 0.00 | 2.01 |
| Zardc42245(ZaAP3) | Transcription factor | 27 | 1.19 | 3.08 | 0.49 | 0.00 | 2.33 |
| Zardc42251(ZaAP3) | Transcription factor | 27 | 2.47 | 1.47 | 0.04 | 0.00 | 2.03 |
| Zardc42253(ZaAP3) | Transcription factor | 29 | 3.95 | 2.02 | 0.00 | 0.00 | 3.14 |
| Zardc44971(ZaSTK) | Transcription factor | 9 | 2.39 | -5.86 | 0.51 | 0.00 | 4.47 |
| Zardc51499(ZaFYF) | Transcription factor | 14 | 0.54 | 0.42 | 0.05 | 0.23 | 0.49 |
| Zardc51954(ZaSOC1) | Transcription factor | 3 | -4.00 | -0.02 | 0.00 | 0.99 | 2.83 |
| novel.3902(CKX6) | Zeatin biosynthesis | 15 | 1.24 | 1.06 | 0.00 | 0.00 | 1.15 |
| Zardc19469(UGT85A4) | Zeatin biosynthesis | 22 | 1.15 | 0.34 | 0.00 | 0.13 | 0.85 |
| Zardc24534(CKX5) | Zeatin biosynthesis | 12 | 1.36 | -1.28 | 0.00 | 0.00 | 1.32 |
| Zardc36944(CKX1) | Zeatin biosynthesis | 20 | 4.37 | 5.22 | 0.01 | 0.00 | 4.81 |
| Zardc46645(CKX3) | Zeatin biosynthesis | 13 | 4.89 | 4.10 | 0.00 | 0.00 | 4.51 |
